# Reused Protein Segments Linked to Functional Dynamics

**DOI:** 10.1093/molbev/msae184

**Published:** 2024-09-03

**Authors:** Yiğit Kutlu, Gabriel Axel, Rachel Kolodny, Nir Ben-Tal, Turkan Haliloglu

**Affiliations:** Department of Chemical Engineering and Polymer Research Center, Bogazici University, Istanbul, Turkey; School of Neurobiology, Biochemistry & Biophysics, George S. Wise Faculty of Life Sciences, Tel Aviv University, Tel Aviv, Israel; Department of Computer Science, University of Haifa, Haifa, Israel; School of Neurobiology, Biochemistry & Biophysics, George S. Wise Faculty of Life Sciences, Tel Aviv University, Tel Aviv, Israel; Department of Chemical Engineering and Polymer Research Center, Bogazici University, Istanbul, Turkey

**Keywords:** structural dynamics, elastic network models, themes, evolution, domains, dynamic elements

## Abstract

Protein space is characterized by extensive recurrence, or “reuse,” of parts, suggesting that new proteins and domains can evolve by mixing-and-matching of existing segments. From an evolutionary perspective, for a given combination to persist, the protein segments should presumably not only match geometrically but also dynamically communicate with each other to allow concerted motions that are key to function. Evidence from protein space supports the premise that domains indeed combine in this manner; we explore whether a similar phenomenon can be observed at the sub-domain level. To this end, we use Gaussian Network Models (GNMs) to calculate the so-called soft modes, or low-frequency modes of motion for a dataset of 150 protein domains. Modes of motion can be used to decompose a domain into segments of consecutive amino acids that we call “dynamic elements”, each of which belongs to one of two parts that move in opposite senses. We find that, in many cases, the dynamic elements, detected based on GNM analysis, correspond to established “themes”: Sub-domain-level segments that have been shown to recur in protein space, and which were detected in previous research using sequence similarity alone (i.e. completely independently of the GNM analysis). This statistically significant correlation hints at the importance of dynamics in evolution. Overall, the results are consistent with an evolutionary scenario where proteins have emerged from themes that need to match each other both geometrically and dynamically, e.g. to facilitate allosteric regulation.

## Introduction

Analysis of protein space may reveal how proteins emerged and continue to evolve. In general, evolution leaves traces in the form of segments that are shared among proteins, where significantly similar segments can be assumed to have diverged from a common ancestor. The many instances of similarity across proteins in sequence and/or structure indicate that proteins evolve by “reusing” parts that provide evolutionary benefit (Shakhnovich et al. 1996; Holm and Sander 1999; Chothia et al. 2003; Socolich et al. 2005; Lee et al. 2007; Liberles et al. 2012; Nepomnyachiy et al. 2014, 2017; Edwards and Deane 2015; Alva and Lupas 2018). Protein domains—as defined, e.g. in the SCOP, CATH, CDD, and ECOD databases (Murzin et al. 1995; Orengo et al. 1997; Marchler-Bauer et al. 2011; Cheng et al. 2014)—are perhaps the best-known example of segments that recur across multiple proteins. Domains, in turn, seem to have emerged and evolved through reuse of even shorter protein segments (Eck and Dayhoff, 1966; Berezovsky et al. 2000; Lupas et al. 2001; Trifonov et al. 2001; Dokholyan et al. 2002; Alva et al. 2015; Raanan et al. 2018; Longo et al. 2020; Raanan et al. 2020; Ben-Tal and Lupas 2021; Kolodny et al. 2021; Qiu et al. 2022).

If we consider protein evolution as a process in which existing protein segments are “mixed and matched” into new combinations, it seems reasonable to assume that segments corresponding to parts of different functions should render a combined function. However, for such a combination to persist from an evolutionary perspective, it must satisfy various physicochemical considerations: First and foremost, there must be a geometric fit between the individual parts, and, in addition, their dynamic behaviors must match. Evidence from protein space supports this premise: for example, reusing a membrane-binding PH domain in a kinase chain can bring the latter closer to its membrane-embedded substrate. However, to facilitate allostery between membrane binding and catalysis, the PH and kinase domains should dynamically couple to each other (Chu et al. 2020). As another example, in ABC transporters, docking of the substrate-binding protein at the extracellular side is dynamically coupled with ATP binding and hydrolysis at the intracellular nucleotide binding domains (NBDs) 60 to 70 Å away (Acar et al. 2020). Notably, these examples correspond to the above-domain level. Here, using a computational approach, we explore whether protein space supports the existence of such a phenomenon at the *sub-domain* level. More specifically, we seek to obtain evidence that evolutionarily conserved sub-domain-level segments “mix and match” into combined dynamic behavior.

To this end, we first identify dynamic protein regions that are likely to manifest such a composition. Specifically, we consider a set of 150 ECOD domains, in which we have previously and independently detected multiple (sub-domain-level) fragments that are reused across protein space; such fragments are referred to as “themes” (Nepomnyachiy et al. 2017; see below for further details). We focus on the ECOD domains’ so-called slow/soft modes of motion: Cooperative conformational motions that are robustly favored by the protein's structure and can be reliably predicted by normal mode analysis (Alexandrov et al. 2005; Bahar and Rader 2005; Fuglebakk et al. 2015; Haliloglu and Bahar 2015; Grudinin et al. 2020). These motions are made possible by the intrinsic dynamics of the protein and are essential to protein function; indeed, prior studies have used normal mode analysis to relate intrinsic dynamics to protein–substrate interactions, binding, catalysis, and allosteric responses (Henzler-Wildman et al. 2007; Li et al. 2014; McClendon et al. 2014; Soner et al. 2015; Chandrasekaran et al. 2016; Chopra et al. 2016; Mishra and Jernigan 2018; Tiwari and Reuter 2018; Guclu et al. 2021). The fact that intrinsic dynamics and function are linked implies that the former is also subject to evolutionary selection (Maguid et al. 2006; Bastolla et al. 2017; Zhang et al. 2020). Indeed, normal mode analyses show that evolutionarily related proteins share similar global dynamics (Maguid et al. 2005; Bastolla et al. 2017; Campitelli et al. 2020). Residues in critical positions, and in particular residue pairs that mediate allosteric communication, are evolutionarily conserved (Hatley et al. 2003; Süel et al. 2003; Granata et al. 2017). For specific folds, analyzing the normal modes of ancestrally reconstructed proteins offered hypotheses as to how the dynamic behavior itself evolved (Campitelli et al. 2020; Modi et al. 2021). Notably, those studies considered evolutionary relationships among domains; we focus on the evolutionary signal at the sub-domain level and study its relationship to dynamic behavior.

We use elastic network analysis with the Gaussian Network Model (GNM) to identify the soft/slow modes of motion of each ECOD domain in our dataset. In GNM, proteins are represented as a collection of interaction sites, corresponding to their amino acids, with springs between those that are sufficiently close to each other in 3D space (Bahar et al. 1997; Haliloglu et al. 1997; Emekli et al. 2008). With the aid of such a representation, for each of the slow modes of motion, we obtain a decomposition of the corresponding domain into geometrically compact units—where each unit comprises amino acids that move in a coordinated fashion. We then divided these compact units into amino acid segments that are continuous in sequence (as explained below) and refer to each as a “dynamic element” (DE). We subsequently examine how the reused protein segments (“themes”) contained in the ECOD domain map onto these DEs (Nepomnyachiy et al. 2017).

As noted above, the reused protein segments contained in our focal ECOD domains were identified in a previous, independent study (Nepomnyachiy et al. 2017), in which we generated an extensive database of segments that recur across protein space. The process for detecting segment recurrence resembled the hidden Markov model (HMM)-based approach used (among other approaches) to classify proteins in the Pfam database (Finn et al. 2014; Mistry et al. 2021)—a database in which proteins are grouped into families on the basis of sequence similarity. Starting from a list of seeds (different from the seeds used to generate Pfam families), we utilized the HHsearch sequence search engine (Soding 2005) to systematically survey reuse in protein space, including that at the sub-domain level (Nepomnyachiy et al. 2017); we identified segments of at least 35 amino acids that recur across proteins. Like reused domains and Pfam entries, instances of recurring segments were not necessarily identical; rather, their sequences were (statistically significantly) similar. We called these shared segments “themes”—a term that reflects both their recurrent nature and the potential for diversity alongside such recurrence (“variations on a theme”). We emphasize that, like Pfam entries, the reused themes were identified based on their sequence similarity (as opposed to structure). As alluded to above, sub-domain shared themes may be remnants of ancient evolutionary events, from even before domains were formed.

**Fig. 1.**
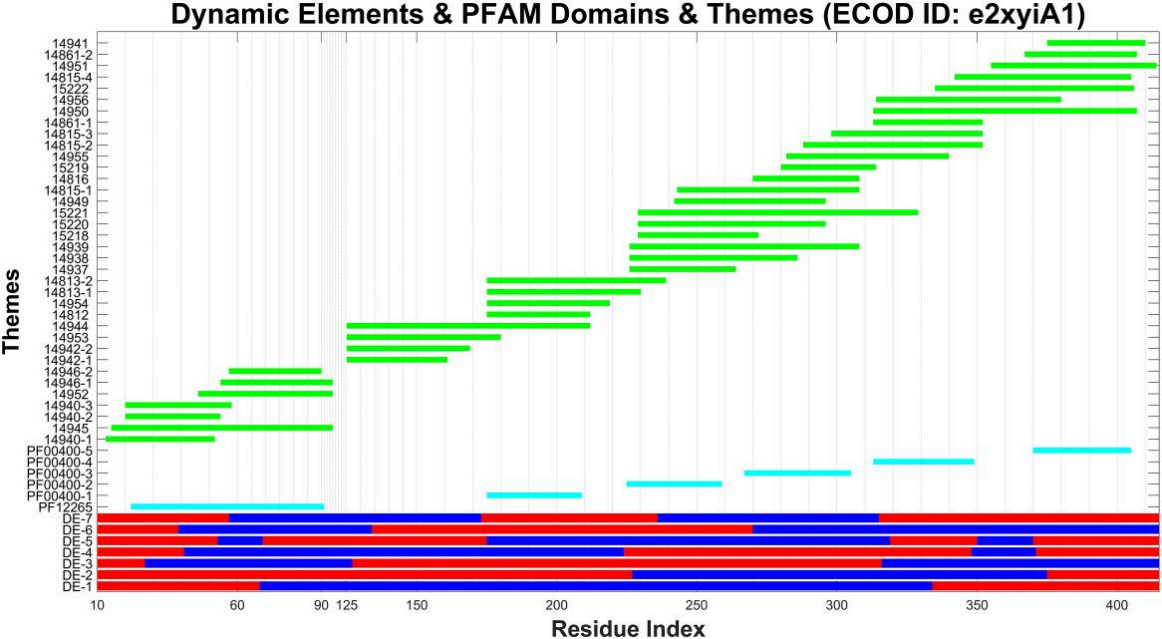
Correlation between shared protein segments and structural dynamics. The DEs of the e2xyiA1 propeller (from the histone-binding protein CAF1; PDB ID: 2XYI), in each of the seven slowest modes (DE-1 through DE-7), are marked in blue and red (corresponding to the opposite senses) along the residue index (*x* axis). The recurring sequence segments, “themes”, are marked along the *y* axis, and their positions are highlighted in green. Mappings of Pfam entries—also based on sequence data—are highlighted in cyan.

An important difference between themes and domains (or Pfam entries) is that themes are not mutually exclusive within a larger protein sequence, but rather can overlap. In other words, whereas domains and Pfam entries partition the protein chain into separate sets of residues, themes can overlap, such that an amino acid segment in a protein sequence may belong to any number of overlapping themes (Fig. 1). The assumption of overlap allowed us to expose reuse in its full magnitude, where complicated behavior was manifested—e.g. cases in which the same short amino acid segment belongs both to a long theme (shared by closely related proteins) and to a shorter theme (shared by more remotely related proteins). Recently, we have studied themes that mediate binding to the ancient ligand adenine (Narunsky et al. 2020), and that are shared among domains that are seemingly evolutionarily distinct, including very ancient ones (Longo et al. 2020; Kolodny et al. 2021; Qiu et al. 2022).

In what follows, we first present an in-depth analysis of 13 ECOD domains in our dataset—8 repeat domains and five non-repeat domains—belonging to six different homology groups. For these domains, we show correspondence between the boundaries of the DEs (for each mode of motion) and the boundaries of the themes, detected purely based on sequence similarity. To reinforce and quantify this observation, we subsequently carry out a mutual information (MI) analysis, as well as a *P*-value analysis, of the complete set of 150 ECOD domains, belonging to 26 different homology groups. This analysis shows the statistical significance of the correspondence between themes and DEs. The correspondence we reveal describes a tangible link between dynamics and the evolution of domains.

## Results

### Data and Approach

**Fig. 2.**
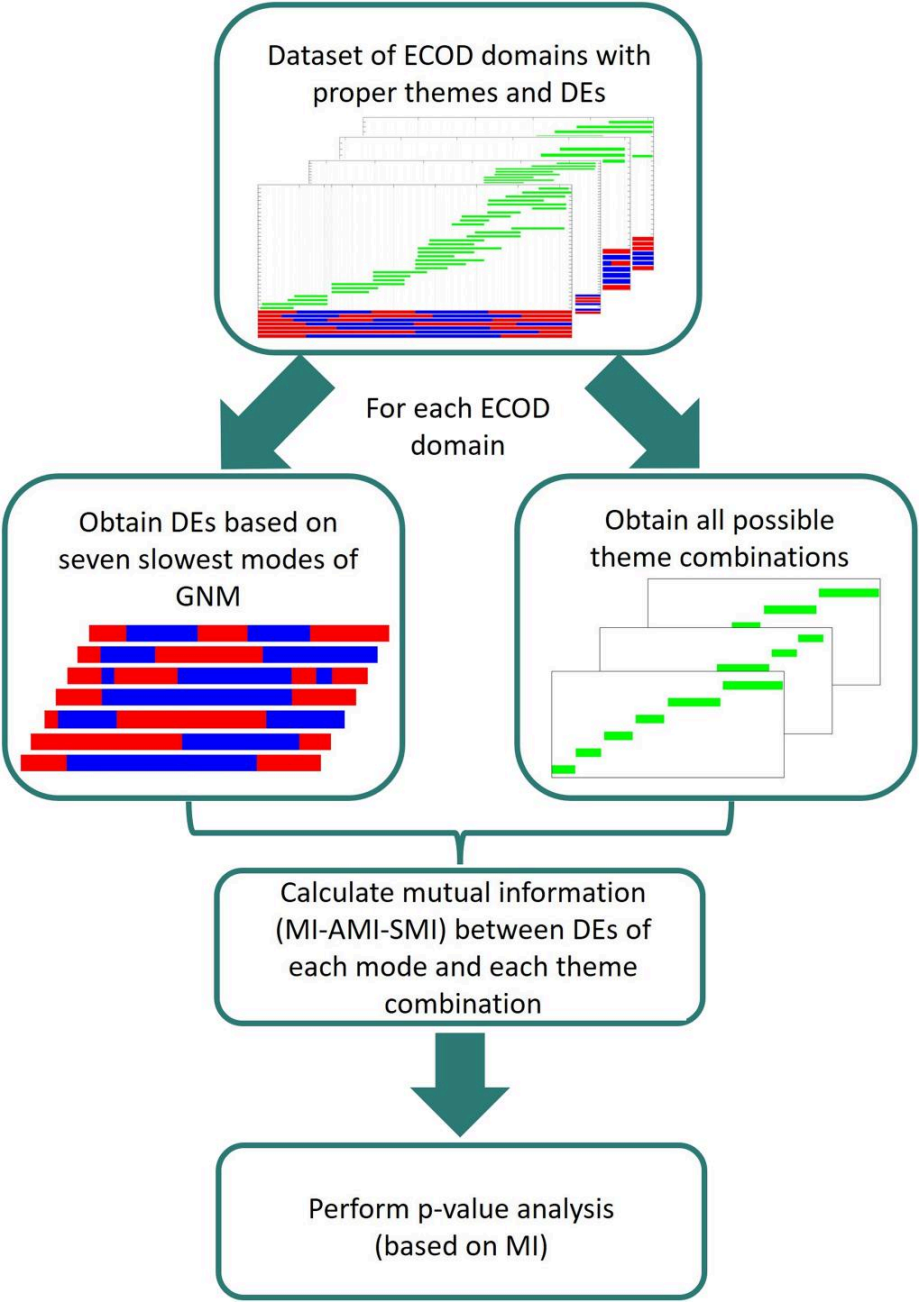
Schematic representation of the approach used here to examine the correlation between themes and dynamic elements.

A flowchart of the approach used is provided in Fig. 2. As noted above, our analyses focused on a set of 150 ECOD domains (see Supplementary website for the complete set). All the calculations in this study are performed on these ECOD domain regions (rather than whole PDB structures). We conducted two main sets of analyses:

1. an in-depth analysis on a small subset of 13 ECOD domains (the “in-depth set”), in which we examined possible correlations between themes and dynamics on a case-by-case basis; this dataset comprised eight repeat domains (Table 1) and five non-repeat domains (Table 2); and
2. a broader statistical analysis of the complete set of 150 ECOD domains (the “expanded set”), in which we sought to examine the statistical significance of the correlations observed in our in-depth set.

**Table 1:** A set of eight ECOD domains from four ECOD H-groups, and three very different architectures, all-beta, all-alpha, and alpha/beta (in-depth set repeats)

PDB ID	Domain ID	H-group name	T group name	Protein name
**2XYI**	e2xyiA1	Beta-propeller	7-bladed	Probable histone-binding protein CAF1
**3EMH**	e3emhA1			WD repeat-containing protein 5
**2OF3**	e2of3A1	ARM-repeat	ARM-repeat	ZYG-9
**1B3U**	e1b3uA1			Protein phosphatase PP2A
**4ADY**	e4adyA2	Proteasome/cyclosome (PC) repeat	Proteasome/cyclosome (PC) repeat	26s proteasome regulatory subunit RPN2
**1J6O**	e1j6oA1	TIM barrels	TIM barrels	TatD-related deoxyribonuclease
**2GZX**	e2gzxA1			Putative TatD-related DNAse
**4P5U**	e4p5uA1			Tat-linked quality control protein TatD

The PDB IDs of the corresponding proteins are listed on the left column, and the ECOD ID and annotation of the domain are listed in the next columns.

**Table 2:** A set of five ECOD domains from RIP and Rossmann-related ECOD H-groups (in-depth set nonrepeats)

PDB ID	Domain ID	H-group name	T group name	Protein name
**3KTZ**	e3ktzA1	Ribosome inactivating proteins (RIP)	Ribosome inactivating proteins (RIP)	Ribosome-inactivating protein gelonin
**3CTK**	e3ctkA1			rRNA N-glycosidase
**1ULS**	e1ulsB1	Rossmann-related	NAD(P)-binding Rossmann-fold domains	Putative 3-oxoacyl-acyl-carrier-protein reductase
**2AE2**	e2ae2A1			Tropinone reductase-II
**3N74**	e3n74B1			3-Ketoacyl-(acyl-carrier-protein) reductase

The PDB IDs of the corresponding proteins are listed on the left column, and the ECOD ID and annotation of the domain are listed in the next columns.

For each domain in our dataset, we used GNM to approximate the domain's equilibrium dynamics based on its 3D structure (Bahar et al. 1997; Haliloglu et al. 1997). In this model, the dynamics of a protein of *n* amino acids is represented as a spectrum of (*n* − 1) orthogonal modes of motion. The modes of motion are sorted based on their contributions, from the slowest (the so-called soft modes) to the fastest, such that mode-1 corresponds to the most collective global motion and mode *n* − 1 to the most local fluctuations. We focused on the seven slowest modes of collective motion, as explained in the “Materials and Methods” section. Each mode of motion partitions the amino acids in the domain into two dynamic parts, which move in opposite “senses”—meaning that the motions of the amino acids in one dynamic part are positively correlated with each other (within that specific mode), and are negatively correlated with the motions of the amino acids in the other dynamic part (Emekli et al. 2008). As described in the “Materials and Methods” section, we applied a filter to the results to obtain segments of at least 15 consecutive amino acids with correlated motion. These segments are predicted to move in a coordinated fashion, and we refer to them as DEs. Namely, DEs are consecutive subsets of the structural domains. Figure 1 shows the DEs for the seven slowest modes of the e2xyiA1 propeller projected along its amino acid sequence (We acknowledge that the most common approach to study protein dynamics is full atom molecular dynamics (MDs) simulations. However, as we demonstrate in “Comparison of DEs with GNM and MD simulations” below, revealing dynamics in atomic detail is an unnecessary burden for this study.).

After identifying the DEs in each of the seven slowest modes of motion for each ECOD domain, we examined the correspondence between the DEs and the (previously identified) themes contained in the domain (Nepomnyachiy et al. 2017). Visual representations of the DEs and themes observed for each domain, presented as bar graphs and PyMOL sessions, are available in the Supplementary website. We note that the partition into DEs depends on the GNM analysis, which is sensitive to the specific domain conformation used. We investigated the potential influence of conformation choice on our results; a search revealed that among the 150 ECOD domains in our dataset, only five had more than one conformation (with moderate differences between conformations, RMSD of over 2.5 Å). Reassuringly, in-depth analysis of two of these ECOD domains showed that the differences in the partitioning into DEs have little effect on the correlations with the themes (supplementary fig. S1, Supplementary Material online and supplementary tables S1 and S2, Supplementary Material online) (For the remaining three domains, such analysis was not feasible, as elaborated in the supplementary text, Supplementary Material online.).

### In-depth Analysis of Repeat Domains

We first conducted in-depth analysis for the set of eight ECOD domains with symmetrical repeat architectures (Table 1). This set comprised two (homologous all-β) propeller structures, two (homologous all-α) ARM-repeats, a repetitive alpha hairpin, and three (homologous α/β) TIM barrels.

#### All-β Architecture: Propellers

First, we considered two homologous seven-bladed beta-propellers, sharing 24% sequence identity (of similar structures: Superimposition RMSD of 3.26 Å): ECOD domain e2xyiA1 from histone-binding protein Chromatin Assembly Factor 1 (CAF1, Nurf55 in complex with an H4 peptide; PDB ID: 2XYI); and ECOD domain e3emhA1 from WD repeat-containing protein 5 (PDB ID: 3EMH). Figure 1 and supplementary fig. S2, Supplementary Material online show the themes and predicted DEs in the seven slowest modes of motion of the two ECOD domains; supplementary tables S3 and S4, Supplementary Material online list the themes for e2xyiA1 and e3emhA1, respectively. Each mode of motion comprises two dynamic parts (marked by red and blue) that move in opposite senses around hinges. Each of the two slowest modes of motion in e2xyiA1 and e3emhA1 feature at most three relatively long DEs. The higher modes feature more DEs, which, in turn, typically stretch over shorter segments. Within a given mode, each amino acid can belong only to one DE. However, when considering all (seven) slowest modes, the same amino acid belongs to many (seven) DEs. In this respect, the modes of motion are intertwined, just like the themes.

**Fig. 3.**
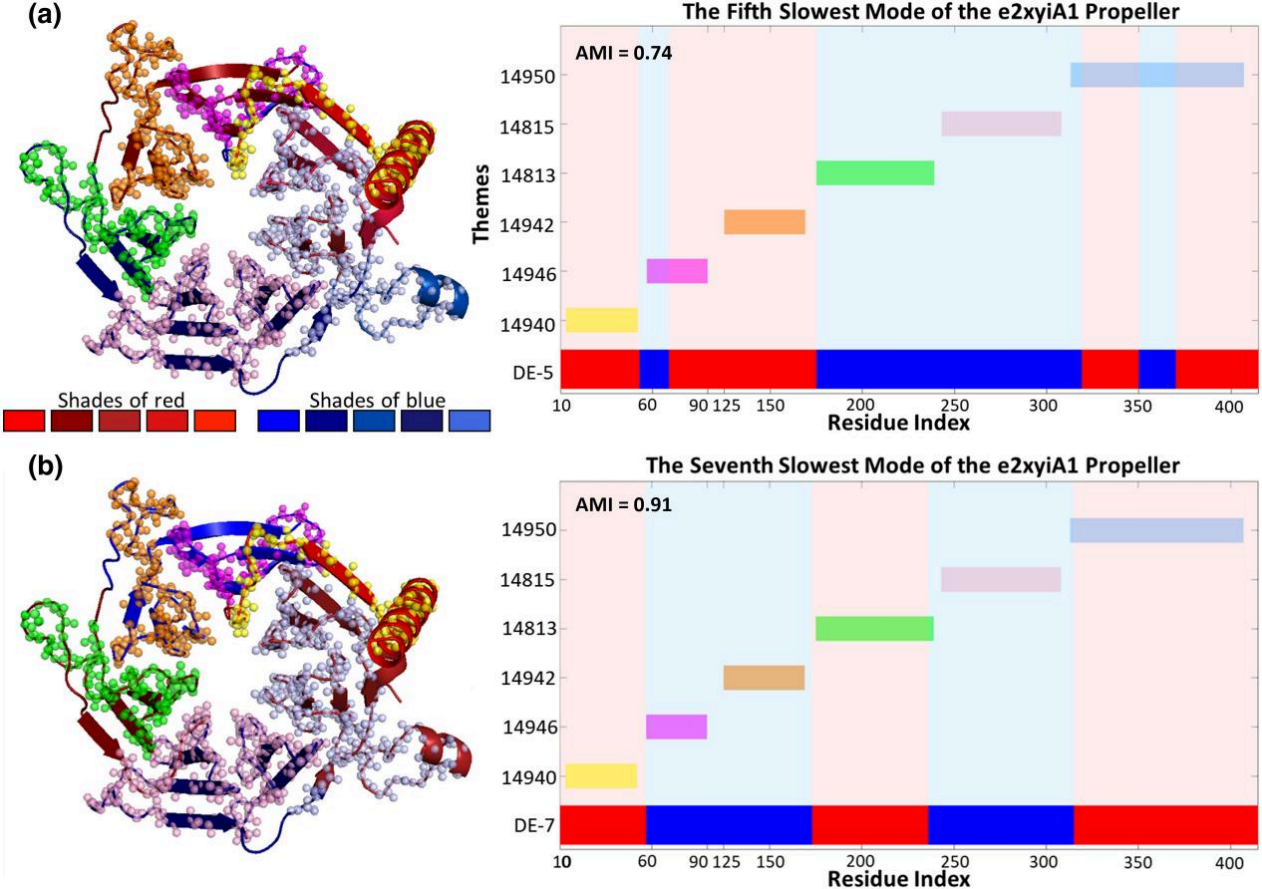
Correspondence between (sequence-based) themes and functional dynamics in the e2xyiA1 propeller (histone-binding protein CAF1, PDB ID: 2XYI). (Left) Themes projected on the 3D structure of the e2xyiA1 (β-propeller) domain of the histone-binding protein CAF1 (PDB ID: 2XYI) together with the DEs of the fifth (a) and seventh (b) modes of motion. The DEs were colored in shades of red and blue by their order of appearance in the sequence using the (arbitrarily chosen) palettes shown. The same DE coloring scheme was used also in the subsequent figures. (Right) Projection of the same data on the protein sequence. DEs (red and blue) and themes (various colors) are colored accordingly in both 3D and bar representations. While two themes constitute a DE in one mode, they could align with two different DEs in another mode.

In the propellers e2xyiA1 and e3emhA1, most of the DEs align with either a single theme or a combination of several reused themes. The dynamic segments of the third mode of e3emhA1 are an exception, as they are too short to allow decomposition into DEs with our 15 amino acid threshold (as explained in the “Materials and Methods” section below). We see different ways to combine themes, which correspond to the DEs of the different modes. This suggests a (possibly hierarchal) rewiring of the themes to facilitate functional motions. Figure 3 shows an example of selections of nonoverlapping themes that collectively cover, in essence, the entire e2xyiA1 propeller and correspond to the DEs of the fifth (Fig. 3a) and seventh (Fig. 3b) modes. Interestingly, in some cases there is a one-to-one correspondence between a DE and a theme, while in other cases two (or more) themes combine to cover a single DE, as in the case of the fourth DE of the fifth mode and the second DE of the seventh mode. Likewise, the same theme pair could align together with a single DE in one mode while each of them separately aligns with other DEs in another mode. In fact, to partition the protein domain e2xyiA1 so that the (sufficiently long) segments would fall along all the boundaries of DEs in both the fifth and seventh modes, one must use at least six segments. Indeed, the reused themes shown in the figure for e2xyiA1 are exactly these segments.

**Fig. 4.**
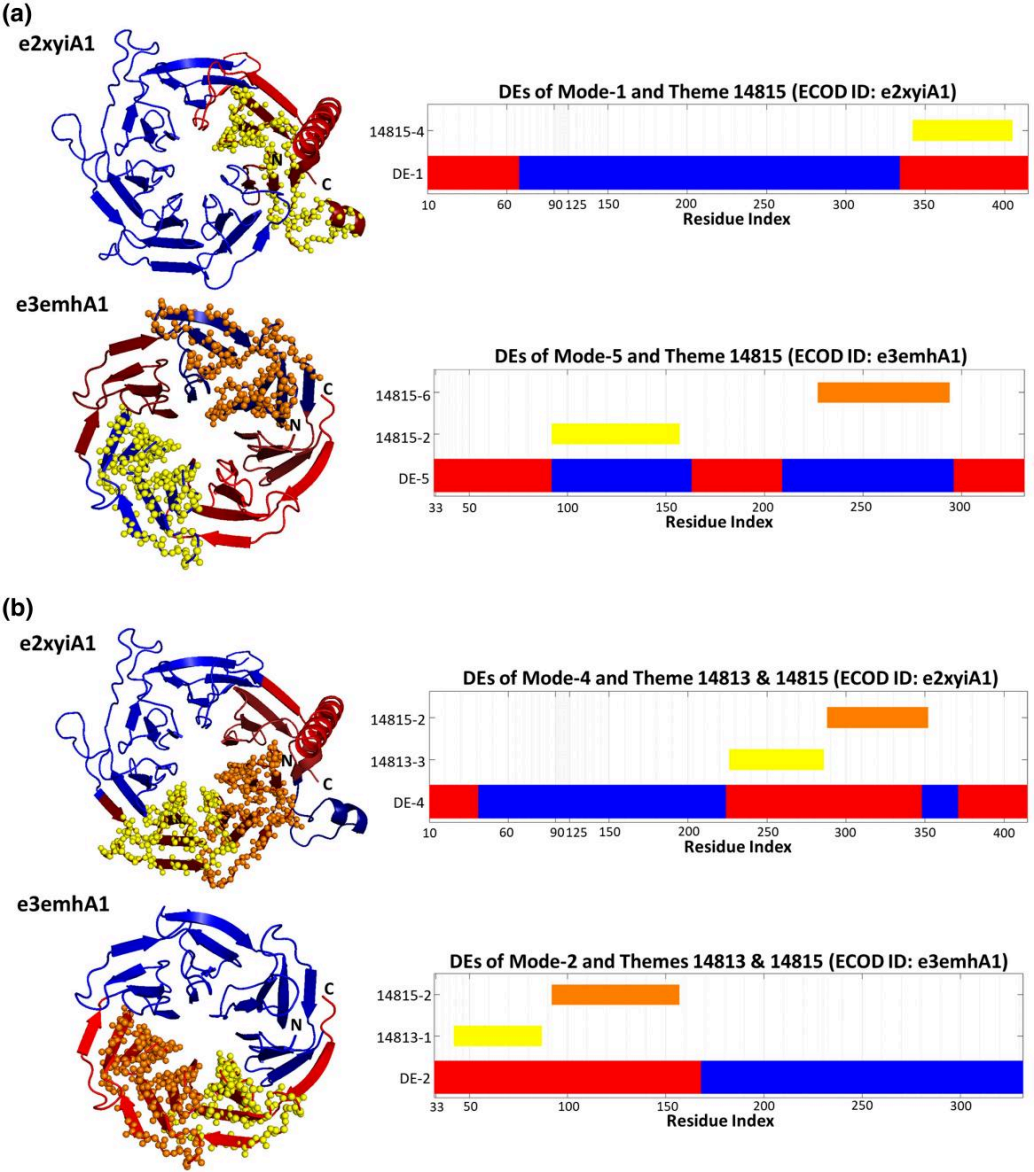
The same theme (or themes combination) may correspond to DEs of different modes of motion. a) Variations of theme 14815 correspond to DEs in the e2xyiA1 and e3emh propellers. The 14815-4 variation corresponds to one of the DEs of the first mode (yellow) of e2xyiA1, and variations 14815-2 and 14815-4 correspond to DEs of the fifth mode (orange and yellow, respectively) of e3emh. The red DE at the N-terminal of the first mode of e2xyiA1 corresponds to theme 14940 (not shown). b) A concatenation of themes 14813 and 14815 corresponds to a DE in the fourth mode of e2xyiA1, and second mode of e3emh. The two structures are shown from similar views. The themes are shown as yellow and orange spheres on the 3D structures on the left, and their sequences’ positions are marked by the bars on the right, and the DEs are mapped on the structure as in Fig. 3.

Sometimes equivalent themes of the two propeller domains match with DEs from different modes of motion. For example, theme 14815 corresponds to a DE of mode-1 for e2xyiA1 and a DE of mode-5 for e3emhA1 (Fig. 4a). Note that variations of theme 14815 (indexed 14815-1 through 14815-4) appear in multiple positions along the two propeller domains. Specifically, variation 14815-4 corresponds to a DE of mode-1 of e2xyiA1, and variations 14815-2 and 14815-4 correspond to DEs of mode-5 of e3emhA1. In another example, (variations of) themes 14813 and 14815 jointly correspond to a DE of mode-4 of e2xyiA1, and to a DE of mode-2 of e3emhA1 (Fig. 4b).

#### Themes, Dynamics, and Binding Sites

**Fig. 5.**
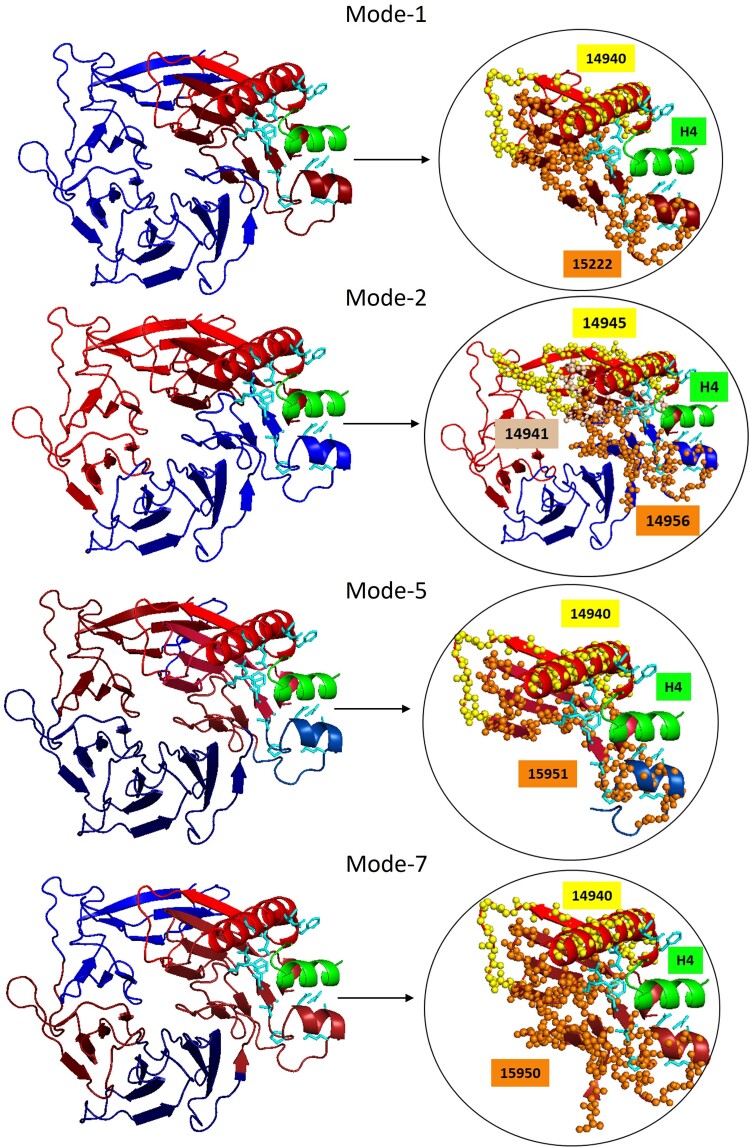
Correspondence between binding, dynamics, and themes in the e2xyiA1 (β-propeller) domain of the histone-binding protein CAF1 (PDB ID: 2XYI). The first, second, fifth, and seventh slowest modes of motion observed in the e2xyiA1 domain in complex with the histone H4 peptide. The H4 peptide is shown in green, and the DEs are colored in shades of red and blue (as in Fig. 3) in the respective dynamic parts with the sidechains of amino acids that bind histone marked with cyan sticks. Zoom-in views on the binding region illustrate the DEs and themes that mediate peptide binding. Themes that appear to mediate histone binding are represented in yellow and orange spheres.

For e2xyiA1, which is the histone-binding domain of CAF1, we know the histone peptide H4 binding site, as the protein's crystal structure is of the bound structure (Nowak et al. 2011). The dynamic dissection of e2xyiA1 shows that the H4 binding site is at the interfaces of the DEs of several slow modes of motion (Fig. 5, Supplementary website [session file]), demonstrating the importance of the DEs to H4 binding. The relationship between the boundaries of these DEs and themes at the histone-binding site suggests evolutionary links between dynamics and histone-binding in CAF1. The H4 binding site residues are encompassed by two themes (14940 and 15222) in the slowest mode, three themes (14945, 14956, and 14941) in the second slowest mode, two themes (14940 and 14951) in the fifth slowest mode, and two themes (14940 and 14950) in the seventh slowest mode (Fig. 5). Themes 14940 and 14945 repeatedly appear and partner with different themes in different modes to accompany the motion defined by each slow mode. Similarly, another histone binding site (histone H3) projected from a different crystal structure (PDB ID: 2YBA, ECOD Domain ID: e2ybaA1) is also located at the DE interfaces of e2xyiA1 (Supplementary website [session file]). That the same DEs mediate both H3 and H4 histone binding suggests allosteric communication between the corresponding binding sites. Some of the themes are shared between both binding sites (supplementary fig. S3, Supplementary Material online), suggesting possible evolutionary roots of the allostery in this case. The correspondence between DEs, themes, and binding sites supports the idea that dynamics is evolutionarily advantageous (perhaps by promoting allostery; see also (Ma et al. 2011; Liu and Bahar 2012; McClendon et al. 2014; Haliloglu and Bahar 2015; Chopra et al. 2016; Berezovsky et al. 2017; Mishra and Jernigan 2018; Saavedra et al. 2018; Guclu et al. 2021; Kutlu et al. 2021; Tang and Kaneko 2021).

#### All-α Architecture: Alpha Helix Bundles

**Fig. 6.**
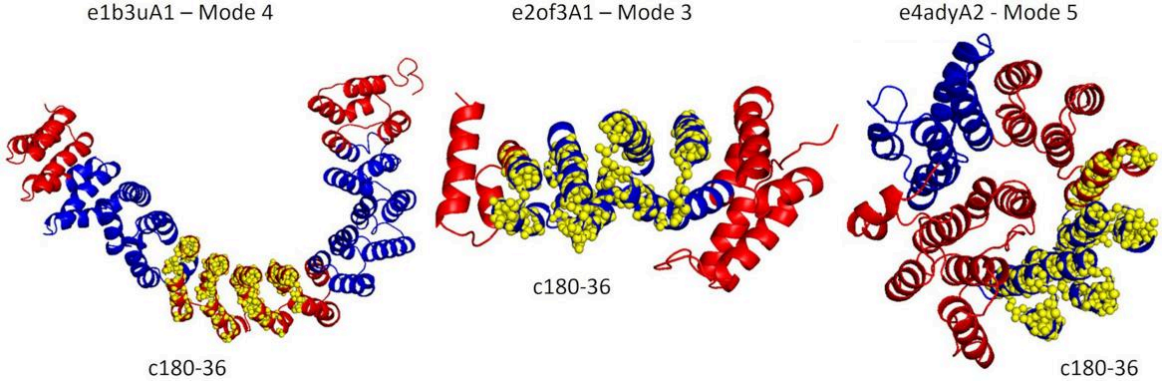
Theme c180-36 approximately corresponds to DEs in the fourth mode of e1b3uA1 (PP2A, PDB ID: 1B3U), the third mode of e2of3A1 (ZYG-9, PDB ID: 2OF3), and the fifth mode of e4adyA2 (26S proteasome subunit Rpn2, PDB ID: 4ADY). The theme is represented as yellow spheres, and the DEs in blue and red. Projections of the modes and themes on the protein sequence are presented in supplementary figs. S4 to S6, Supplementary Material online.

**Fig. 7.**
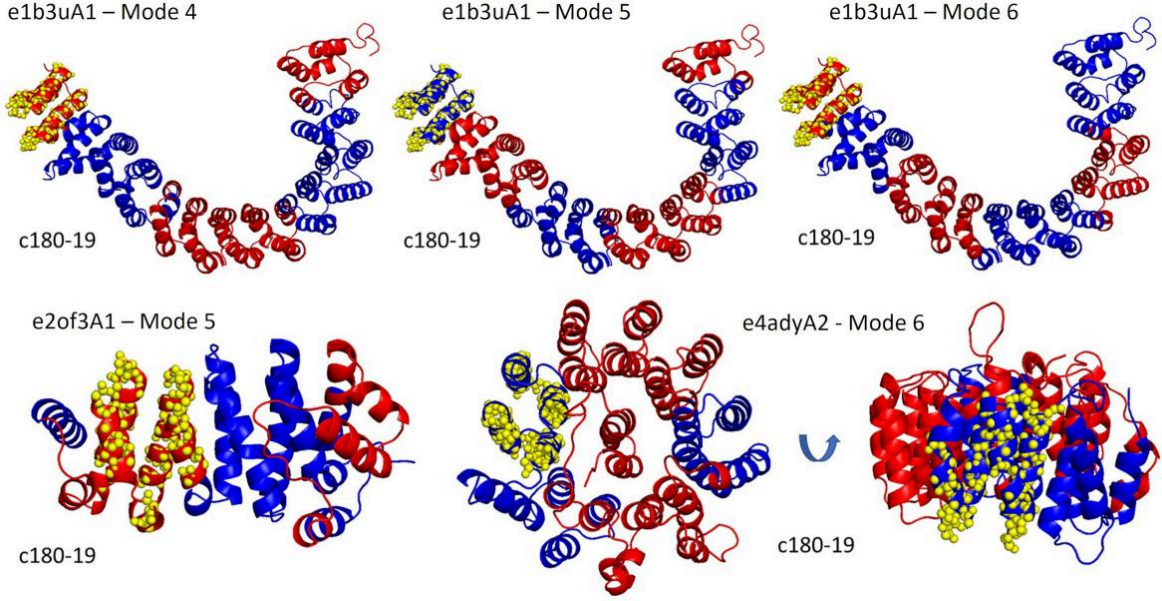
Theme c180-19 corresponds to DEs of the fourth, fifth, and sixth modes of e1b3uA1 (PP2A, PDB ID: 1B3U), and approximately to the fifth mode of e2of3A1 (ZYG-9, PDB ID: 2OF3) and sixth mode of e4adyA2 (26S proteasome subunit Rpn2, PDB ID: 4ADY). The marks and color codes are the same as in Fig. 6. Supplementary figs. S4 to S6, Supplementary Material online show projections of the modes and themes on the protein sequence.

We next examine the correlation between DEs and themes in repetitive alpha hairpin protein domains (ECOD X-group 109): Two homologs of the homology group (H-group) ARM-repeat (ECOD H-group 109.4): e2of3A1 from PP2A (PDB ID: 2OF3) and e1b3uA1 from ZYG-9 (PDB ID: 1B3 U) (with 50% sequence identity), and a domain from the H-group proteasome/cyclosome repeat (ECOD H-group 109.35) e4adyA2 from 26S proteasome subunit Rpn2 (PDB ID: 4ADY) (sequence identity of 27% and 26% to e2of3A1 and e1b3uA1, respectively) (Cheng et al. 2014). supplementary table S5, Supplementary Material online lists the themes detected in the three protein domains and their sequence positions, and supplementary figs. S4 to S6, Supplementary Material online compare these themes to the DEs of the seven slowest modes of each protein domain. Here, too, hinge points and themes’ edges often overlap, further demonstrating correlation between the DEs and the themes. For example, theme c180-36 approximately corresponds to DEs in mode-3 of e2of3A1 (supplementary fig. S4, Supplementary Material online), mode-4 of e1b3uA1 (supplementary fig. S5, Supplementary Material online), and mode-5 of e4adyA2 (supplementary fig. S6, Supplementary Material online) (Fig. 6). Similarly, theme c180-19 perfectly aligns with DEs of mode-5 in e2of3A1 (supplementary fig. S4, Supplementary Material online), with DEs of modes 4, 5, and 6 of e1b3uA1 (supplementary fig. S5, Supplementary Material online), and with mode-6 of e4adyA2 (supplementary fig. S6, Supplementary Material online), all of which form alpha-hairpins (Fig. 7).

#### α/β Architecture: TIM Barrels

The TIM barrel, one of the oldest folds, also made of repeats, is represented here with (domains from) three homologous proteins, namely, TatD-related deoxyribonuclease (PDB ID: 1J6O, ECOD Domain ID: e1j6oA1), Putative TatD-related DNAse (PDB ID: 2GZX, ECOD Domain ID: e2gzxA1), and Tat-linked quality control protein TatD (PDB ID: 4P5 U, ECOD Domain ID: e4p5uA1). The themes detected in these three protein domains and their sequence positions are listed in supplementary table S6, Supplementary Material online and are presented with the DEs of seven slow modes in supplementary figs. S7 to S9, Supplementary Material online. As in the cases of the propellers and alpha helix bundles, here, too, hinge points and themes’ edges often overlap, demonstrating the correlation between the themes and DEs.

**Fig. 8.**
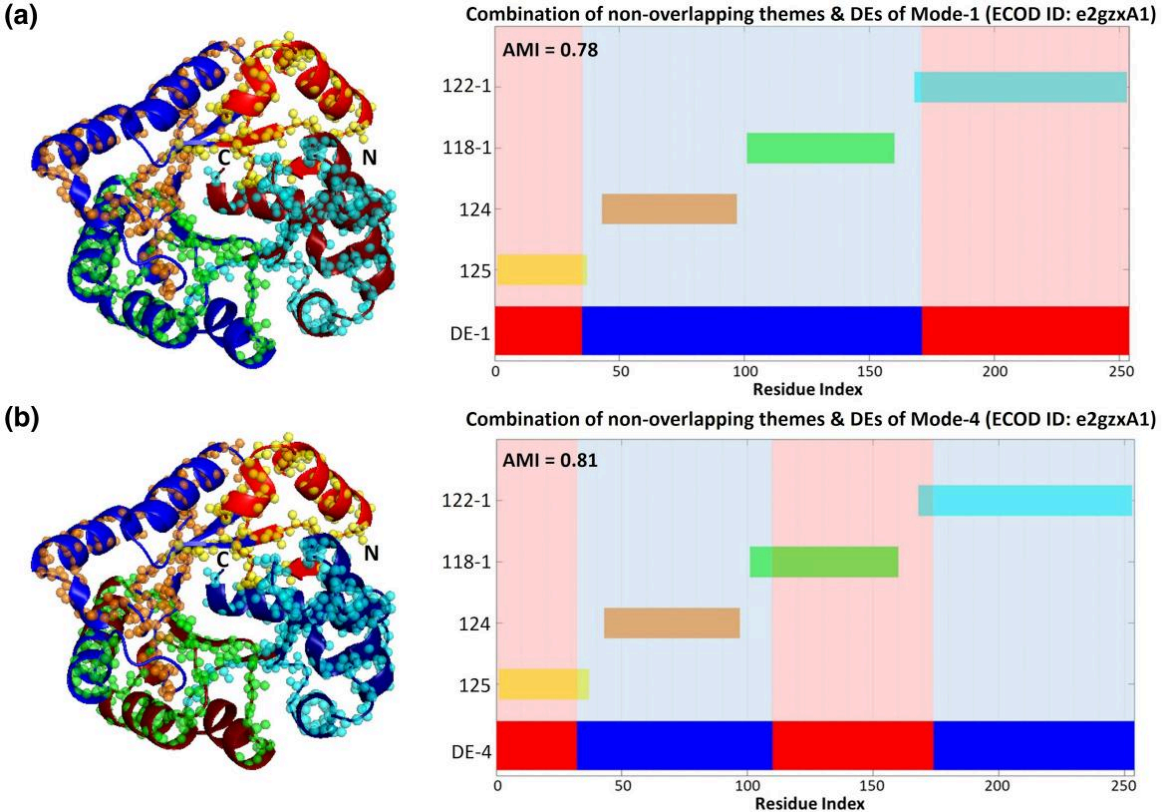
Correspondence between themes and functional dynamics in the e2gzxA1 domain (Putative TatD-related DNAse, PDB ID: 2GZX) TIM barrel. (Left) Themes projected on the 3D structure of e2gzxA1 together with the DEs of the first (a) and fourth (b) modes of motion. (Right) Projection of the same data on the protein sequence. DEs (shades of red and blue as in Fig. 3) and themes (various colors) are colored accordingly in both 3D and bar representations. In the fourth mode each of the four DEs roughly corresponds to a single theme, whereas in the first mode, the largest DE corresponds to a combination of themes 124 and 118-1.

As with the e2xyiA1 propeller, an anthology of nonoverlapping themes can collectively cover the entire sequence and correspond to all the DEs of specific modes. Figure 8 shows that the DEs of mode-1 and mode-4 of e2gzxA1 are entirely covered by the same four themes. However, in mode-4 there is one-to-one correspondence between the DEs and themes, while in mode-1 two themes (124 and 118) combine to cover a single DE.

**Fig. 9.**
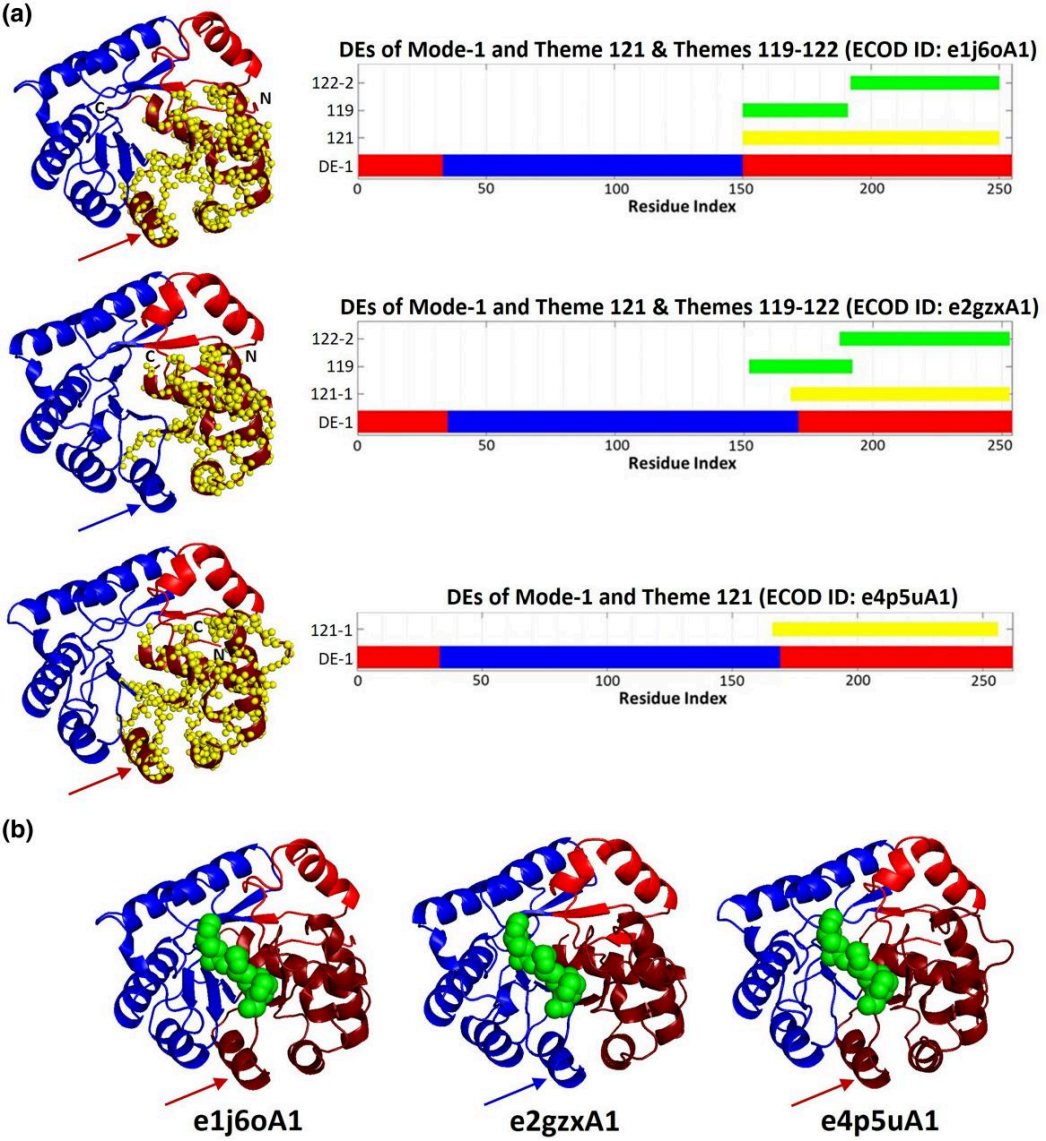
Themes and DEs in homologous TIM barrels. a) DEs of the slowest mode of motion of e1j6oA1 (TatD-related deoxyribonuclease, PDB ID: 1J6O), e2gzxA1 (Putative TatD-related DNAse, PDB ID: 2GZX), and e4p5uA1 (Tat-linked quality control protein TatD, PDB ID: 4P5U) are nearly identical, but the sense of the helix α6, marked with an arrow, is altered in e2gzxA1 compared to e1j6oA1 and e4p5uA1. Variations of theme 121, which corresponds to the C-terminal DE of this mode, capture this difference. A combination of themes 119 and 122 also corresponds to the same DE in e1j6oA1, but not in e2gzxA1 and e4p5uA1. All three structures are shown from similar views. Theme 121 is shown as yellow spheres on the 3D structures on the left, and the sequences’ positions are marked by the bars on the right. DEs are represented as shades of red and blue in 3D structures (as in Fig. 3). b) Projection of the trinucleotide DNA (from PDB ID: 4PE8) on e1j6oA1, e2gzxA1, and e4p5uA1 shows that the DNA is at the interface of the dynamic parts, which likely has a role in the trinucleotide DNA binding. The trinucleotide DNA is represented as green spheres.

Figure 9 compares the themes and DEs across the homologous TIM barrels. Variations of theme 121 cover a single DE from mode-1 in the C-terminus of each of the three homologous domains. It is noteworthy that the partitioning into DEs of e2gzxA1 is somewhat different from that of the other two homologs, where the DE in the C-terminus includes an additional α helix (α6, marked with an arrow in Fig. 9a), which alters its sense of correlations (red vs. blue) and becomes part of another DE in e1j6oA1 and e4p5uA1. Interestingly, this difference is also reflected in the variations of theme 121 vs. 121-2. Additionally, this DE of e1j6oA1 is also covered by a combination of two themes: 119 and 122. Theme 119 covers helix α6 (with the adjacent helix α7). Thus, theme 119 in combination with theme 122 does not align with this C-terminal DE in e2gzxA1, and it does not exist in e4p5uA1. On the other hand, the cooperative motion described by the slowest mode is likely the key mode to be associated with DNA binding (Fig. 9b). Here, the interface of the three DEs of the slow modes is probably adjusting the DNA binding mode. The DEs of e2gzxA1 display a difference compared with those of e1j6oA1 and e4p5uA1. The DNA segment is projected here from another homologous protein (PDB ID: 4PE8) to show the likely position of the trinucleotide DNA located at the interface of DEs in this most cooperative motion.

### In-depth Analysis of Non-repeat Domains

Next, we analyze the five non-repeat domains in our in-depth set; of these, two belong to ribosome-inactivating proteins (RIP), and three belong to Rossmann-related homology groups (Table 2). The themes detected in these domains and their sequence positions are listed in supplementary tables S7 and S8, Supplementary Material online. As in the repeat domains introduced above, here, too, hinge points often overlap with themes’ edges. Most of the DEs align with a single theme or with a combination of several themes, and/or themes align with either a single or several DEs (supplementary figs. S10 to S14, Supplementary Material online and Supplementary website).

**Fig. 10.**
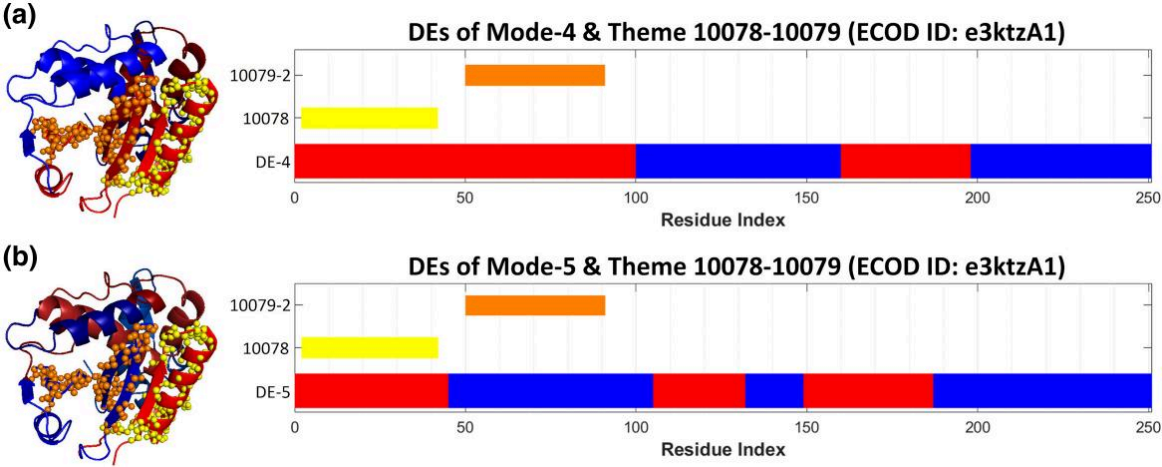
Correspondence between themes and dynamics in the e3ktzA1 domain of ribosome-inactivating protein gelonin (PDB ID: 3KTZ). a) A combination of themes 10078 and 10079 corresponds to a single DE of the fourth mode. b) Themes 10078 and 10079 individually correspond to two DEs of the fifth mode. Structures are shown from similar views. The themes are shown as yellow and orange spheres on the 3D structures on the left, and their sequences’ positions are marked by the bars on the right. The DEs are represented as shades of red and blue in the 3D structures, as in Fig. 3.

**Fig. 11.**
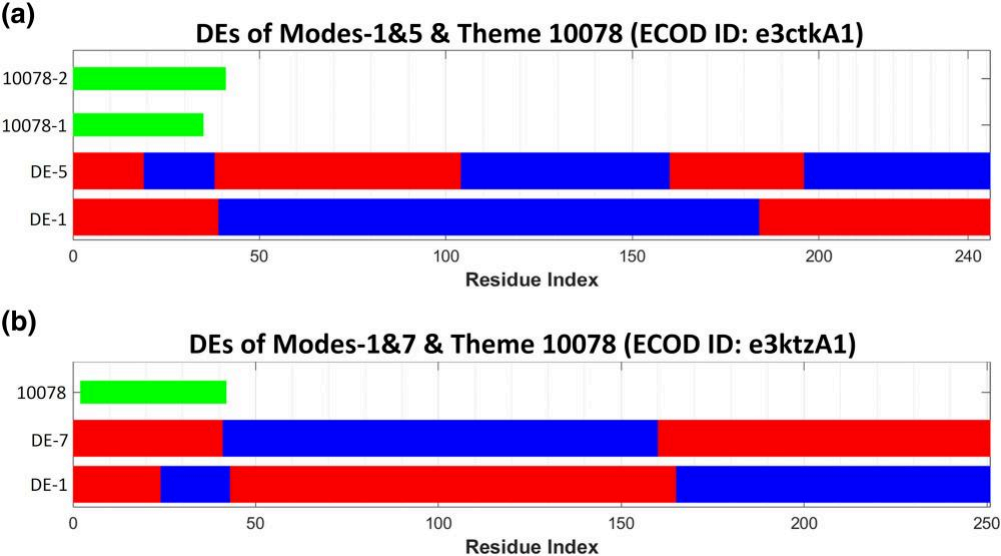
The same theme may correspond to a DE or a DE combination of different modes of motion in RIP homologues. a) Variations of theme 10078 correspond to a single DE of the first mode, and to a combination of two DEs of the fifth mode in e3ctkA1 (rRNA N-glycosidase, PDB ID: 3CTK). b) The same theme (marked in green) corresponds to a DE of the seventh mode and to a combination of two DEs of the first mode in e3ktzA1 (ribosome-inactivating protein gelonin, PDB ID: 3KTZ). DEs are shown in blue and red, corresponding to the opposite senses.

In the RIP homology group, for example, we observe that in domain e3ktzA1 (from ribosome-inactivating protein gelonin; PDB ID: 3KTZ), themes 10078 and 10079 correspond to different DEs of mode-5, whereas the same themes combined correspond to a single DE of mode-4 (Fig. 10). Additionally, in domain e3ctkA1 (from rRNA N-glycosidase; PDB ID: 3CTK), variations of theme 10078 correspond to a single DE of mode-1 (10078-1) and to a combination of two DEs of mode-5 (10078-2) (Fig. 11a). Likewise, in e3ktzA1, the same theme (10078) corresponds to a combination of two DEs in mode-1 as well as to a single DE of mode-7 (Fig. 11b). This example illustrates the flexibility in the alignment of DEs with variations of the same theme.

**Fig. 12.**
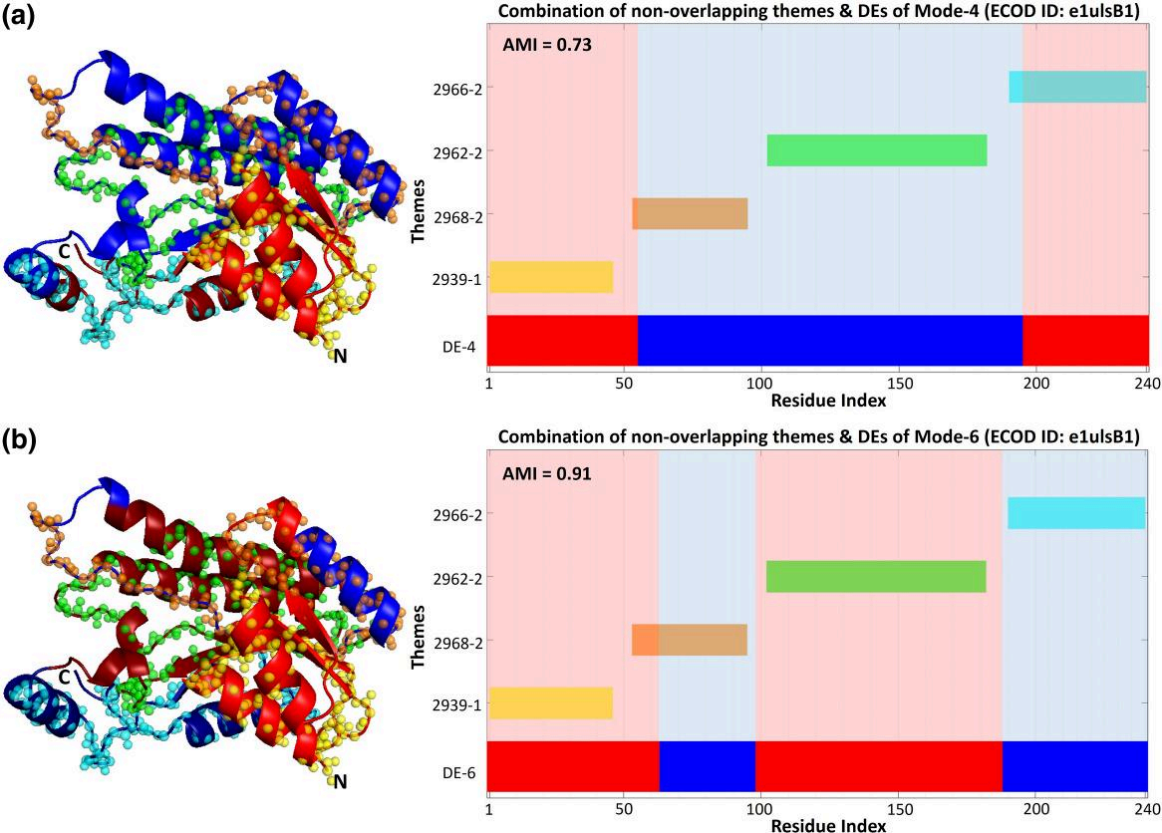
Correspondence between themes and functional dynamics in e1ulsB1 (putative 3-oxoacyl-acyl-carrier-protein reductase, PDB ID: 1ULS) Rossmann-related domain. (Left) Themes projected on the 3D structure together with the DEs of the fourth (a) and sixth (b) modes of motion. (Right) Projection of the same data on the protein sequence. DEs (shades of red and blue, as in Fig. 3) and themes (various colors) are colored accordingly in both 3D and bar representations.

For the Rossmann-related homology group, Fig. 12 shows that a selection of nonoverlapping themes can collectively cover the entire sequence and correspond to all the DEs from a specific mode, as shown above in the e2xyiA1 propeller and e2gzxA1 TIM barrel. DEs of mode-4 and mode-6 for putative 3-oxoacyl-acyl-carrier-protein reductase (PDB ID: 1ULS, ECOD Domain ID: e1ulsB1) are entirely covered by the same combination of nonoverlapping themes. As seen, a theme alone or in combination with other themes may correspond to a DE. For example, theme 2962 and theme 2968, in combination, align with a single DE (mode-4), yet theme 2962 may also align with a single DE individually (mode-6).

**Fig. 13.**
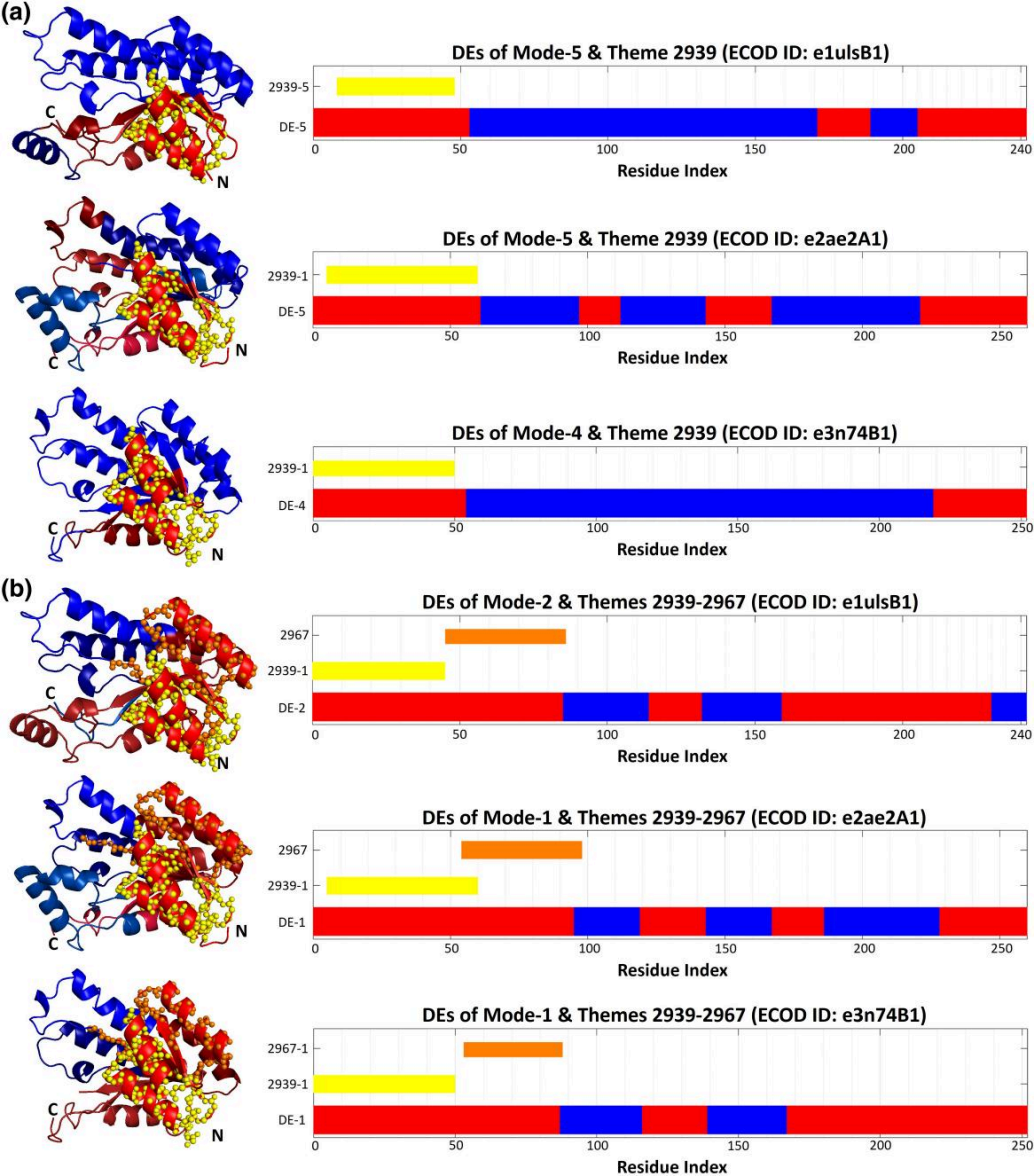
Correspondence between themes and DEs in Rossmann homologous domains. a) Variations of theme 2939 correspond to DEs in Rossmann-related domains e1ulsB1 (putative 3-oxoacyl-acyl-carrier-protein reductase, PDB ID: 1ULS) (mode-5), e2ae2A1 (tropinone reductase-II, PDB ID: 2AE2) (mode-5), and e3n74B1 (3-ketoacyl-(acyl-carrier-protein) reductase, PDB ID: 3N74) (mode-4). b) A concatenation of themes 2939 and 2967 corresponds to DEs in e1ulsB1 (mode-2), e2ae2A1 (mode-1) and e3n74B1 (mode-1). All three structures are shown from similar views. The themes are shown as yellow and orange spheres on the 3D structures on the left, and their sequences positions marked by the bars on the right. DEs are represented as shades of red and blue in 3D structures, as in Fig. 3.

In another example, for each of the three proteins with Rossmann-related homology, variations of theme 2939 correspond to a DE of mode-5 in e1ulsB1, to a DE of mode-5 for the e2ae2A1 domain in tropinone reductase-II (PDB ID: 2AE2), and a DE of mode-4 for the e3n74B1 domain in 3-ketoacyl-(acyl-carrier-protein) reductase (PDB ID: 3N74) (Fig. 13a). Variations of the same 2939 theme together with theme 2967 correspond to a DE of mode-2 in e1ulsB1, a DE of mode-1 in e2ae2A1, and a DE of mode-1 in e3n74B1 (Fig. 13b). As seen, variations of the same themes among homologs may compensate for variations in the dynamics between them, which further reinforces the correspondence between themes and DEs.

**Fig. 14.**
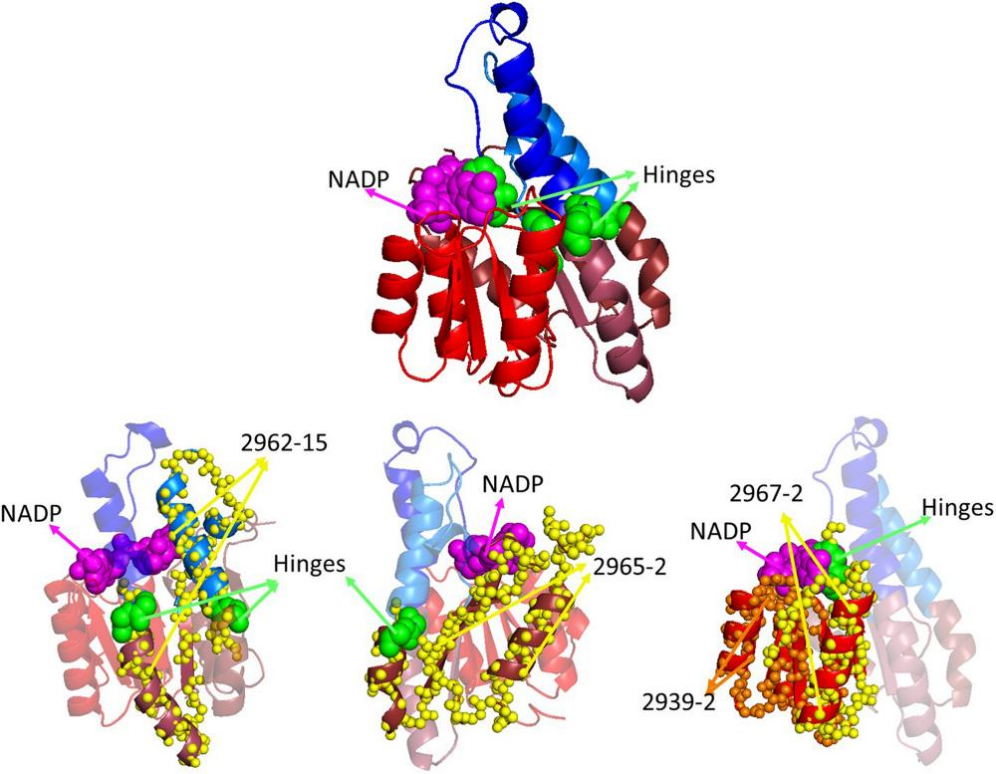
DEs (and themes) do not always correspond to secondary structure elements. DEs and hinges observed in the slowest mode of e3n74B1 (Rossmann-related 3-ketoacyl-(acyl-carrier-protein) reductase, PDB ID: 3N74) in complex with NADP (inferred from superimposition of the 3N74 structure with PDB ID: 3OP4). Themes that have termini near hinge points (2962-15, 2965-2, and 2967-2) are shown as yellow spheres with their related DEs in shades of red and blue, as in Fig. 3, and the remaining protein parts are shown using pale colors. NADP is shown as magenta spheres, and hinges are shown as green spheres.

DEs may correspond to secondary structure elements, with the hinges between them residing in loops that connect the elements. However, the Rossmann-related domain e3n74B1 (from the 3-ketoacyl-(acyl-carrier-protein) reductase, FabG) provides counter-examples. As seen in Fig. 14, the dynamic dissection of the slowest mode includes hinges in the middle of two long helices. Interestingly, the hinges correspond to the termini of themes 2962-15, 2965-2, and 2967-2. This illustrates that the sequence and dynamic dissection occur at a level different from what secondary structures may imply. The slowest mode may facilitate FabG binding to its NADP cofactor, which is key to the protein's function (Hou et al. 2016).

### In-depth set: Synthesis of Observations

Taking together our observations from our in-depth analysis, we observe that, in some cases, the same theme combination may correlate with more than one dynamic mode, i.e. the same sequence of themes complies with several inherent motions that are embedded in the structure. This occurs in the cases of the e2xyiA1 propeller, the e2gzxA1 TIM barrel, and the e1ulsB1 Rossmann-related domain (Figs. 3, 8, and 12). For example, themes 2939, 2968, 2962, and 2966 correspond to DEs of the fourth and sixth modes in e1ulsB1. On the other hand, between homologous domains, there may be a shift in mode space (e.g. the fifth dynamic mode of e1ulsB1 corresponds to the fourth dynamic mode of its homolog e3n74B1; Fig. 13). Additionally, comparison of homologous protein domains may reveal some alterations (differences in mode shape and DEs) across homologs’ corresponding dynamic modes. These alterations might be minor (e.g. when comparing the first dynamic modes of Rossmann-related homologs e1j6oA1, e2gzxA1, and e4p5uA1; Fig. 9a) or major (e.g. when comparing the first dynamic modes of RIP homologs 3CTK and 3KTZ; Fig. 11). Yet, these dynamic modes are still correlated with the themes and their combinations. Thus, the same themes (and combinations of themes) or their variations may align with the DEs in the corresponding modes between homologs as in Rossmann-related domains (Fig. 13). Moreover, new themes (and combinations of themes) may appear to align with more significant variations in slow modes as in TIM barrels (themes 119 and 122) (Fig. 9), adding viability to the correlation of the themes and DEs. Themes may appear in multiple positions along the protein domain (indexed as xxx-1, xxx-2, etc.). Unlike some themes in the propellers (Fig. 1, supplementary fig. S2, Supplementary Material online, Fig. 4), which are made of repeats, these positions are in similar segments of the protein in non-repeat domains (bar graph sections in supplementary figs. S10 to S14, Supplementary Material online). As a result, although specific themes cover relatively similar DEs among homologous protein domains in the corresponding modes and/or in different modes, their variations enable the theme to comply with the dynamics and thus function (Figs. 11 and 13).

### MI Analysis Quantifies the Statistical Significance of Correspondence Between Theme Boundaries and DEs

To quantify the apparent relationship between the DEs and the themes, we used MI analysis. Conventional MI does not provide any measure of statistical significance. Thus, we used two variants that innately consider the randomness factor by including the expected MI in the calculations, and provide some assessment of statistical significance (Romano et al. 2014): Adjusted mutual information (AMI), which ranges between 0 and 1 (1 being perfectly identical), and standardized mutual information (SMI), which measures the distance in SDs from dissimilarity (the larger the value the more similar the distributions). Complicating this analysis is the fact that DEs and themes are fundamentally different entities. In particular, the DEs of each mode always cover the whole structure, but themes typically do not. Also, DEs of a given mode do not overlap, while the themes sometimes do. To minimize gaps and overlaps, we sampled various combinations of themes, as described in the “Materials and Methods” below. A detailed description of our application of AMI and SMI to themes and DEs is provided in the “Materials and Methods” and supplementary text, Supplementary Material online.

To illustrate our analysis, we describe it in detail for the e2xyiA1 propeller. supplementary table S9, Supplementary Material online lists examples of 10 theme combinations out of 147 possible theme combinations of e2xyiA1 with (an arbitrarily chosen) overlap of three residues and gap restriction of eight residues. The AMI values calculated between each of the seven slowest modes of e2xyiA1 and all possible theme combinations range between 0.49 and 0.91, and the corresponding SMI values range between 79 and 160 (Table 3). MI results using other combinations of overlap and gap restrictions give AMI values of 0.48 to 0.94 and SMI values of 76 to 160 (supplementary tables S10 and S11, Supplementary Material online). These quantify the strong statistical correlation between the DEs and the themes, being particularly strong in the third-through-seventh modes. In the exemplary case of Fig. 3b, themes corresponding to the DEs of the seventh mode of the e2xyiA1 propeller are assigned a high AMI score of 0.91. For comparison, supplementary fig. S15, Supplementary Material online typifies a case with low similarity (AMI score of 0.47).

**Table 3:** AMI and SMI values for the correlation between the DEs of each of the seven slowest GNM modes of e2xyiA1 and theme combinations, filtered with thresholds of a 3-residue overlap and an 8-residue gap

AMI	DEs Mode-1	DEs Mode-2	DEs Mode-3	DEs Mode-4	DEs Mode-5	DEs Mode-6	DEs Mode-7
**Minimum**	0.49	0.52	0.61	0.54	0.65	0.58	0.72
**Maximum**	0.67	0.64	0.73	0.71	0.80	0.71	0.91
**Average**	0.55	0.57	0.66	0.63	0.73	0.64	0.82

SMI	DEs Mode-1	DEs Mode-2	DEs Mode-3	DEs Mode-4	DEs Mode-5	DEs Mode-6	DEs Mode-7
**Minimum**	86	92	98	79	87	94	123
**Maximum**	127	128	133	109	111	124	160
**Average**	99	106	112	94	99	108	137

We also applied MI analysis to all domains examined in detail above (the in-depth set). For this set, we used thresholds of a 3-residue overlap and an 8-residue gap. We note that the MI analysis excluded the alpha helix bundle domains studied in detail above (e2of3A1, e1b3uA1, and e4adyA2) because themes only partially cover each of these protein domains, preventing a meaningful calculation of the correlation with the DEs. MI analysis results for the in-depth set present equally significant correlations.

Table 4 lists the mean AMI and SMI values of the MI analysis for the domains in the in-depth set, together with the maximum AMI and SMI values observed in each domain. The averages of the mean AMI and SMI values over the 10 domains are 0.64 and 90, respectively, with corresponding SDs of 0.09 and 14. When the maximum AMI and SMI values for each domain are considered, the analysis gives 0.85 as the average of maximum AMI values and 133 as the average of the maximum SMI values (Table 4). The correlations at the resolution of individual modes are listed in Table 5 (calculated mean AMI values) and supplementary table S12, Supplementary Material online (corresponding SMIs). Table 6 lists the calculated maximum AMI values, and supplementary table S13, Supplementary Material online the corresponding SMI values. DEs of the vast majority of the slow modes show significant correlation with the themes. Overall, our results for representatives of different protein families strongly support the correlation between the DEs and the themes. Furthermore, we observe that DEs and themes show a high correlation (AMI > 0.70) in multiple modes of motion for all cases, suggesting that themes contribute to protein dynamics in multiple wirings.

**Table 4:** MI analysis of the single domains in the in-depth set

Domain ID	Mean AMI	SD (AMI)	Max AMI	Mean SMI	SD (SMI)	Max SMI
**e2xyiA1**	0.66	0.09	0.91	108	15	160
**e3emhA1**	0.59	0.12	0.92	99	20	178
**e1j6oA1**	0.64	0.05	0.74	90	12	113
**e2gzxA1**	0.66	0.07	0.81	89	12	120
**e4p5uA1**	0.66	0.09	0.81	94	18	140
**e3ktzA1**	0.68	0.09	0.89	87	10	114
**e3ctkA1**	0.63	0.08	0.84	83	12	126
**e1ulsB1**	0.64	0.10	0.91	78	16	126
**e2ae2A1**	0.64	0.08	0.80	85	17	130
**e3n74B1**	0.58	0.09	0.84	86	11	119
**AVERAGE**	0.64	0.09	0.85	90	14	133

The ECOD IDs of the domains are listed on the left column, and the next columns provide the calculated mean values, standard deviations (SDs), and maximum values of AMI and SMI.

**Table 5:** The mean AMI values for individual modes of each of the domains in the in-depth set

Domain ID	DEs Mode-1	DEs Mode-2	DEs Mode-3	DEs Mode-4	DEs Mode-5	DEs Mode-6	DEs Mode-7
**e2xyiA1**	0.55	0.57	**0.66**	**0.63**	**0.73**	**0.64**	**0.82**
**e3emhA1**	0.57	0.59	N/A	0.55	**0.76**	**0.68**	*0.41*
**e1j6oA1**	**0.68**	0.54	**0.64**	**0.67**	**0.64**	**0.70**	**0.62**
**e2gzxA1**	**0.62**	0.56	**0.68**	**0.71**	**0.67**	**0.67**	**0.71**
**e4p5uA1**	**0.78**	0.51	**0.64**	**0.66**	**0.69**	**0.66**	**0.70**
**e3ktzA1**	**0.64**	**0.61**	**0.75**	**0.66**	**0.83**	**0.62**	**0.68**
**e3ctkA1**	**0.66**	0.58	**0.70**	**0.64**	**0.68**	0.51	**0.64**
**e1ulsB1**	*0.44*	**0.64**	**0.62**	**0.63**	**0.66**	**0.74**	**0.70**
**e2ae2A1**	**0.65**	**0.69**	0.59	0.51	**0.75**	**0.68**	**0.64**
**e3n74B1**	**0.63**	0.51	0.56	**0.63**	0.51	**0.71**	0.54

Italic, AMI < 0.5; Bold, AMI > 0.6.

**Table 6:** The maximum AMI values for individual modes of each of the domains in the in-depth set

Domain ID	DEs Mode-1	DEs Mode-2	DEs Mode-3	DEs Mode-4	DEs Mode-5	DEs Mode-6	DEs Mode-7
**e2xyiA1**	0.67	0.64	**0.73**	**0.71**	**0.80**	**0.71**	**0.91**
**e3emhA1**	**0.73**	0.67	N/A	0.66	**0.92**	**0.83**	*0.55*
**e1j6oA1**	**0.72**	*0.56*	0.65	0.68	0.64	**0.74**	0.62
**e2gzxA1**	**0.78**	0.64	**0.75**	**0.81**	**0.77**	**0.70**	**0.80**
**e4p5uA1**	**0.81**	*0.57*	**0.72**	**0.71**	**0.78**	0.69	**0.74**
**e3ktzA1**	**0.73**	0.69	**0.79**	**0.73**	**0.89**	0.65	**0.76**
**e3ctkA1**	**0.81**	0.67	**0.84**	**0.73**	**0.76**	0.66	**0.75**
**e1ulsB1**	*0.51*	0.69	0.68	**0.80**	**0.76**	**0.91**	**0.79**
**e2ae2A1**	0.69	**0.79**	0.69	*0.56*	**0.80**	**0.77**	0.66
**e3n74B1**	**0.75**	0.61	**0.70**	**0.76**	0.61	**0.84**	0.69

Italic, AMI < 0.6; Bold, AMI > 0.7.

Lastly, we performed MI analysis on the expanded set of 150 domains to further support our findings. The maximum AMI and SMI values are presented for each domain in supplementary file S1, Supplementary Material online, and detailed results for each domain are available in the Supplementary website (https://gabiaxel.github.io/themes-dynamics/). In the expanded set, we observed a high correlation (AMI > 0.70) for an average of 3.9 modes in the 150 domains examined. For 131 of the 150 domains, high AMI values were observed for two or more modes of motion.

### *P*-value Analysis for the Statistical Significance of Correspondence Between Themes and DEs

To further consolidate and reinforce our findings, we used conventional MI analysis to assign *P*-values to the correlations between themes and DEs. This analysis provides an additional independent measure of significance beyond the AMI and SMI values, which inherently consider the expected MI.

We generated random sets of continuous protein segments resembling the themes identified in each domain. We calculated the MI between random set combinations and the DEs, and between the real theme combinations and the DEs in each individual mode. Then, we conducted *P*-value calculations based on the distribution of the MI of the random set combinations and the DEs. Probability distribution of MI for the randomly generated themes of the e2xyiA1 propeller and DEs of mode-5 is provided in supplementary fig. S16, Supplementary Material online, with the MI and *P*-value obtained for the real themes marked on the distribution graph. Details regarding the random generation process and *P*-value calculations are provided in the “Materials and Methods” section.

**Fig. 15.**
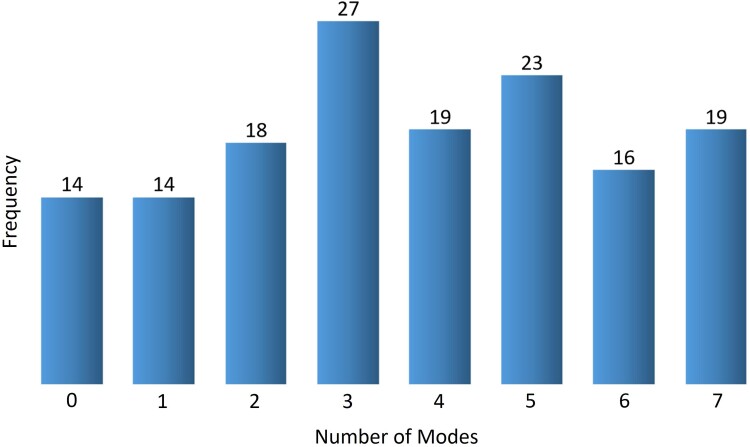
The distribution of statistical significance per domain. The *x* axis is the number of modes with statistically significant (*P*-value < 0.05) correlations with themes in each domain, and the *y* axis is the domain frequency. Total number of domains in the set: 150. Average number of statistically significant modes per domain: 3.7 of a total of 7.

Table 7 lists the *P*-values assigned to the calculated MI between the themes and DEs for each of the seven slowest modes of motion in each of the 10 domains considered from the in-depth set. Significant *P*-values (< 0.05) were assigned to multiple individual modes of motion in each of the domains. Figure 15 shows a histogram of the number of modes with significant *P*-values (< 0.05) per domain in the expanded set of 150 domains. The distribution averages at 3.8 modes of motion with a SD of 2.1 modes. We observed at least one mode with significant *P*-value (< 0.05) for 136 domains out of 150, and multiple modes with significant *P*-value (< 0.05) for 122 domains out of 150. *P*-values are presented for each of the 150 domains in supplementary file S2, Supplementary Material online, and detailed results for each domain are available in the Supplementary website (https://gabiaxel.github.io/themes-dynamics/). It is important to note that domains lacking modes associated with a significant *P*-value typically manifest fewer themes and smaller domain sizes compared to their counterparts. Overall, the *P*-value analysis shows that the correspondences between themes and DEs in multiple levels shown by MI are statistically significant.

**Table 7:** *P*-values for the correlation between the themes and DEs in each individual mode for each domain in the in-depth set

Domain ID	DEs Mode-1	DEs Mode-2	DEs Mode-3	DEs Mode-4	DEs Mode-5	DEs Mode-6	DEs Mode-7	# modes *P* < 0.05
**e2xyiA1**	**0.0165**	**0.0224**	**0.0133**	**0.0022**	**0.0004**	**0.0132**	**0.0001**	7
**e3emhA1**	**0.033**	0.1131	N/A	0.0865	**0.0013**	0.0913	0.1929	2
**e1j6oA1**	**0.0143**	0.2355	**0.0116**	**0.0186**	**0.0165**	**0.0046**	**0.0437**	6
**e2gzxA1**	**0.004**	0.1107	**0.0074**	**0.0049**	**0.0013**	**0.0217**	**0.0023**	6
**e4p5uA1**	**0.0015**	0.1588	0.089	**0.0133**	**0.0013**	0.068	**0.0052**	4
**e3ktzA1**	**0.0187**	**0.0354**	**0.0081**	**0.0247**	**0.0004**	0.1228	**0.0177**	6
**e3ctkA1**	**0.0089**	0.0719	**0.0199**	**0.0288**	**0.0108**	0.185	0.0756	4
**e1ulsB1**	0.1621	0.0669	0.0736	**0.0102**	**0.0227**	**0.0073**	**0.0437**	4
**e2ae2A1**	**0.0113**	**0.0102**	**0.0376**	0.1308	**0.0012**	**0.0075**	**0.0291**	6
**e3n74B1**	0.1936	0.1212	**0.0388**	**0.0212**	0.1212	**0.0063**	0.1111	3

Significant *P*-values (< 0.05) are shown as bold. The dynamic segments of Mode-3 in domain e3emhA1 are too short to yield DEs, and obviously cannot be assigned a *P*-value; they are marked as N/A.

## Conclusion

Our detailed analysis of the relationship between the themes that are found in 13 protein domains and the DEs of their slowest modes reveals a nontrivial observation: The boundaries of DEs often coincide with those of themes. Themes and DEs are two types of sub-domain sized segments that were determined via two independent computational procedures. Like Pfam entries, the themes were found using sequence similarity search and manifest reuse in protein space (Nepomnyachiy et al. 2017). The DEs, on the other hand, were derived from GNM calculation and reflect functional dynamics. In both cases, the segments are not very short. DEs contain at least 15 residues and themes at least 35 residues, implying that only relatively few positions along the protein chain are among their boundaries. Thus, the (statistically significant) boundary overlaps between these two different entities, i.e. the DEs and themes, cannot be trivially explained.

It is helpful to consider the dynamic behavior of multidomain proteins as a reference, as domains, like themes, are protein segments reused in evolution. Indeed, multiple studies have shown that structural domains correspond to the moving parts within specific modes of motion (Granata et al. 2017; Zhang et al. 2020), and that these motions are important for protein function. For example, several slow modes of motion underlie the cooperative movements of the two transmembrane domains and two NBDs for unidirectional transport of the substrate in ABC transporters (Acar et al. 2020). The mechanistic role of domain interfaces in this case is embedded in intrinsic dynamics and allosteric pathways (Liang et al. 2018; Zhang et al. 2020). In the allosteric signaling of human estrogen receptor alpha, the interface of the DNA- and ligand-binding domains also has a functional role (Huang et al. 2018). Likewise, in metabotropic glutamate receptors (mGluRs), structural changes at the dimer interface are coupled with the receptor activation mechanism (Xue et al. 2014). Finally, in eukaryotic cytoplasmic Hsp70s, the coupling of interface dynamics and the global functional motion (i.e. slow modes of motion), provides a mechanistic framework that could explain how the modulation of the interfaces may lead to functional changes and perhaps functional adaptation in evolutionary diversification (Meng et al. 2018).

Unlike domains, which are assumed to fold autonomously, themes may be too short to fold independently. The abundance of their reuse throughout protein space, and in particular within ancient protein families (Longo et al. 2020; Kolodny et al. 2021; Qiu et al. 2022), suggests that these reused segments are important (Shakhnovich et al. 1996; Holm and Sander 1999; Chothia et al. 2003; Socolich et al. 2005, Lee et al. 2007; Liberles et al. 2012; Nepomnyachiy et al. 2014, 2017; Alva et al. 2015; Edwards and Deane 2015; Alva and Lupas 2018; Ben-Tal and Lupas 2021). In many instances, their importance is due to a specific function such as binding, as in the case of the beta-propeller in Fig. 5. Following the hypothesis that domains have emerged from smaller building blocks (Eck and Dayhoff 1966; Lupas et al. 2001), we suggested that they formed from mixing-and-matching of themes (Nepomnyachiy et al. 2017; Kolodny et al. 2021). However, as discussed above, for a theme combination to persist through evolution, the themes must fit together—not only geometrically but also dynamically. Indeed, without the resulting functional dynamics, the domain would not be able, for example, to shift between active and inactive conformations and to allosterically control the transition (Modi et al. 2021). Our findings herein are compatible with the premise of mixing-and-matching, showing that, in many cases, evolutionarily reused parts move together within certain modes of motion—thereby facilitating internal protein transduction, which is key to biological function. Indeed, dynamics is crucial for the function of each of the protein domains we analyzed in detail. Thus, the correlation between the themes and DEs may be viewed as another evolutionary relic, offering further support to the hypothesis that themes play important roles in protein evolution.

Though our MI analyses offer convincing evidence of the correspondence between themes and DEs, these results should be interpreted with caution. In particular, our MI calculation does not account for dependencies between the amino acid positions within a protein domain, e.g. due to steric hindrance or secondary structure (Finkelstein and Ptitsyn 1987; Murzin 1998; Orengo et al. 2001; Skolnick et al. 2014). Such correlations may have an impact on the accuracy of our approximations. More importantly, the existence of a correlation between themes and DEs does not necessarily imply causality. It is tempting to suggest, as we do here, that the correlation results from evolutionary preference for proteins capable of dynamic transduction, leading to a selection of themes that dynamically match with each other in protein structure. However, we cannot disprove that the correlation is due to yet-to-be discovered confounding factors. Regardless, this correlation is interesting and has many implications. One practical implication is for grafting protein parts in protein engineering (Eisenbeis et al. 2012; Höcker 2014; Jacobs et al. 2016). Examination of the themes and/or DEs that compose a protein may readily suggest parts to be grafted. Clever design based on these building blocks may guarantee both geometrical and dynamic match between the grafted parts, mimicking evolutionary processes. Perhaps even more significantly, our findings suggest that models for the emergence of protein domains in evolution can and should account for dynamics—thus capturing a property that is key to protein function.

## Materials and Methods

### Datasets

Two datasets were compiled specifically for this work: an in-depth set and an expanded set (which encompasses the in-depth set). The in-depth set consists of eight repeat and five non-repeat ECOD domains used for detailed inspection. The expanded set consists of 150 ECOD domains belonging to 26 different ECOD H-groups used for systematic statistical analysis.

#### In-depth set

The set includes both repeat and non-repeat domains to cover maximal structural diversity. The repeat domains include eight large symmetrical protein domains from the database of reuse in proteins (Nepomnyachiy et al. 2017). These domains, presented in Table 1, include obvious examples of repeated elements that result in symmetrical geometry: Two homologous seven-blade beta-propellers—histone-binding protein CAF1 (PDB ID: 2XYI, ECOD Domain ID: e2xyiA1) and WD repeat-containing protein 5 (WDR5, PDB ID: 3EMH, ECOD Domain ID: e3emhA1); two homologous ARM-repeats—ZYG-9 (PDB ID: 2OF3, ECOD Domain ID: e2of3A1) and protein phosphatase PP2A (PDB ID: 1B3U, ECOD Domain ID: e1b3uA1); a repetitive alpha hairpin—26S proteasome subunit Rpn2 (PDB ID: 4ADY, ECOD Domain ID: e4adyA2); and three homologous TIM barrels—TatD-related deoxyribonuclease (PDB ID: 1J6O, ECOD Domain ID: e1j6oA1), Putative TatD-related DNAse (PDB ID: 2GZX, ECOD Domain ID: e2gzxA1), and Tat-linked quality control protein TatD (PDB ID: 4P5U, ECOD Domain ID: e4p5uA1). This sample includes protein domains of three very different architectures: all-beta, all-alpha, and alpha/beta. Our sample of non-repeat domains includes five domains from two different ECOD H-groups. These domains, presented in Table 2, include two homologous RIP domains—ribosome-inactivating protein gelonin (PDB ID: 3KTZ, ECOD Domain ID: e3ktzA1) and rRNA N-glycosidase (PDB ID: 3CTK, ECOD Domain ID: e3ctkA1); and three homologous Rossmann-related domains—putative 3-oxoacyl-acyl-carrier-protein reductase (PDB ID: 1ULS, ECOD Domain ID: e1ulsB1), tropinone reductase-II (PDB ID: 2AE2, ECOD Domain ID: e2ae2A1), and 3-ketoacyl-(acyl-carrier-protein) reductase (PDB ID: 3N74, ECOD Domain ID: e3n74B1).

#### Expanded Set

We systematically generated a significantly larger collection of domains characterized by enhanced structural diversity to facilitate a comprehensive statistical analysis. A paramount criterion guiding the selection of this set was the maximal coverage of domain sequences by themes, thereby enabling a meaningful investigation of their correlation with the dynamic components. To achieve this objective, we conducted a systematic scan of the database of reuse in proteins (Nepomnyachiy et al. 2017), and domains conforming to the specified criterion were identified. Among the domains meeting this criterion, elements with very high structural similarity from those in the same homology group (RMSD < 1.25 Å) were removed from the set to eliminate redundancy. Finally, if the dynamic segments within multiple modes of a given domain were too short to allow decomposition into DEs with our threshold of 15 amino acids, that domain was removed from the set, to ensure robustness of the statistical analysis. We ended up with 150 ECOD domains belonging to 26 different ECOD H-groups. Detailed results for each domain in this set are provided in the Supplementary website as session files and spreadsheets (https://gabiaxel.github.io/themes-dynamics/).

### Themes

The themes, detected based on sequence similarity alone, are protein segments shared between different proteins (Nepomnyachiy et al. 2017). The themes of the domains in the in-depth set are listed in supplementary tables S3 to S8, Supplementary Material online (http://trachel-srv.cs.haifa.ac.il/rachel/ppi/themes; https://gabiaxel.github.io/themes-dynamics/). As an example, the 36 themes detected in the histone-binding protein CAF1 (2XYI) of the propeller fold are presented in Fig. 1; their lengths vary from 35 to 101 amino acids. Variations are found for some of the themes in multiple positions along the protein; an additional index is added to these (e.g. 14815-1 through 14815-4).

The themes of the domains in the expanded set can be observed in https://trachel-srv.cs.haifa.ac.il/rachel/ppi/themes & https://gabiaxel.github.io/themes-dynamics/.

### Dynamic Segments and DEs

To examine the structural dynamics, we used GNM calculations (Bahar et al. 1997; Haliloglu et al. 1997). GNM decomposes residue fluctuations of a given protein structure into a set of orthogonal modes of motion. These span the whole range from the most collective global motions through local fluctuations.

According to the model, the equilibrium correlation between the fluctuations of two residues *i* and *j*, respectively, Δ**R***_i_* and Δ**R***_j_*, is given as:


(1) $$\left\langle \Delta R_{i}\cdot \Delta R_{j}\right\rangle =\left(\frac{3k_{b}T}{\gamma }\right)[\Gamma^{-1}{]}_{ij},$$

where **Γ** is a symmetric matrix known as a Kirchhoff (connectivity) matrix. *γ* is the force constant of the Hookean pairwise potential function, which represents the interactions between the residues in the folded structure. *T* is the absolute temperature in Kelvin degrees, and *k*_b_ is the Boltzmann constant.

The elements of **Γ** are given by


(2) $$\Gamma_{ij}=\{\begin{array}{c} -1\;\;\mathrm{if}\;i\neq j\;\mathrm{and}\;R_{ij}\;\leq \;r_{c},\\ 0\;\mathrm{if}\;i\neq j\;\mathrm{and}\;R_{ij}\;>\;r_{c},\\ -\underset{i,i\neq j}{\sum }\;\Gamma_{ij}\;if\;i=j\;\;. \end{array}$$

where *R_ij_* is the distance between the *C_α_* atoms of *i*-th and *j*-th amino acids, and the *r_c_* threshold defines whether they are close enough to interact. *r_c_* = 10 Å is used here.

Equation (1) can be rewritten as:


(3) $$\left\langle \Delta R_{i}\cdot \Delta R_{j}\right\rangle =\left(\frac{3k_{b}T}{\gamma }\right)[U(\Lambda^{-1})U^{T}{]}_{ij}=\left(\frac{3k_{b}T}{\gamma }\right)\overset{n-1}{\underset{k=1}{\sum }}\;[\lambda_{k}^{-1}u_{k}u_{k}^{T}{]}_{ij},$$

where *k* is the *k*-th vibrational mode in the spectrum of *n* − 1 modes, *n* being the number of residues. **U** is an orthogonal matrix whose columns **u***_i_* are the eigenvectors of **Γ**, and **Λ** is the diagonal matrix of the eigenvalues *λ_k_*. For each individual mode *k*, the normalized residue correlations between residue pairs can be written as


(4) $$[\Delta R_{i}\cdot \Delta R_{j}{]}_{k}=\;\frac{3k_{b}T}{\gamma }\;\;\lambda_{k}^{-1}\;[u_{k}{]}_{i}[u_{k}{]}_{j},$$

where [*u_k_*]*_i_* and [*u_k_*]*_j_* are unit vectors.

This results in a symmetric matrix of residue correlations composed of “+ 1” and “− 1” for each mode. Thus, in each individual mode, the equilibrium correlations between residue pairs display if they fluctuate in the same sense, i.e. are positively correlated (+ 1), or opposite sense, i.e. negatively correlated (− 1) (Emekli et al. 2008). Any row or column of this matrix decomposes the structure in two, based on the sense of correlations; thus, each mode includes two dynamic parts (red and blue) that move in opposite senses around hinges. A toy model to represent decomposition of the structure into the dynamic parts from a correlation matrix of GNM is demonstrated in supplementary fig. S17, Supplementary Material online.

**Fig. 16.**
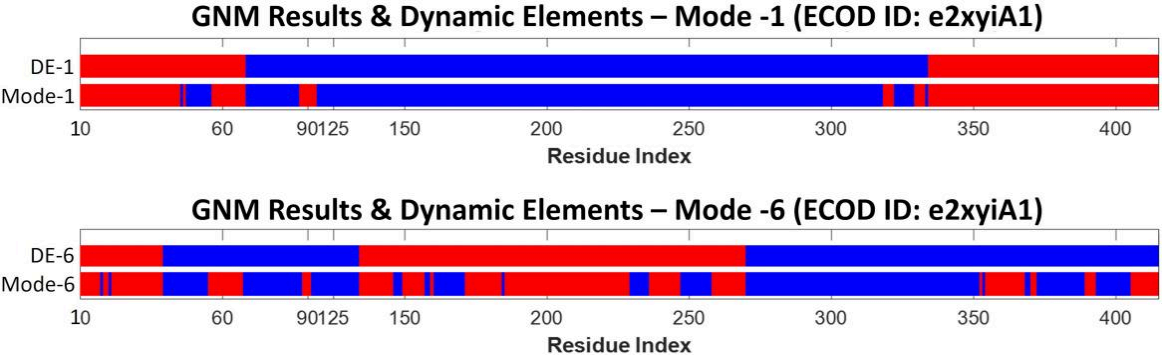
Dynamic segments and DEs in the e2xyiA1 propeller (from the CAF1 protein, PDB ID: 2XYI). For representative purposes only two of the slow modes of motion are shown: The first and sixth. The dynamic segments of each mode are marked in blue and red along the protein sequence (Mode-1 and Mode-6). The DEs (DE-1 and DE-6) are filtered versions of the dynamic segments, after smoothing using a window of 15 amino acids. Each of the red and blue segments (after smoothing) is considered a DE.

The dynamic parts can then be projected onto the amino acid sequence of the protein, where a continuous stretch of amino acids of the same dynamic part is called a “dynamic segment” (see, e.g. the blue and red stripes in Fig. 16). To avoid minor fluctuations, short fragments containing less than 15 residues are merged with the neighboring longer dynamic segment. The merged parts, the upper bars in the example of Fig. 16, are called DEs. Thus, the DEs are defined based on dynamics analysis only.

The modes are sorted according to their eigenvalues from 1 to *n* ‒ 1, such that the first modes, also called the “slowest modes”, underlie the largest and most cooperative global motions. The distribution of the relative contributions of the dynamic modes shows that the overall motion of the protein is dominated by the slowest modes (supplementary fig. S18, Supplementary Material online). We therefore restricted the analysis to the seven slowest modes. This (arbitrary) threshold was selected because the contribution of the next modes to the overall dynamics decreases. Additionally, beyond the seventh mode, the number of fragments smaller than 15 residues, which combine with longer dynamic segments as explained in the previous paragraph, significantly increases (supplementary fig. S19, Supplementary Material online).

### Comparison of DEs With GNM and MD Simulations

GNM, in spite of its simplicity and use of approximated linear dynamics, can reveal long-time behavior (Micheletti et al. 2004; Wang et al. 2004; Yang et al. 2007; Bahar et al. 2010; Togashi and Flechsig 2018) and allow us to observe protein dynamics on multiple levels, in various slow modes of motion; we utilize these properties in the present work. Nevertheless, to illustrate the comparison of the dynamic dissection into simpler components by GNM and MD simulations, we analyzed type-2 angiotensin 2 receptor (PDB ID: 5UNH). MD trajectory, topology, and model files were obtained from the GPCRmd dataset (Rodríguez-Espigares et al. 2020). The accumulated simulation time of the MD simulation is 1.5 µs. We calculated the correlations between residue fluctuations and performed principal component analysis on the covariance matrix to obtain eigenvalues and eigenvectors. We compared the seven slowest GNM modes individually with the MD principal components (PCs) that capture the largest variations. Each PC is binarized to 1 for positive and − 1 for negative correlations between residues. We observe one-to-one correspondence between GNM modes and PCs (supplementary table S14, Supplementary Material online, supplementary figs. S20 to S26, Supplementary Material online), and the DEs obtained with GNM and MD are observed to be very similar (supplementary fig. S27, Supplementary Material online). To note, the slowest and second slowest GNM modes correspond to the same PC (PC3), and PC6 does not correspond to any of the slow modes. The variations (eigenvalues) captured by each PC may change with the length of the MD simulation. However, the essential motions represented by the corresponding PCs seem to be well accounted for with slow modes of GNM.

We also compare here the DEs with themes of GPCRs (ECOD X-group 5001) via the HHsearch engine. Since the conservation levels among GPCRs are high, we obtain relatively long themes compared to the ones in our in-depth set. Yet, we observe that the correspondence between themes and DEs (obtained with both GNM and MD simulations) still holds. Theme-1 corresponds to two DEs of the third slowest mode of GNM and PC1. Furthermore, Theme-2 and Theme-3 correspond to a single DE (first and second DEs) of the same GNM mode and the same PC (supplementary fig. S28, Supplementary Material online).

### AMI and SMI

MI is a commonly used measure for comparing clusters. Consider two random variables *x* and *y* with a joint probability mass function *p*(*x*, *y*) and marginal probability mass functions *p*(*x*) and *p*(*y*). MI (*x*; *y*) is the difference in relative entropy between the joint distribution *p*(*x*, *y*) and the product distribution *p*(*x*) *p*(*y*) (Cover and Thomas 1991).

Here we use two related measures: AMI and SMI.

AMI is the normalized variant of MI. AMI ranges between 1, when the two partitions are identical, and 0, when the MI between two partitions equals to the value expected by chance alone (Vinh et al. 2010).

SMI is obtained by probabilistic adjustment for chance on MI; it is simply the standardized form of MI. The SMI value is the number of SDs of the MI from the mean, under a null distribution of random clustering solutions with fixed marginal (Romano et al. 2014). Thus, an SMI value of 50 signifies that the MI is 50 SDs away from the mean of random clustering solutions.

Consider two types of clusterings from a dataset consisting of N records: D (DEs) and T (themes). Let the data in D be clustered in *k* clusters (number of DEs obtained in a specific mode) of size *d_i_* for each cluster *i* = 1, …, *k*, and let the data in T be clustered in *l* clusters (number of themes in a specific combination) of size *t_j_* for each cluster *j* = 1, …, *l*. The number of records shared between clusters *i* and *j* is expressed as *n_ij_*. The overlap between the two clusterings can be represented in a matrix form by the *k* × l contingency supplementary table S15, Supplementary Material online.

The equations used in the calculation of MI, AMI and SMI between two clusterings are given in the supplementary text, Supplementary Material online.

To carry out the MI analysis between the two clusterings, D and T, both clusterings need to be partitions of the same data, and thus need to have the same length in total (Romano et al. 2014).

In our case, we compare the DEs defined by the slow modes of motion (D) with the themes (T), detected based on sequence similarity alone. DEs define all residues in a protein domain's structure, so in order to carry out the MI analysis, the themes need to cover as many as possible of the residues in the protein domain structure. Also, there are gaps and overlaps between themes. With different threshold values for the overlaps/overlays and the gaps, we identify sequences of themes that maximally cover the whole structure. Because the thresholds are arbitrary, we used values of 3 and 5 residues for the overlapping regions, and values of 8, 10, and 15 residues for gaps between themes. Thus, in total we consider three versions of decomposition of the protein domain into themes: 3-residue overlap and 8-residue gap; 5-residue overlap and 10-residue gap; and 5-residue overlap and 15-residue gap. We generate these sequences of the themes with a Monte Carlo like algorithm that enables us to produce all possible combinations with the given thresholds. As an example, the application of the above three threshold combinations to 2XYI resulted in 147, 755 and 2218 different theme combinations.

The AMI and SMI computations (equations A7 and A8 in the supplementary text, Supplementary Material online) were performed using the MATLAB code provided by Romano et al. (2014).

The MI results are presented as mean AMI and SMI values that are averaged over all theme combinations for each dynamic mode in a given protein domain. The maximum and minimum values are also shown, to reflect the spectrum of the variations in the AMI and SMI values and thus the potential capacity of the alignment of various theme combinations with different slow modes of motion. In a given pool of themes for a protein domain, while a specific theme combination may highly correlate with the DEs of a specific mode, another theme combination would more significantly align with the DEs of another mode.

### *P*-value Analysis

In each domain, we randomly sampled 1,000 sets of consecutive amino acid segments, corresponding to the themes found in the respective domain. The number of random consecutive amino acid segments for each set was taken as the average number of themes in a domain in our dataset, which is 30. The minimum and maximum lengths of the randomly generated amino acid segments correspond to the respective values of the themes observed in the domain dataset, i.e. 30 and 175 residues, respectively. Thus, the lengths of the randomly generated segments were in the range of 30 to 175 amino acids.

The Monte Carlo-based algorithm was then used to obtain combinations of randomly generated amino acid segments. A randomly generated set is considered valid only if there is at least one combination of segments that covers the entire domain, which is needed for MI calculations. For each domain, the random set generation process was performed iteratively to obtain a thousand valid sets to get a sufficient population required for *P*-value analysis. The MI computations (supplementary equation A5, Supplementary Material online in the supplementary text, Supplementary Material online) were performed using the MATLAB code provided by Romano et al. (2014).

In order to obtain a single *P*-value for each individual mode in each domain, we considered the maximum MI values obtained from both random sets and themes combination. We obtained a distribution of MI values for each individual mode in each domain from the population of randomly generated sets of amino acid segments. *P*-value calculations were performed with the cumulative distribution functions of the distributions. Probability of obtaining a value *X* greater than the sample value (*x*), *P*-value, was calculated as;


P−value=P(X>x)=1−P(X<x)=1−F(x),

where *x* is the maximum MI obtained with the real themes (sample value) and *F* is the cumulative distribution function of the distribution obtained with the random generated population. *P*-value calculations were done via the “tcdf” function of MATLAB. Detailed results of the *P*-value analysis, codes and specifications are provided in the Supplementary website (https://gabiaxel.github.io/themes-dynamics/).

## Supplementary Material

msae184_Supplementary_Data

## Data Availability

The data underlying this article are available in the article and in its online Supplementary material (Supplementary Website: https://gabiaxel.github.io/themes-dynamics/ and supplementary text and figures).
