# Supplementary material for: Reused Protein Segments Linked to Functional Dynamics
Source: Mol Biol Evol. 2024 Sep 3;41(9):msae184. doi: 10.1093/molbev/msae184 (PMC11412252; doi:10.1093/molbev/msae184)
Supplement: msae184_Supplementary_Data [file msae184_supplementary_data.zip › Supplementary Text & Figures.pdf]

Reused Protein Segments Linked to Functional Dynamics

Yiğit Kutlu[1], Gabriel Axel[2], Rachel Kolodny[3,*], Nir Ben-Tal[2,*], Turkan Haliloglu[1,*]

[1]Department of Chemical Engineering and Polymer Research Center, Bogazici University, Istanbul, Turkey.

[2]Department of Biochemistry and Molecular Biology, George S. Wise Faculty of Life Sciences, Tel Aviv University, Tel Aviv, Israel.

[3]Department of Computer Science, University of Haifa, Haifa, Israel.

[*] To whom correspondence should be addressed. E-mail: trachel@cs.haifa.ac.il bental@tauex.tau.ac.il halilogt@boun.edu.tr

Supplementary Text

Correspondence between themes and Pfam entries

As noted in the main text, to detect the themes, we used an approach similar to that used to detect Pfam domains (Finn et al., 2014): namely, sequence analysis within the framework of hidden Markov models (Soding 2005). Accordingly, for completeness, we examine the relationship between the themes and Pfam entries. HMM-HMM search of the themes against the Pfam database results in 9,917 theme profiles (78% of all themes) matching one or more Pfam entries at 90% probability or higher (Figure S29). The vast majority of these theme profiles match only one or a few Pfam entries, with 3,395, 3,147 and 1,316 theme profiles matching 1, 2 and 3 Pfam entries, respectively (Figure S30). Similarly, the majority of the 3,695 theme-matching Pfam entries match one or few theme profiles, with 972, 432, 281 and 201 Pfam entries matching 1, 2, 3 and 4 theme profiles (Figure S31). Overall, both Pfam entries and themes reflect similarities in protein space. The main difference between these two types of sequence profiles is that our themes are inclusive, allowing for the same protein segment to be a part of multiple themes, while Pfam entries undergo post-processing to eliminate this, making them exclusive. Due to our inclusive approach, in a typical ECOD domain (at least in our set), each residue is covered by at least one theme (and many residues are covered by multiple themes). Pfam entries, on the other hand, provide only partial coverage. The differences are clearly visible in the example of Figure 1, and the supplementary website, where themes and Pfam entries are mapped to all the ECOD domains in our sets.

Correspondence between DEs and Pfam entries

Of the 150 ECOD domains in our dataset, only 61 can be defined with Pfam entries using the approach that we used with the themes. Unfortunately, 55 of these domains are defined by a single Pfam entry and 2 of them are defined by repetition of the same Pfam entry. This leaves us with 4 ECOD domains in which the same definition of overlap and gap residues can be used to examine the correspondence between Pfam entries and DEs. These four ECOD domains are e4kliA3, e2bcqA3, e4drxF1, and e3ro3A1, and interestingly, in each of these four cases, the Pfam entry combinations correspond to DEs of one of the seven slowest modes. AMI analysis on these four domains shows that the correspondence of the partitioning into Pfam entries with the DEs is as high as that of the themes (Table S14). The correspondence in 2D and 3D is shown in the respective pages in the supplementary website (https://gabiaxel.github.io/themes-dynamics/).

Impact of protein conformational changes on GNM modes and theme correspondence

The GNM analysis, used to detect the DEs, depends on the particular conformation used for the domain. Starting from various conformations of the same domain would yield various partitions into DEs, which may differ from each other in their correlations with the themes of the domain. (Obviously, the themes, derived from sequence analysis, would be exactly the same for all the conformations.) To examine the effect of different conformations on the DE-themes correlation, we conducted a thorough analysis of our ECOD domain dataset to identify and evaluate any significant structural changes. First, we scanned the dataset to identify other ECOD domains belonging to the same protein (as indicated by at least 99% sequence identity) that exhibit at least a moderate structural change, defined as an RMSD greater than 2.5 Å. Out of the 150 ECOD domains in our dataset, 145 do not have a counterpart ECOD domain showing significant structural changes. This leaves only five ECOD domains with counterparts exhibiting moderate structural changes. That only a handful of domains undergo conformational changes suggests that for most proteins, comprising multiple domains, conformational alterations are based on motions of the domains as rigid parts. (However, it is important to note that even relatively minor conformational changes can be important for the physiological function of the domain.) Among the five domains with alternative conformations, three have counterparts with significant differences in sequence lengths due to missing or uncaptured parts in the 3D structures. In these cases, the themes cover regions larger than the length of the sequence, and the differences in sequence lengths result in the segments obtained from GNM and the DEs defining different regions. Consequently, it is not feasible to compare the GNM modes or the correspondence between themes and DEs for these three domains.

This leaves us with two domains that can be meaningfully compared: e4qvhB1 and e1twfA10. Their counterparts, which exhibit moderate structural changes, are e4blaB3 and e3j1nA7, with RMSD values of 3.96 Å and 3.15 Å respectively. We conducted the exact same analysis using the two structurally-altered counterparts, and compared the results to the results obtained for the corresponding conformations.

Figure S1 illustrates the correlation matrices of the GNM modes for these domain pairs. For ECOD domain e4qvhB1, we found that six out of seven GNM modes have corresponding modes (correlation coefficient > 0.75) in its conformationally changed counterpart, e4blaB3, and vice-versa. For ECOD domain e1twfA10, all seven GNM modes have corresponding modes in its conformationally changed counterpart, e3j1nA7, and vice-versa. Similar to the observation with

homologous protein domains (Figure 13), we observed some shifts in mode space (e.g., the second dynamic mode of e1twfA10 corresponds to the third dynamic mode of e3j1nA7).

Subsequently, we projected the themes from the ECOD domains in our dataset onto their conformationally changed counterparts and calculated the AMI values to assess the correspondence between themes and DEs in these domains. We documented the maximum AMI values obtained for each mode in Tables S1 and S2, similarly to our treatment of ECOD domains within the dataset.

The fifth mode of domain e4qvhB1 lacks a corresponding mode in its counterpart domain e4blaB3, and conversely, the seventh mode of e4blaB3 has no counterpart in e4qvhB1. Despite these discrepancies, the DEs derived from these modes exhibit high correspondence with the themes. Conversely, the seventh modes of e1twfA10 and e3j1nA7 display a strong correlation, yet the resulting DEs differ, leading to a change in the correspondence with the themes. Notably, the seventh mode of e3j1nA7 exhibits a higher AMI value than e1twfA10. Despite minor differences between the GNM modes of ECOD domains and of their conformationally changed counterparts, resulting in slight modifications in DEs, our observations suggest that these differences do not significantly alter the correspondence between themes and DEs, at least not to an extent that would impact the findings of our study.

Mutual Information Equations

Variables are defined in Table S15.

$$H(\mathrm{D}) = -\sum_{i=1}^{k} \frac{d_i}{N} \log \frac{d_i}{N} \qquad \textit{entropy of D} \tag{A1}$$

$$H(\mathrm{D},\mathrm{T}) = -\sum_{i=1}^{k}\sum_{j=1}^{l} \frac{n_{ij}}{N} \log \frac{n_{ij}}{N} \qquad \textit{joint entropy of D and T}; \tag{A2}$$

$$H(\mathrm{D}|\mathrm{T}) = -\sum_{i=1}^{k}\sum_{j=1}^{l} \frac{n_{ij}}{N} \log \frac{n_{ij}/N}{t_j/N} \qquad \textit{conditional entropy of D given T}; \tag{A3}$$

$$MI(\mathrm{D},\mathrm{T}) = \sum_{i=1}^{k}\sum_{j=1}^{l} \frac{n_{ij}}{N} \log \frac{n_{ij}/N}{d_i t_j/N^2} \qquad \textit{mutual information of A and B} \tag{A4}$$

$$MI(\mathrm{D},\mathrm{T}) = H(\mathrm{D}) - H(\mathrm{D}|\mathrm{T}) = H(\mathrm{D}) + H(\mathrm{T}) - H(\mathrm{D},\mathrm{T}) \tag{A5}$$

$$E\{MI(\mathrm{D},\mathrm{T})\} = \sum_{i=1}^{k}\sum_{j=1}^{l} \sum_{n_{ij}=\max(d_i+t_j-N,0)}^{\min(d_i,t_j)} \frac{n_{ij}}{N} \log\left(\frac{N.n_{ij}}{d_i t_j}\right) \frac{d_i!\,t_j!\,(N-d_i)!\,(N-t_j)!}{N!\,n_{ij}!\,(d_i-n_{ij})!\,(t_j-n_{ij})!\,(N-d_i-t_j+n_{ij})!} \tag{A6}$$

$E\{MI(\mathrm{A},\mathrm{B})\}$ is the expected mutual information;

$$AMI(\mathrm{D},\mathrm{T}) = \frac{MI(\mathrm{D},\mathrm{T}) - E\{MI(\mathrm{D},\mathrm{T})\}}{\sqrt{H(\mathrm{D})\cdot H(\mathrm{T})} - E\{MI(\mathrm{D},\mathrm{T})\}} \tag{A7}$$

$$SMI(\mathrm{D},\mathrm{T}) = \frac{MI(\mathrm{D},\mathrm{T}) - E\{MI(\mathrm{D},\mathrm{T})\}}{\sqrt{Var\{MI(\mathrm{D},\mathrm{T})\}}} \tag{A8}$$

where $\sqrt{Var\{MI(\mathrm{D},\mathrm{T})\}}$ is the variance of mutual information.

Supplementary Figures & Tables

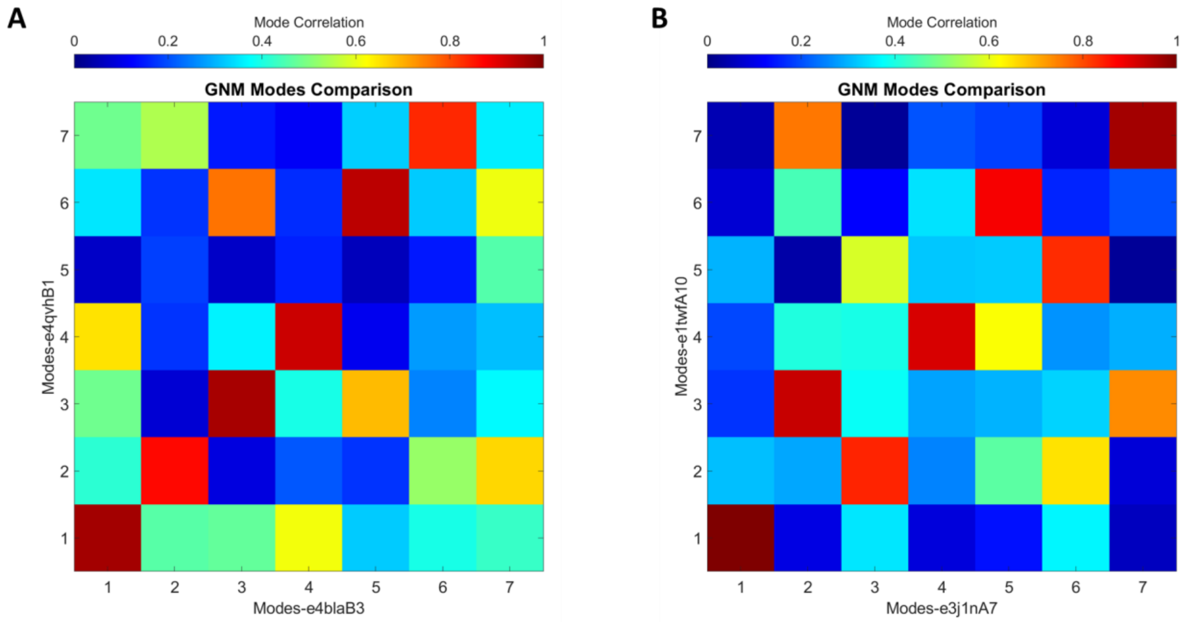

Figure S1. GNM mode comparisons of the ECOD domains in the dataset and their conformationally changed counterpart ECOD domains. The correlation coefficient between modes is represented with colors where dark red is 1, green is 0.5 and dark blue is 0.

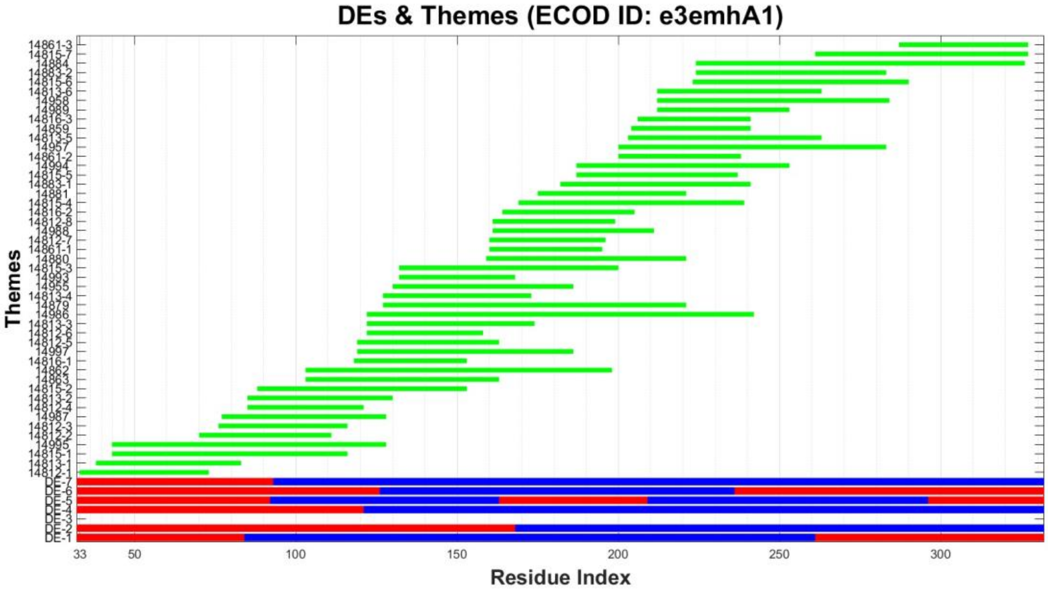

Figure S2. The DEs of the e3emhA1 propeller in each of the seven slowest modes (DE1-through-DE7) are marked along the Residue Index (x-axis). The dynamic segments of mode 3 are too short to yield DEs. The recurring sequences (‘themes’) are marked along the y-axis and their positions are highlighted in green.

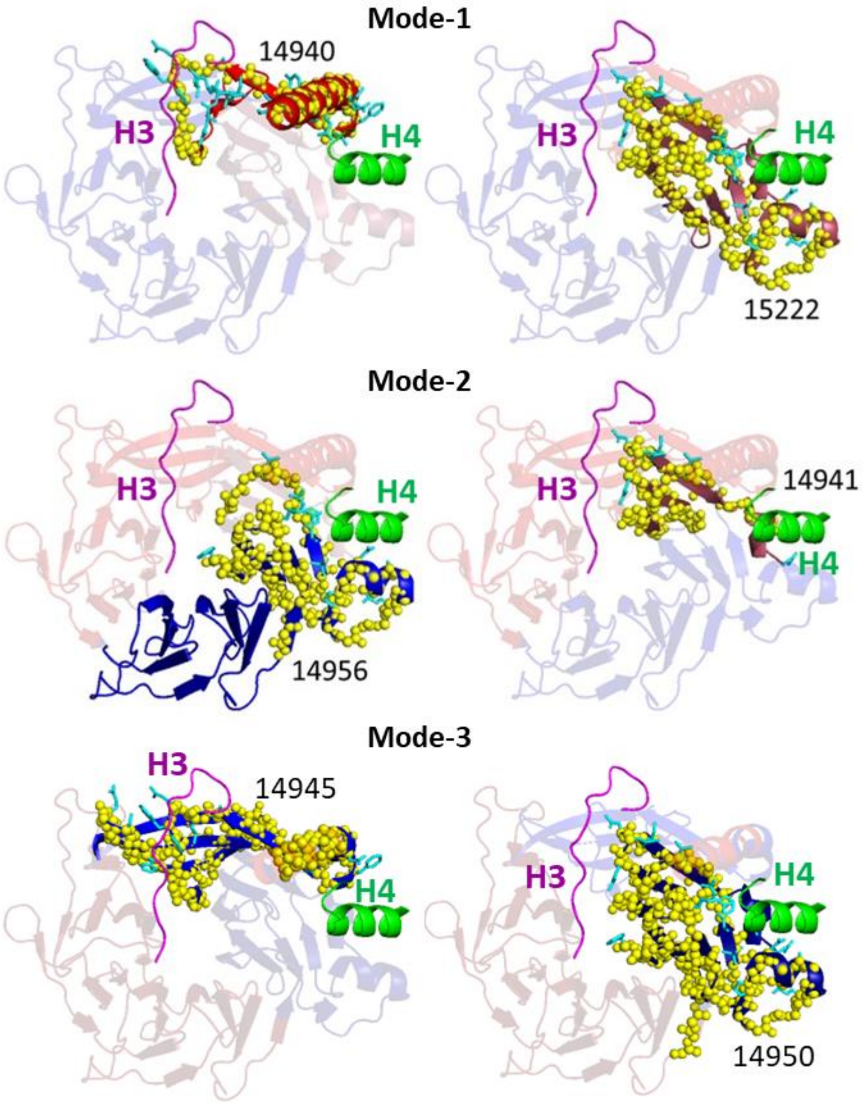

Figure S3. Putative evolutionary roots of allostery: DEs that appear to mediate binding of two histone peptides correspond to shared themes. The three slowest modes of motion observed in the e2xyiA1 β–propeller domain of the CAF1 protein (PDB ID: 2XYI) in complex with histone H3 peptide (magenta) and histone H4 peptide (green). The DEs are colored in shades of red and blue with the sidechains of peptide binding amino acids marked with cyan sticks. Themes that correlate with the DEs and appear to mediate binding of both peptides are highlighted with yellow spheres. The H4 peptide is included in the e2xyiA1 structure, and the H3 peptide is brought in from PDB ID 2YBA by superimposing the CAF1 propellers of both structures.

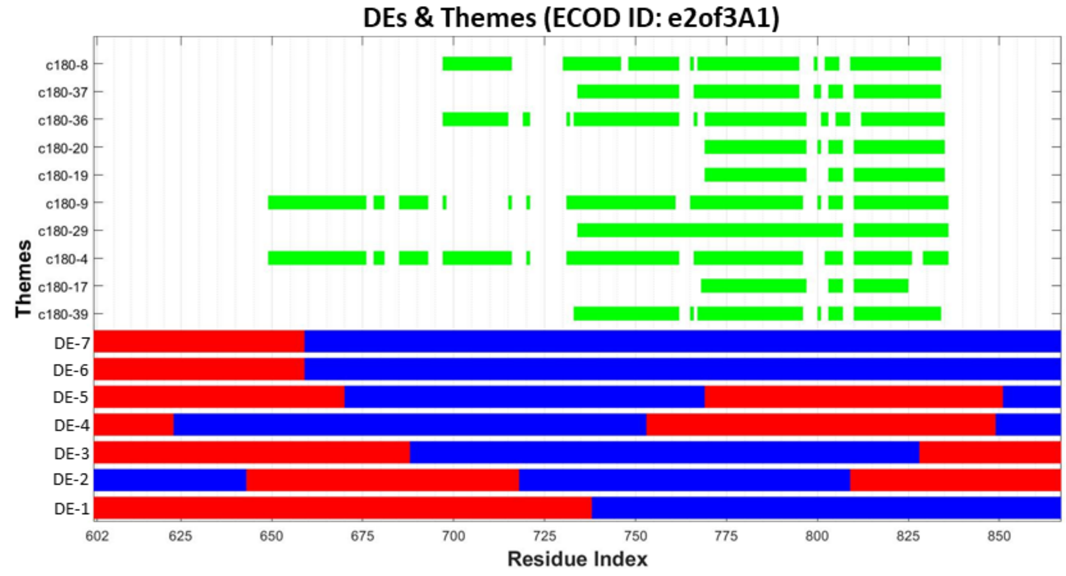

Figure S4. DEs and themes in ARM-repeat (PDB ID: 2OF3, ECOD domain ID: e2of3A1)*.

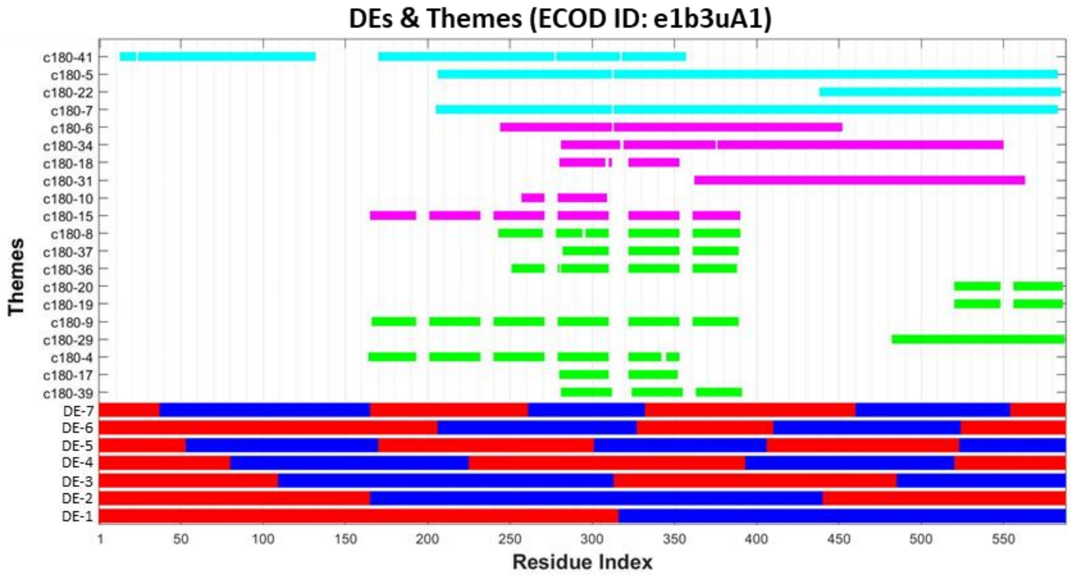

Figure S5. DEs and themes in ARM-repeat (PDB ID: 1B3U, ECOD domain ID: e1b3uA1)*.

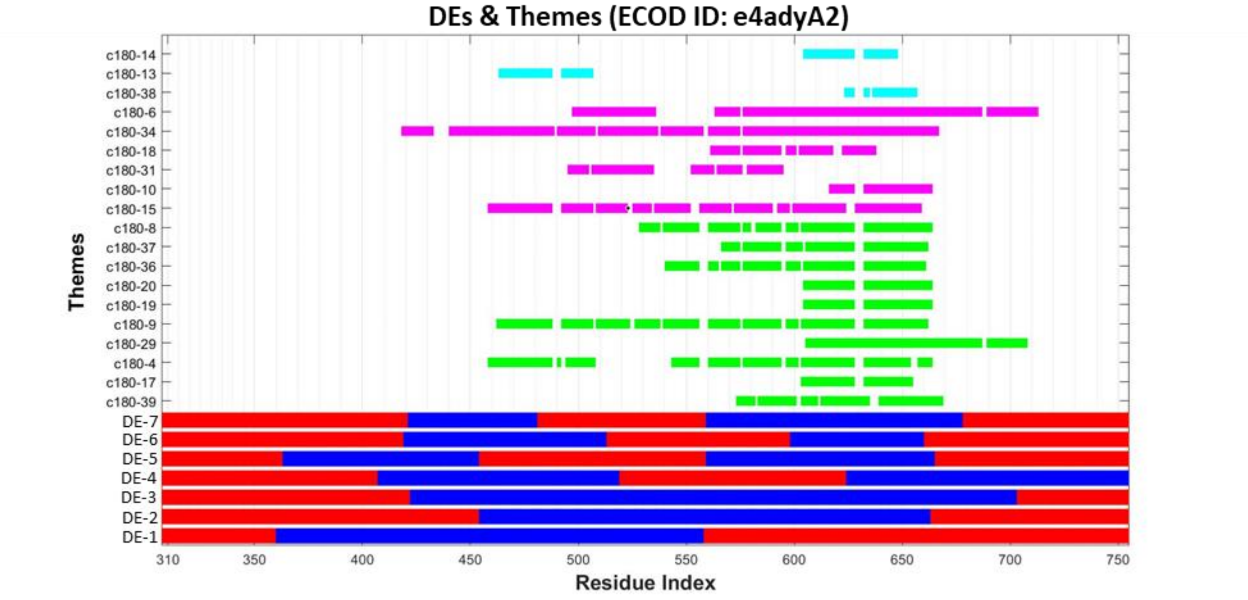

Figure S6. DEs and themes in proteasome/cyclosome repeat (PDB ID: 4ADY, ECOD domain ID: e4adyA2)*.

*For Figures S4 to S6: Themes shared between all three helix-bundle structures (2OF3, 1B3U and 4ADY) are marked green. Themes shared between 1B3U and 4ADY are in magenta. Themes that are unique to one of the three proteins are in cyan.

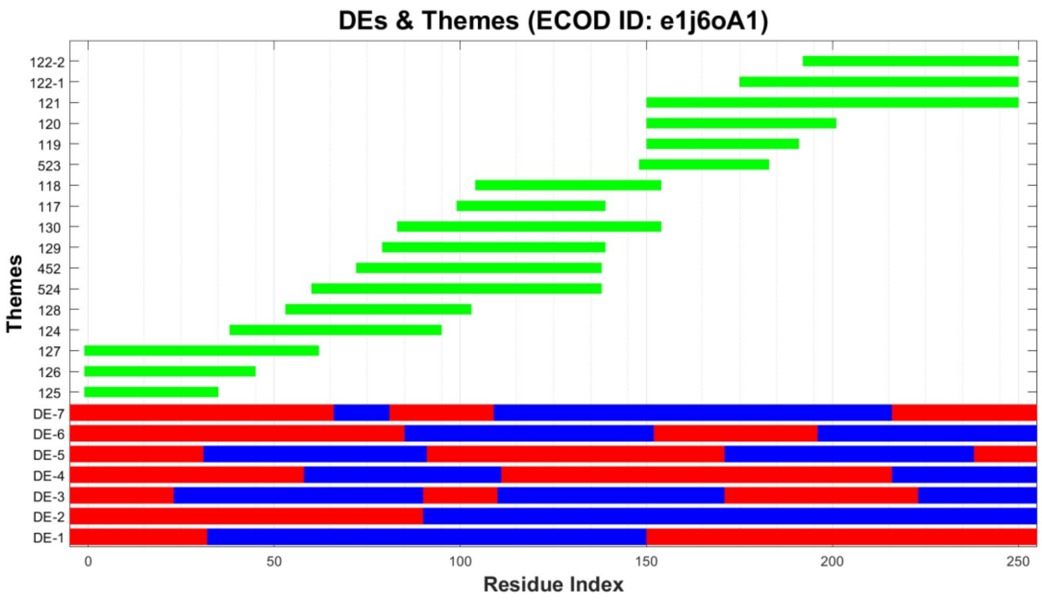

Figure S7. DEs and themes in TatD-related deoxyribonuclease (PDB ID: 1J6O, ECOD domain ID: e1j6oA1).

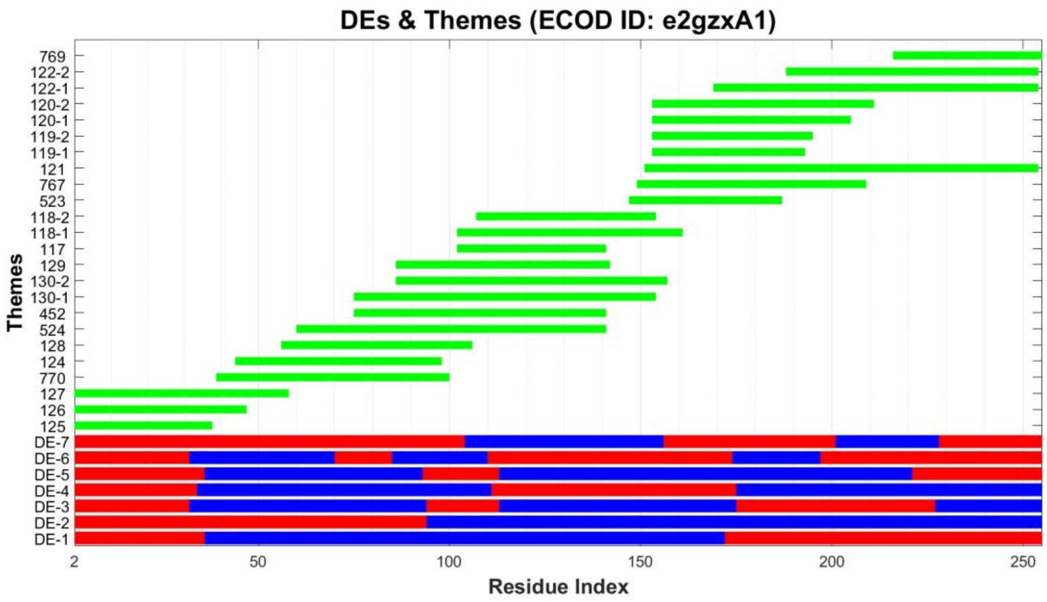

Figure S8. DEs and themes in Putative TatD related DNAse (PDB ID: 2GZX, ECOD domain ID: e2gzxA1).

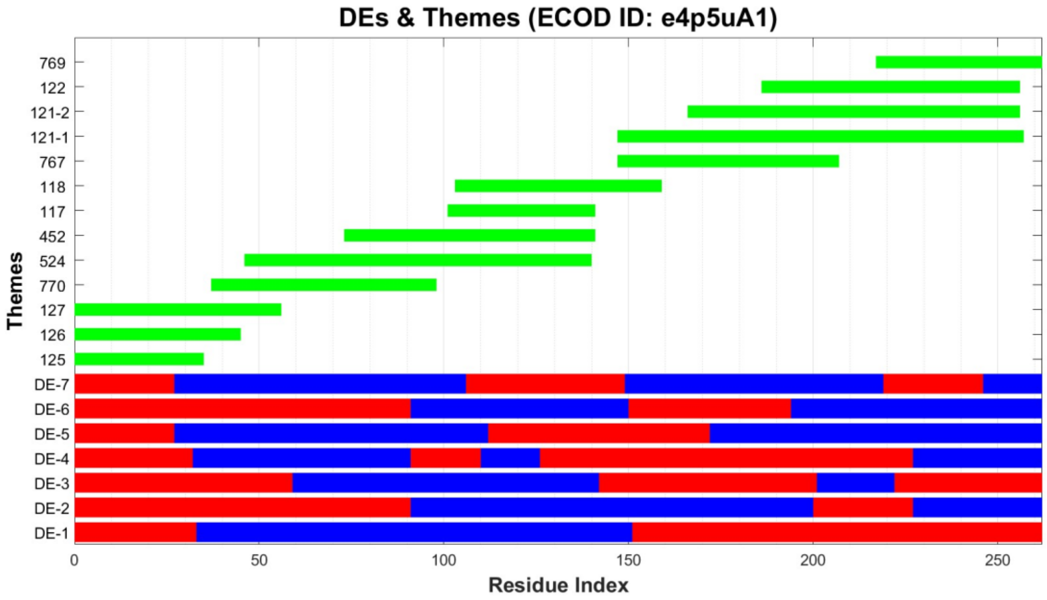

Figure S9. DEs and themes in Tat-linked quality control protein TatD (PDB ID: 4P5U, ECOD domain ID: e4p5uA1).

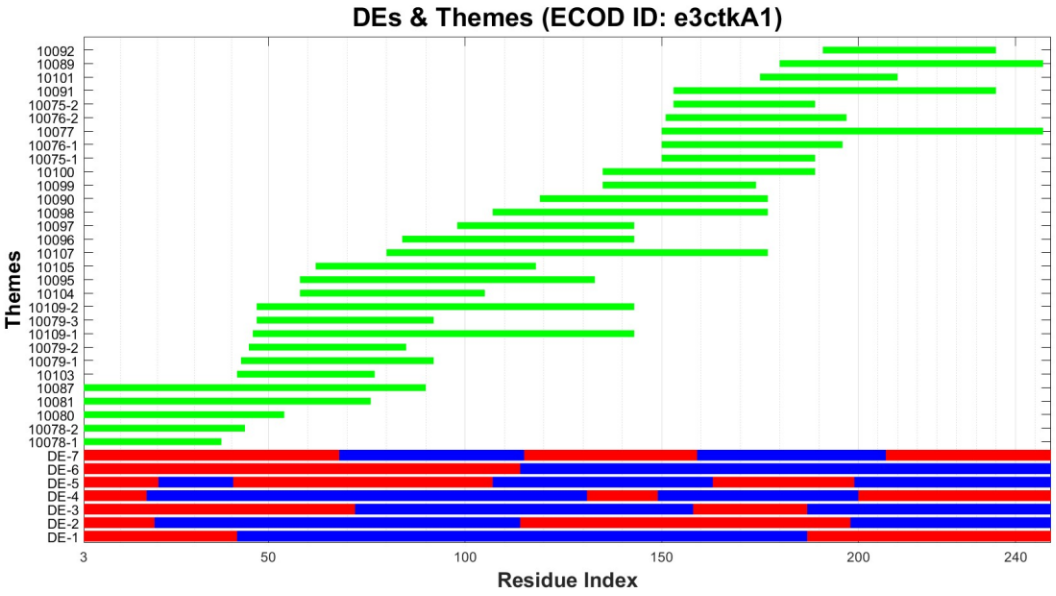

Figure S10. DEs and themes in rRNA N-glycosidase (PDB ID: 3CTK, ECOD domain ID: e3ctkA1).

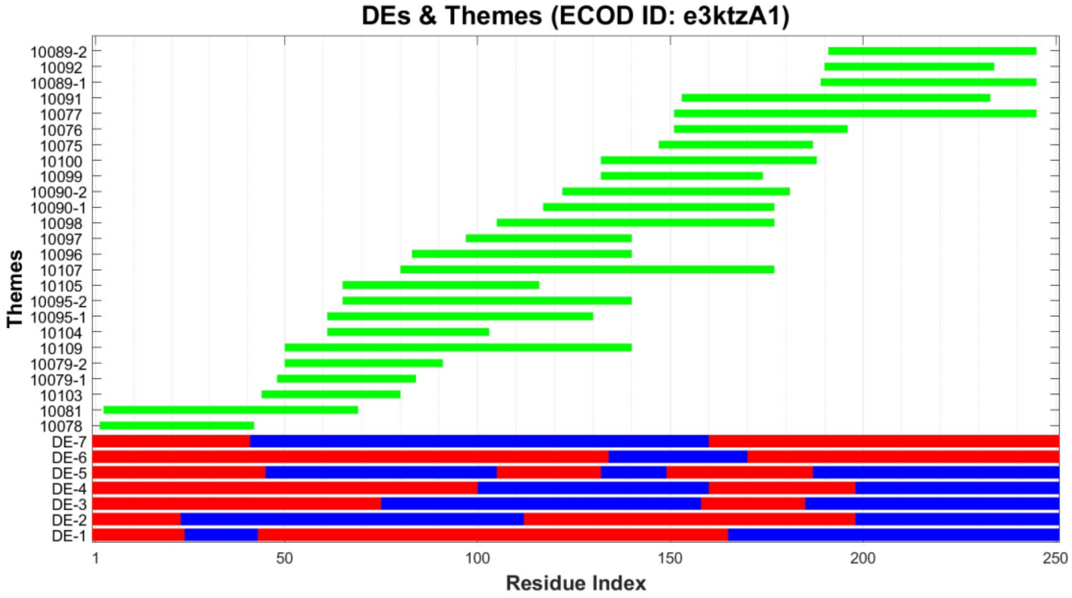

Figure S11. DEs and themes in Ribosome-inactivating protein gelonin (PDB ID: 3KTZ, ECOD domain ID: e3ktzA1).

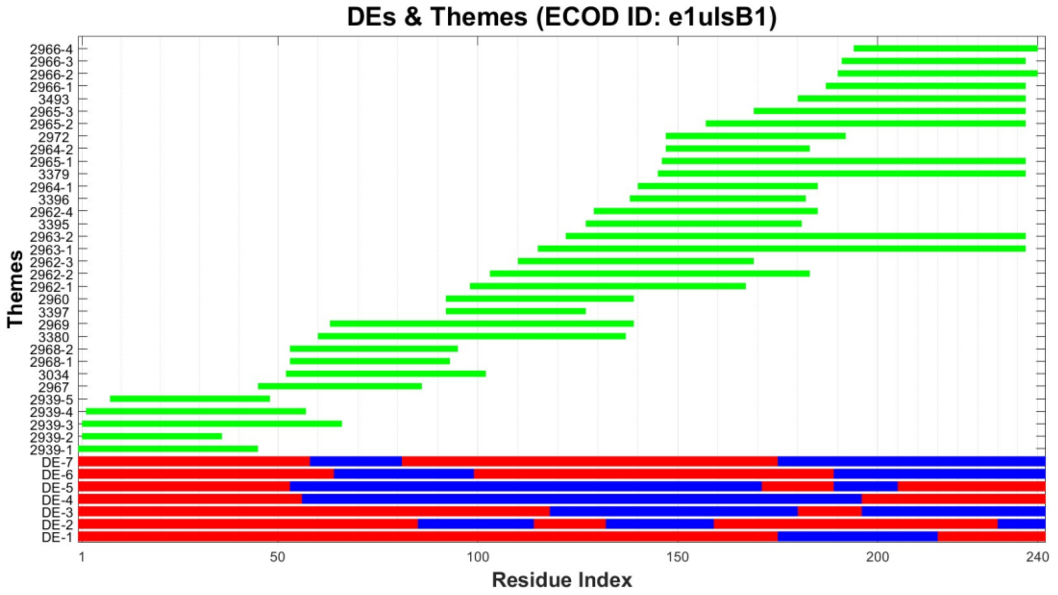

Figure S12. DEs and themes in Putative 3-oxoacyl-acyl carrier protein reductase (PDB ID: 1ULS, ECOD domain ID: e1ulsB1).

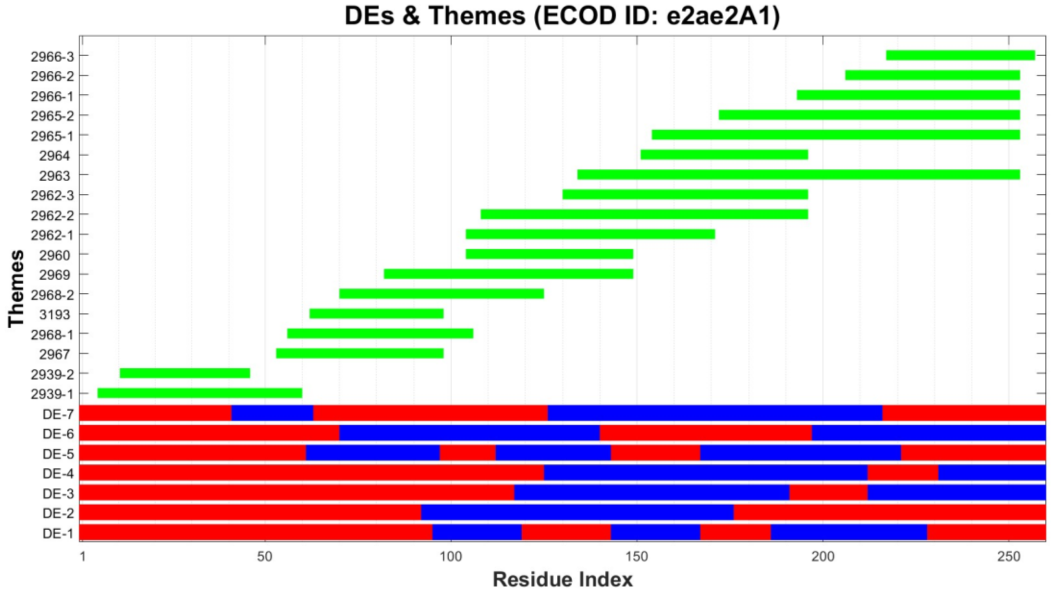

Figure S13. DEs and themes in Tropinpne reductase-II (PDB ID: 2AE2, ECOD domain ID: e2ae2A1).

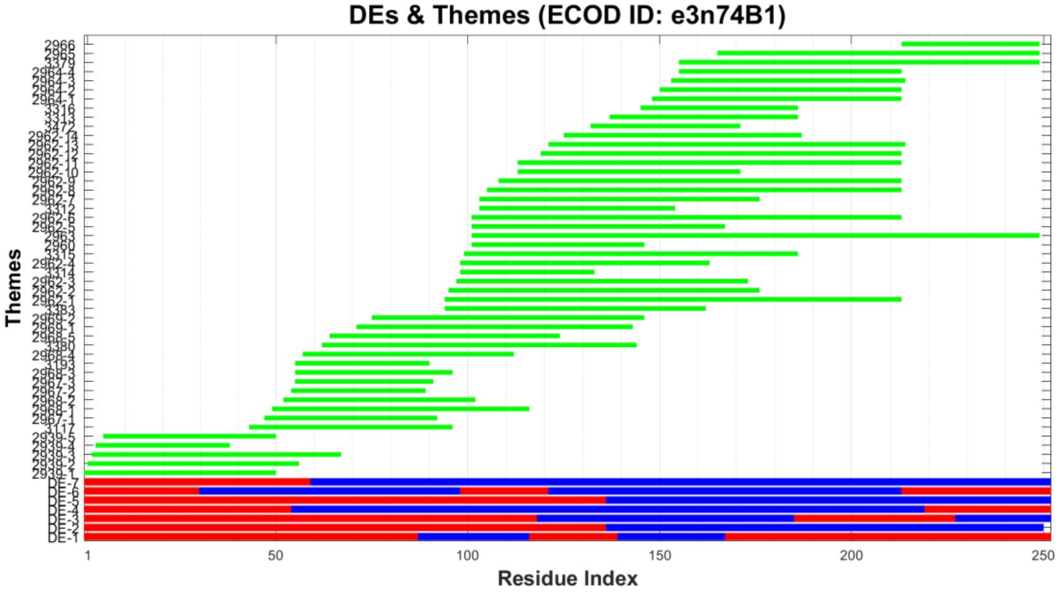

Figure S14. DEs and themes in 3-Ketoacyl-(Acyl-Carrier-Protein) reductase (PDB ID: 3N74, ECOD domain ID: e3n74B1).

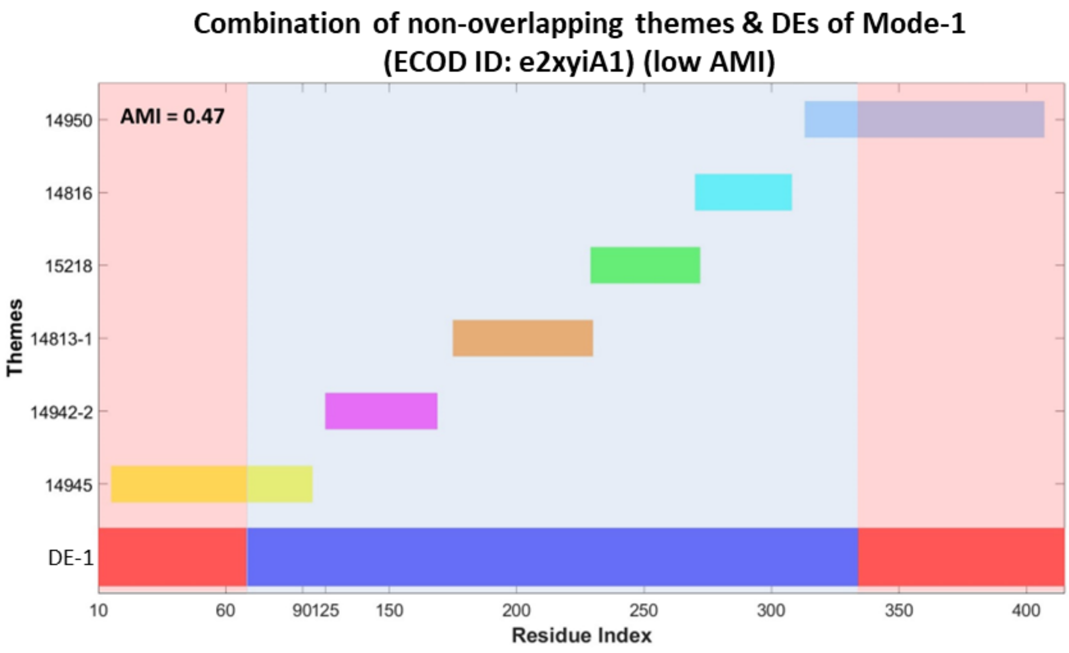

Figure S15. An exemplary case of low AMI (AMI < 0.50) correspondence between themes and DEs for the e2xyiA1 propeller.

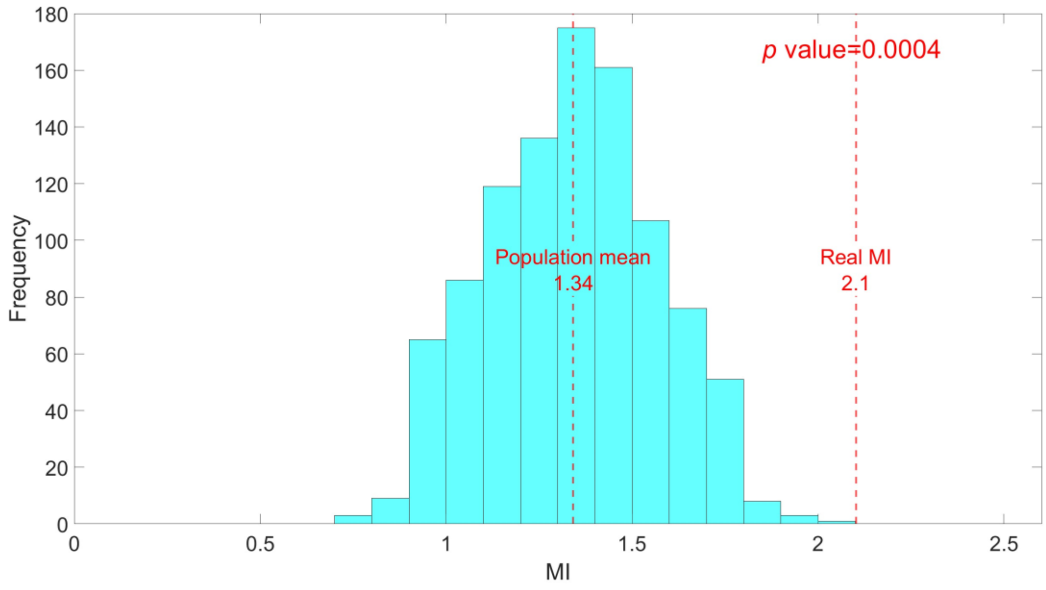

Figure S16. Probability distribution of MI for the randomly generated themes of the e2xyiA1 propeller and DEs of mode 5. The MI and p-values for the real themes are marked.

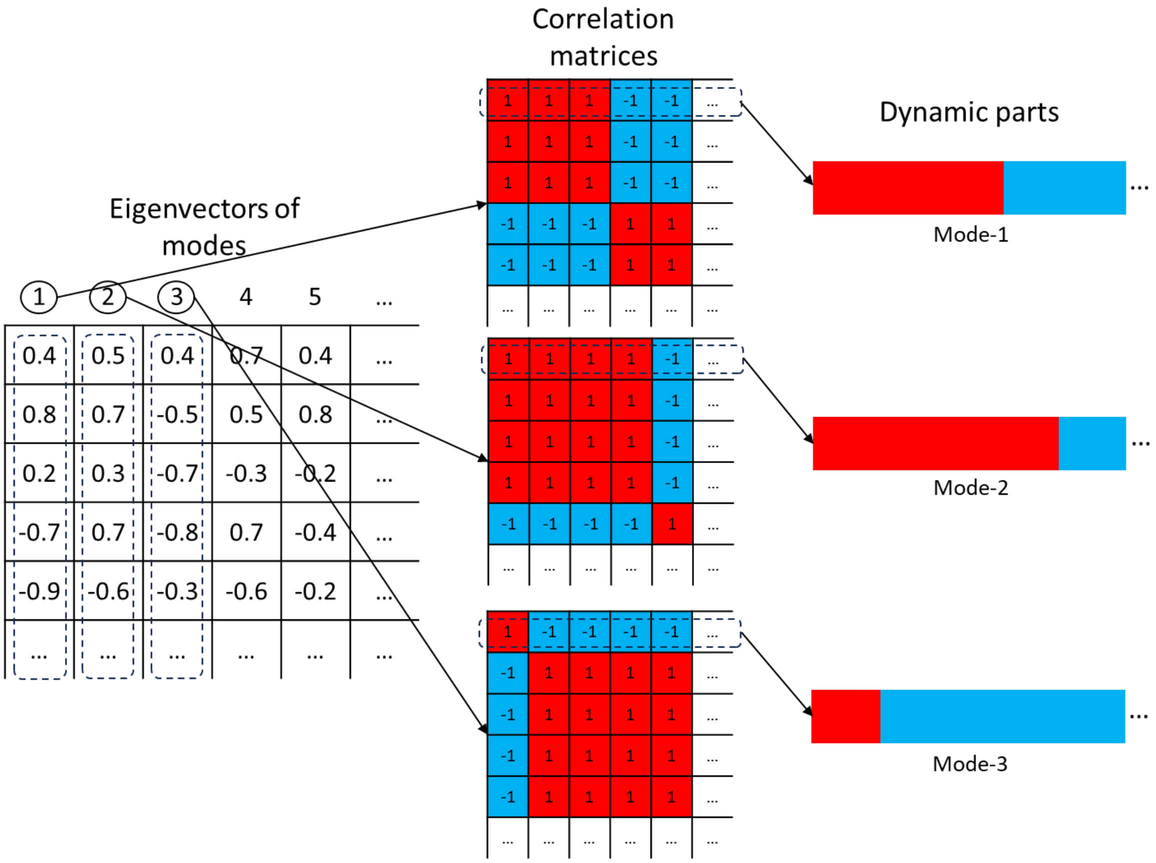

Figure S17. A toy model to represent decomposition of the structure into the dynamic parts from a correlation matrix of GNM. The leftmost panel represents the matrix ***U***, an orthogonal matrix, whose columns $\boldsymbol{u}_i$ are the eigenvectors of **Γ,** a Kirchhoff (connectivity) matrix. The middle panel represents the symmetric matrix of residue correlations composed of “+1” and “-1” for each mode which is obtained via equation 4. The rightmost panel represents the dynamic parts obtained from the matrix of residue correlations by taking any row or column; here, the first row is used.

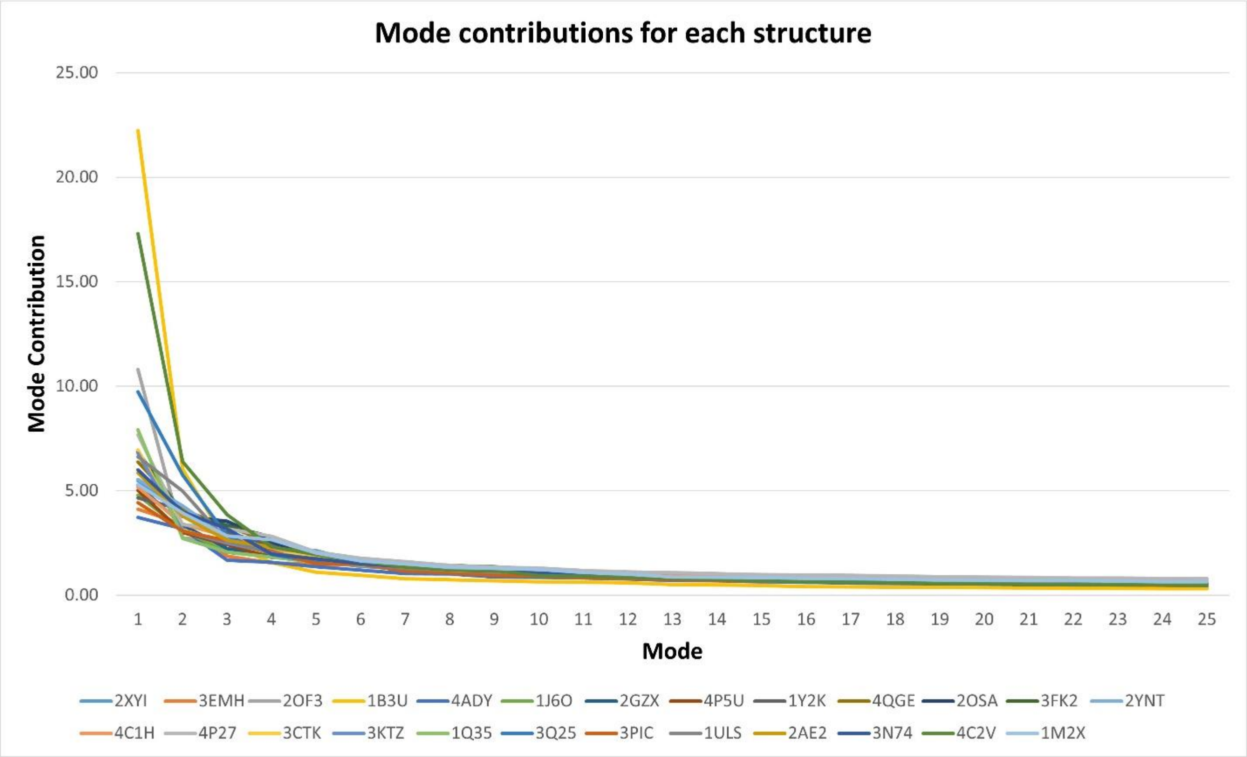

Figure S18. Relative contributions of the modes to the global dynamic motion of the protein. The PDB IDs and colors are listed.

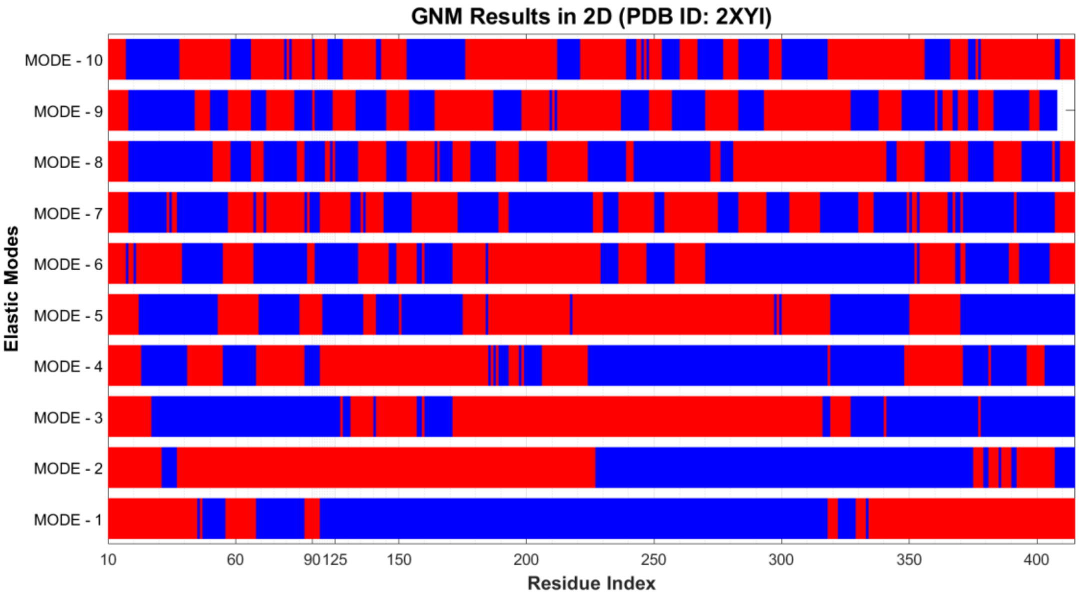

Figure S19. The ten slowest elastic modes of the e2xyiA1 propeller. We observe that the prevalence of larger segments decays with progression through the dynamic spectrum.

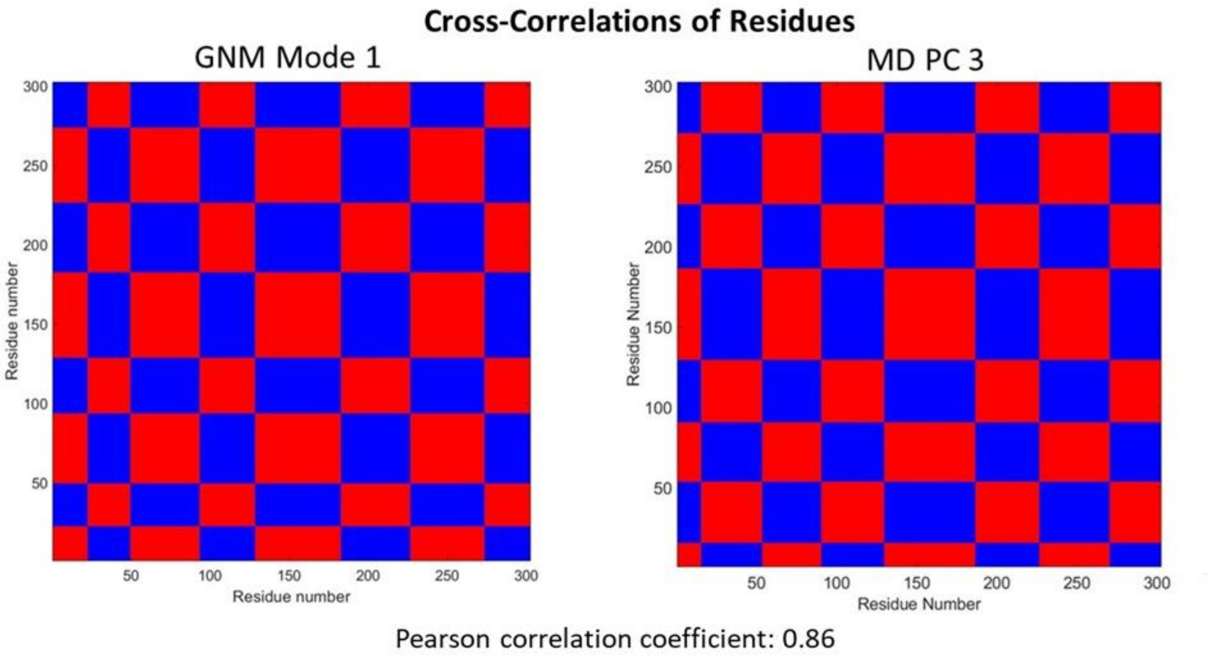

Figure S20. Comparison of individual GNM modes to principal components (PCs) obtained from MD simulations. One-to-one correspondence between the slowest GNM mode and PC3.

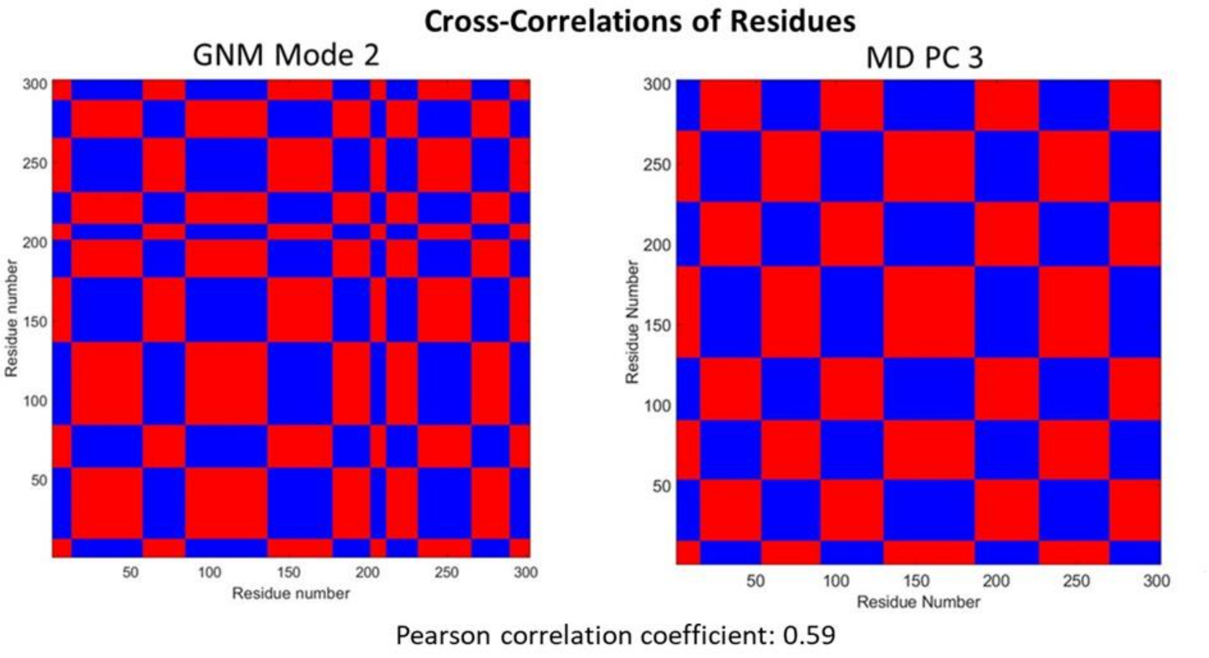

Figure S21. Comparison of individual GNM modes to PCs obtained from MD simulations. One-to-one correspondence between the second slowest GNM mode and PC3.

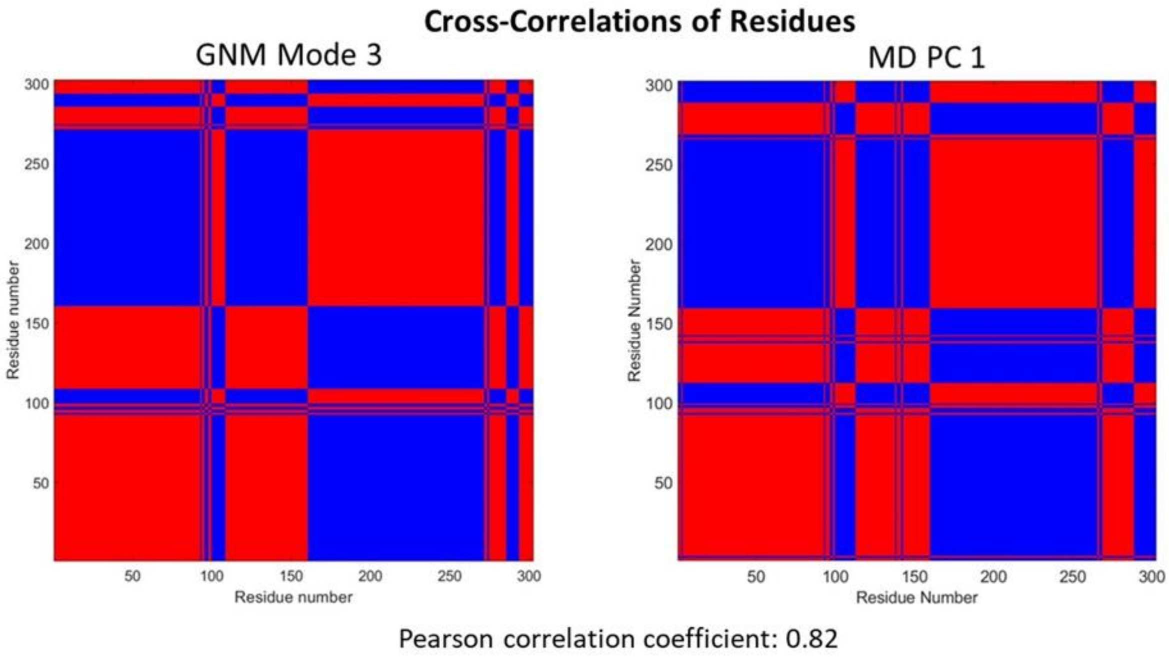

Figure S22. Comparison of individual GNM modes to PCs obtained from MD simulations. One-to-one correspondence between the third slowest GNM mode and PC1.

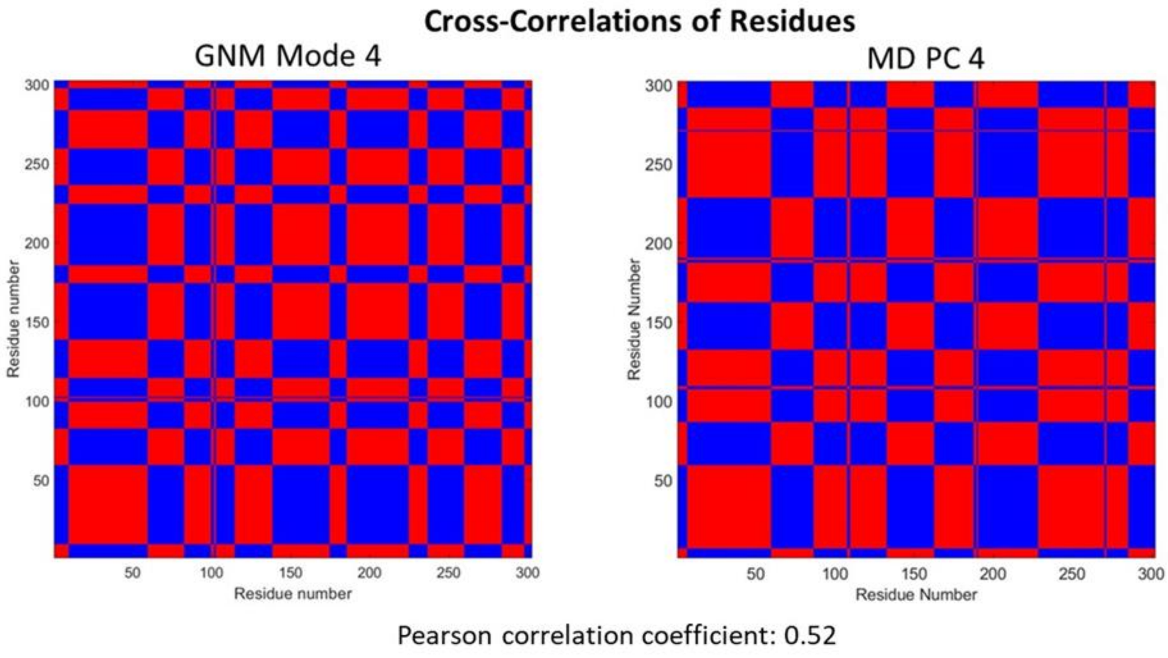

Figure S23. Comparison of individual GNM modes to PCs obtained from MD simulations. One-to-one correspondence with relatively low Pearson correlation coefficient between the fourth slowest GNM mode and PC4.

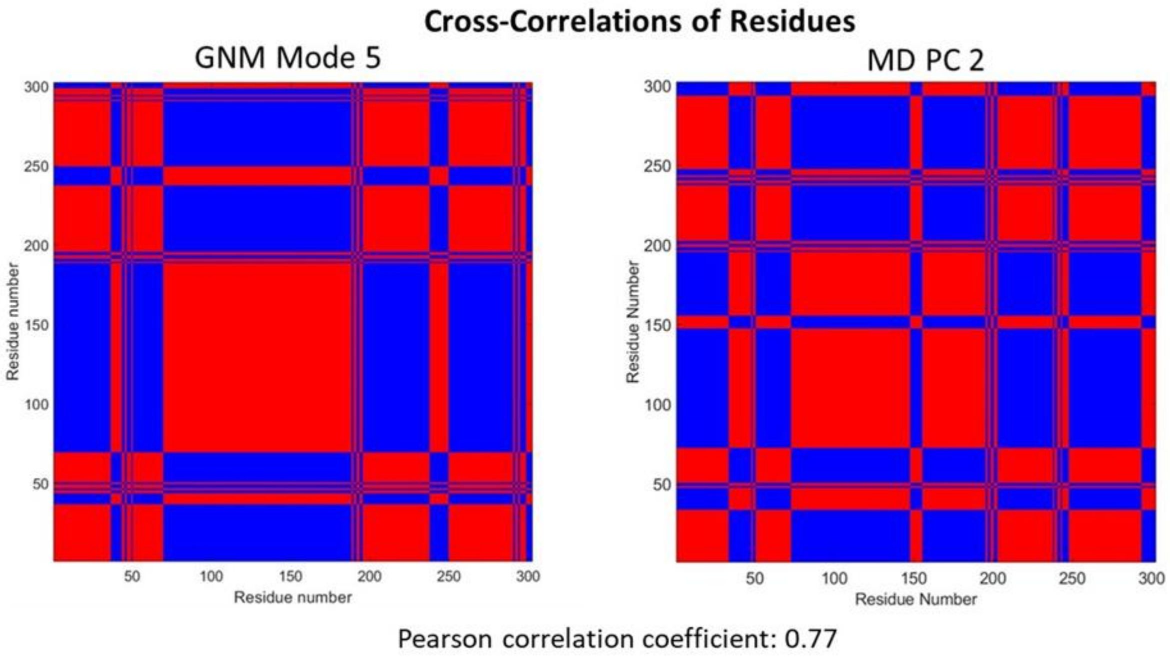

Figure S24. Comparison of individual GNM modes to PCs obtained from MD simulations. One-to-one correspondence between the fifth slowest GNM mode and PC2.

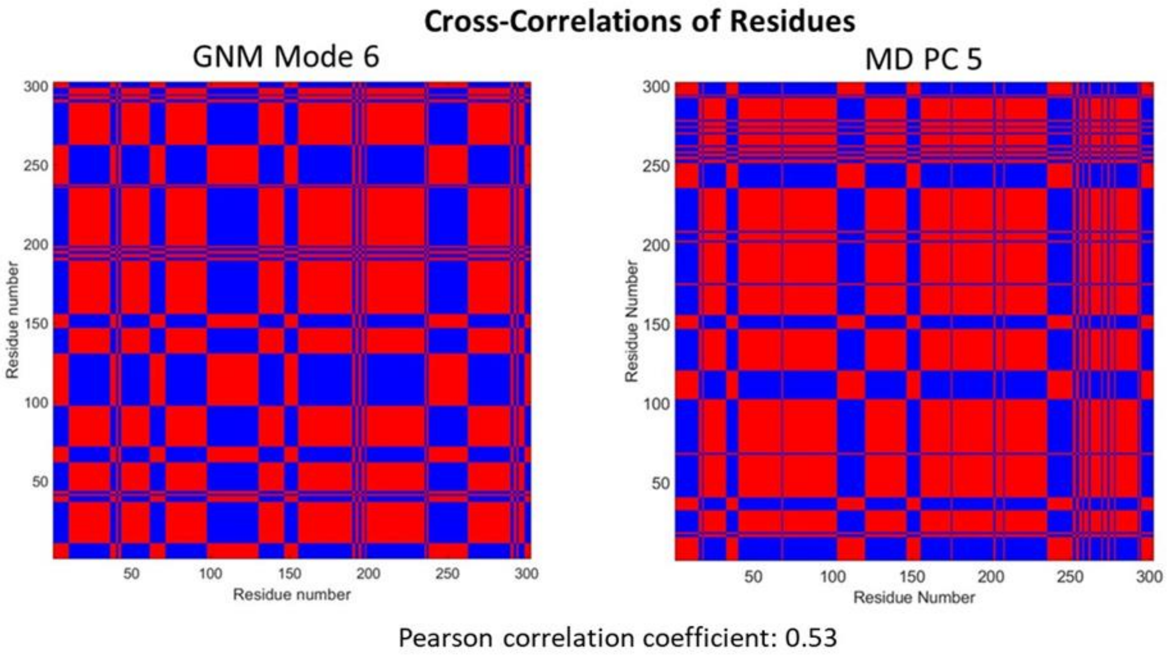

Figure S25. Comparison of individual GNM modes to PCs obtained from MD simulations. One-to-one correspondence with relatively low Pearson correlation coefficient between the sixth slowest GNM mode and PC5.

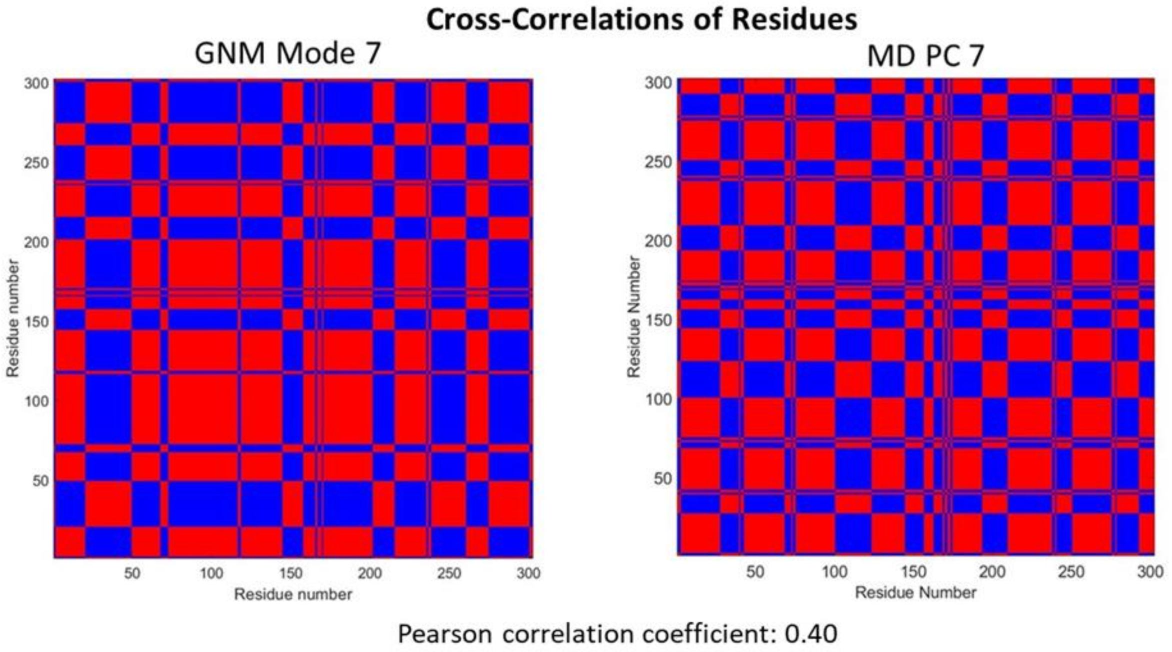

Figure S26. Comparison of individual GNM modes to PCs obtained from MD simulations. One-to-one correspondence with relatively low Pearson correlation coefficient between the seventh slowest GNM mode and PC7.

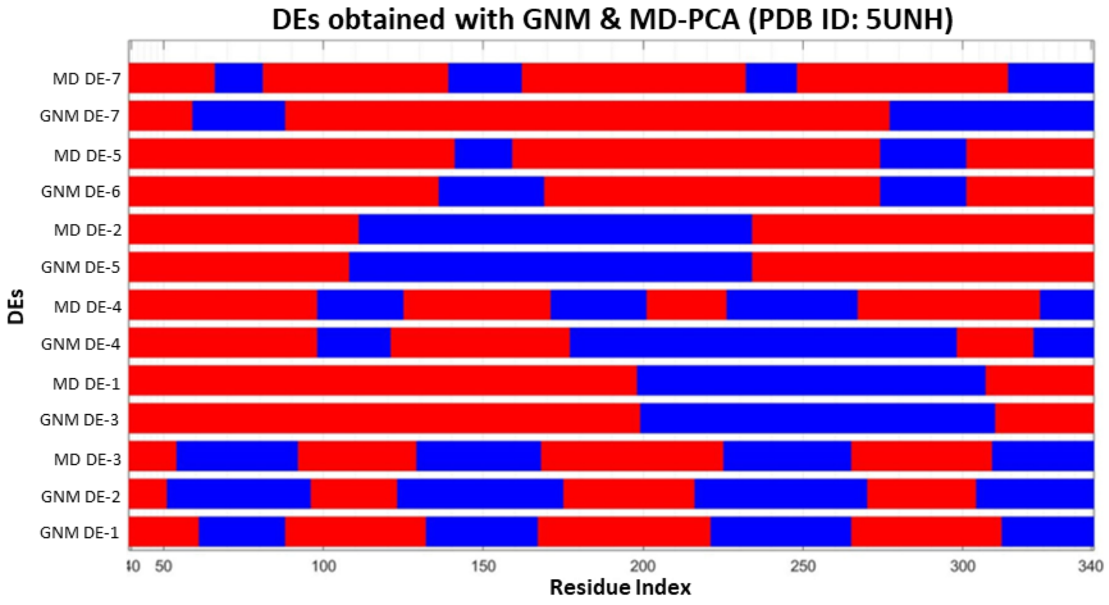

Figure S27. DEs obtained with GNM and MD-PCA. DEs obtained via two different methods are observed to be very similar, save differences between the seventh mode of GNM and PC7 of MD.

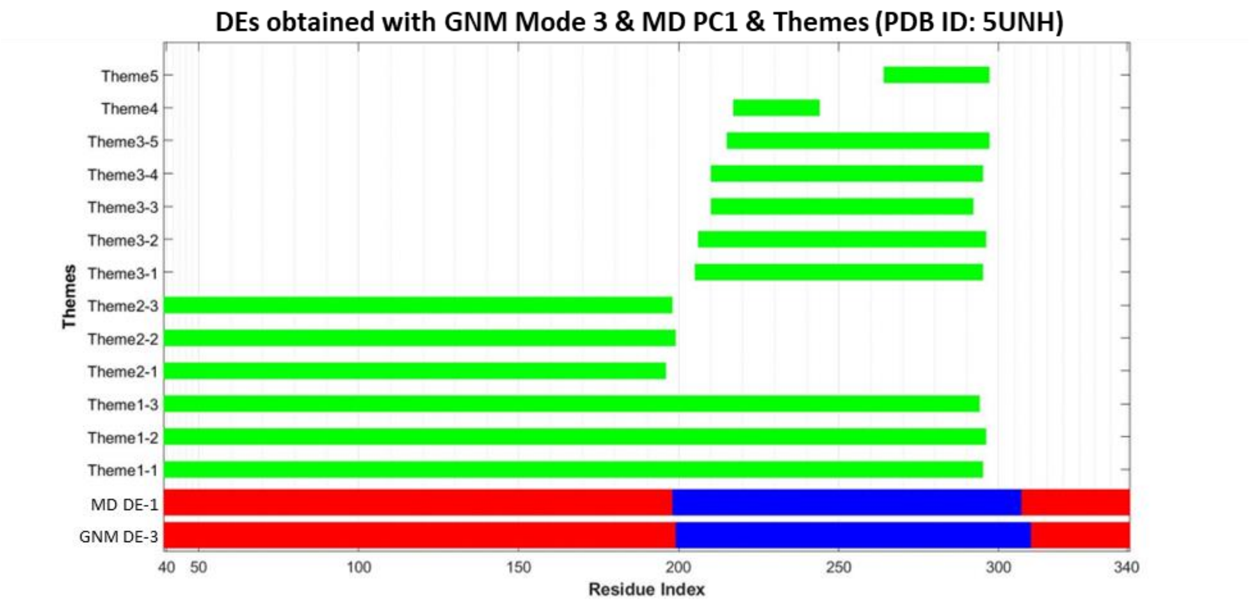

Figure S28. Themes and DEs of the first MD-PCA and the third slowest mode of GNM. Theme-1 corresponds to two DEs of the third slow mode GNM and first mode of MD-PCA. Theme-2 corresponds to the first DE and Theme-3 corresponds to the second DE of the same modes.

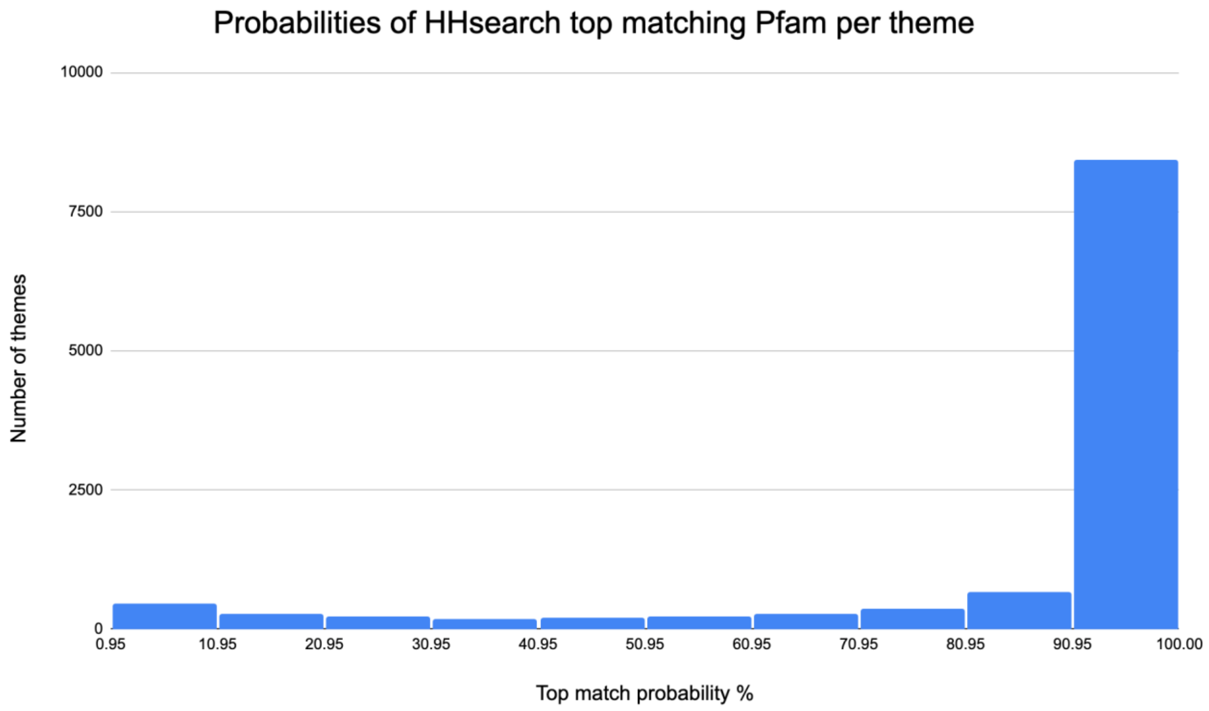

Figure S29. Correlation of themes and Pfam profiles: Number of theme HMM profiles grouped by the probability of their top match to a Pfam HMM profile. HMM-HMM search was implemented with HHsearch. The vast majority of themes match at least one Pfam entry with high probability (90%+).

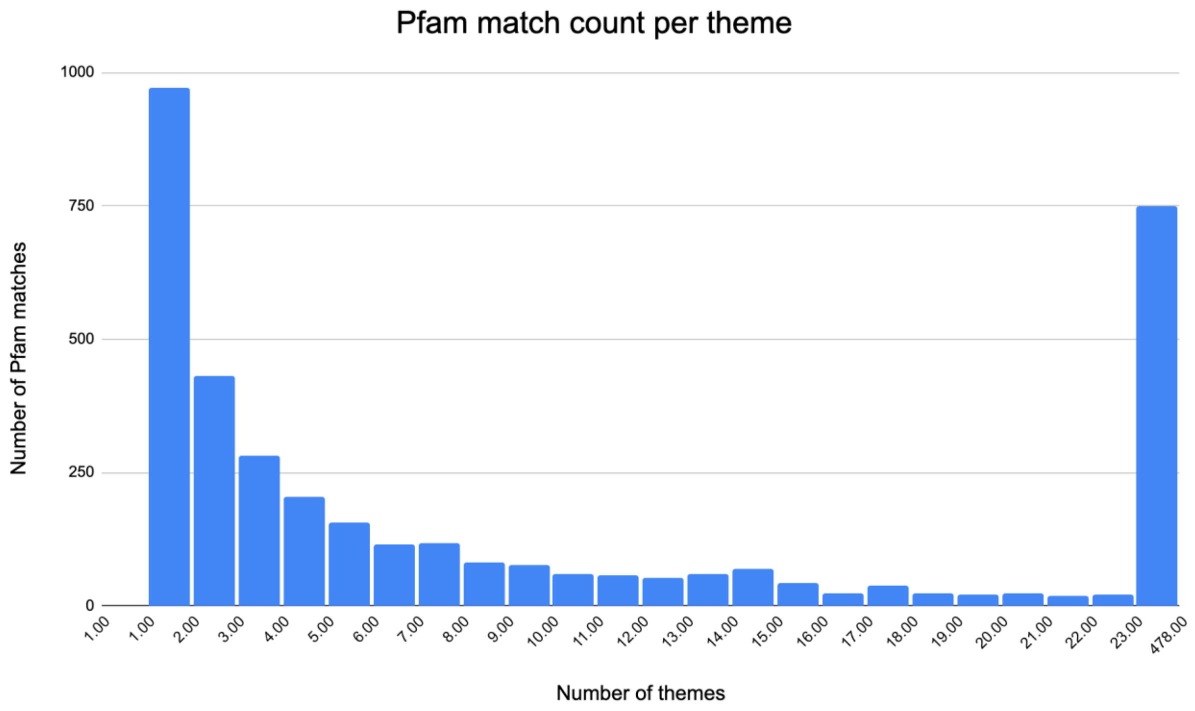

Figure S30. Number of Pfam HMM profiles grouped by the number of theme HMM profiles they match to with probability of 90% or higher (last column groups all counts of 23 and above). The vast majority of Pfam entries match one or few themes.

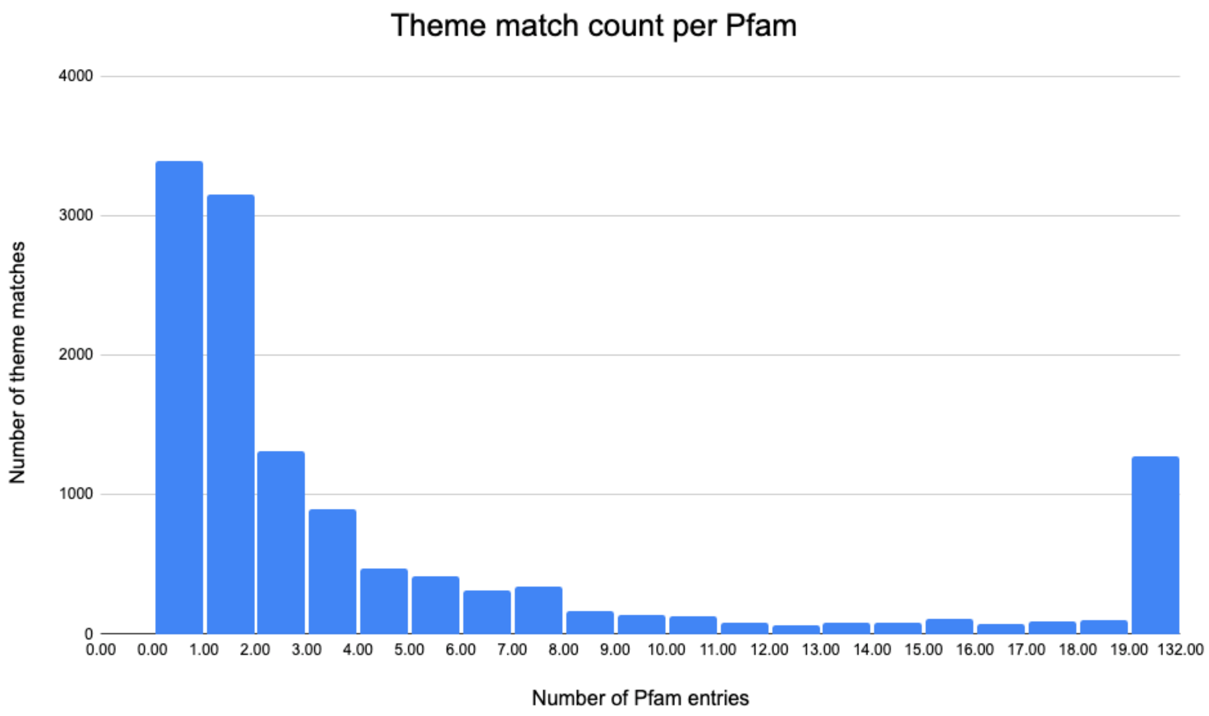

Figure S31. Number of theme HMM profiles grouped by the number of Pfam HMM profiles they match to with probability of 90% or higher (last column groups all counts of 19 and above). The vast majority of themes match one or few Pfam entries.

Domain ID	DEs Mode-1	DEs Mode-2	DEs Mode-3	DEs Mode-4	DEs Mode-5	DEs Mode-6	DEs Mode-7
**e4qvhB1**	0.73	0.69	0.79	0.68	0.70	0.75	0.64
**e4blaB3**	0.73	0.73	0.74	0.68	0.78	0.77	0.75

Table S1. The maximum AMI values for individual modes of e4qvhB1 and its counterpart domain e4blaB3.

Domain ID	DEs Mode-1	DEs Mode-2	DEs Mode-3	DEs Mode-4	DEs Mode-5	DEs Mode-6	DEs Mode-7
**e1twfA10**	0.34	0.53	0.66	0.53	0.66	0.73	0.40
**e3j1nA7**	0.31	0.67	0.52	0.53	0.63	0.78	0.67

Table S2. The maximum AMI values for individual modes of e1twfA10 and its counterpart domain e3j1nA7.

Theme Name	Position on e2xyiA1
**14940**	14-53 / 21-55 / 21-59
**14945**	16-120
**14952**	47-120
**14946**	55-120 / 58-91 / 60-119
**14942**	126-162 / 126-170
**14953**	126-181
**14944**	126-213
**14812**	176-213
**14954**	176-220
**14813**	176-231 / 176-240 / 227-287
**14937**	227-265
**14938**	227-309
**15218**	230-273
**15220**	230-297
**15221**	230-330
**14949**	243-297
**14815**	244-309 / 289-353 / 299-353 / 343-406
**14816**	271-309
**15219**	281-315
**14955**	283-341
**14861**	314-353 / 368-408
**14950**	314-408
**14956**	315-381
**15222**	336-407
**14951**	356-415
**14941**	376-411

Table S3. The themes detected in the e2xyiA1 propeller. In cases of nearly overlapping themes only one representative is used in order to minimize the computational work and confusion in figures. Full list of themes can be observed at https://trachel-srv.cs.haifa.ac.il/rachel/ppi/themes/

Theme Name	Position on e3emhA1	Position on e2xyiA1
**14812**	38-77 / 75-115 / 81-120 / 90-125 / 124-167 / 127-161 / 127-162 / 165-200 / 166-203	176-213
**14813**	43-87 / 82-132 / 90-134 / 127-178 / 132-177 / 166-224 / 208-267 / 217-257 / 217-267	176-231 /176-240 / 227-287
**14815**	48-120 / 93-157 / 137-204 / 174-243 / 192-241 / 228-294 / 266-331	244-309 / 289-353 / 299-353 / 343-406
**14816**	123-157 / 127-160 / 169-209 / 211-245	271-309
**14861**	165-199 / 205-242 / 292-331	314-353 / 368-407 / 368-408 / 369-408 / 371-407
**14955**	48-132	283-341
**14862**	108-202	-
**14863**	108-167	-
**14997**	124-190	-
**14986**	127-246	-
**14993**	137-172	-
**14880**	164-225	-
**14988**	166-215	-
**14881**	180-225	-
**14883**	187-245 / 229-287	-
**14994**	192-257	-
**14957**	205-287 / 217-288	-
**14859**	209-245	-
**14989**	217-257	-
**14958**	217-288	-
**14884**	229-330	-
**14995**	48-132	-
**14987**	82-132	-

Table S4. The themes detected in the e3emhA1 propeller. Themes that are shared with the e2xyiA1 propeller are listed first.

Theme ID	Position on e2of3A1	Position on e1b3uA1	Position on e4adyA2
c180-39	734-762,766,768-796,801,804-807,811-834	282-310,323-353,362-389	567-575,577-594,597-604,606-628,633-662
c180-17	769-797,804-807,811-835	281-310,323-352	604-628,633-665
c180-4	650-676,679-681,686-693,698-716,721,732-762,767-796,803-807,811-826,830-836	165-193,202-232,241-271,280-310,323-342,346-353	459-488,491-492,495-508,544-556,561-575,577-594,597-602,604-628,633-654,658-664
c180-29	735-807,811-836	483-587	606-687,690-708
c180-9	650-676,679-681,686-693,698-716,721,732-761,766-796,801,804-807,811-836	167-193,202-232,241-271,280-310,323-353,362-389	463-488,493-507,509-524,527-538,540-556,561-575,577-594,597-602,604-628,633-662
c180-19	770-797,804-807,811-835	521-548,557-586	605-628,633-664
c180-20	770-796,801,804-807,811-835	521-548,557-586	605-628,633-664
c180-36	698-715,720-721,732,734-762,766,768-795,800-801,804-807,811-833	252-271,280,282-310,323-353,362-388	541-556,561-565,567-575,577-594,597-603,605-628,633-661
c180-37	735-762,767-795,800-801,804-807,811-834	283-310,323-353,362-389	567-575,577-594,597-604,606-628,633-662
c180-8	698-716,731-746,749-762,766,768-796,801,804-807,811-835	244-270,279-294,297-310,323-353,362-390	529-538,540-556,561-575,577-580,583-594,597-602,604-628,633-664
c180-15		166-193,202-232,241-271,280-310,323-353,362-390	459-488,493-507,509-524,527-538,540-556,561-575,577-594,597-602,604-628,633-663
c180-10		258-271,280-309	617-628,633-664
c_180_31		363-583	496-507,509-537,565-575,577-688,691-707
c180-18		281-308,311-312,323-353	562-575,577-594,597-601,603-628,633-648
c180-34		282-317,319-374,376-550	419-433,441-489,491-508,510-537,539-558,561-575,577-667
c180-6		245-312,314-452	498-536,564-575,577-687,690-713
c180-7		206-312+314-583	
c180-22		439-585	
c180-5		207-312,314-583	
c180-41		14-23,25-132,171-277,279-317,319-357	
c180-38			624-628,633-635,637-657
c180-13			464-488,493-507
c180-14			605-628,633-648

Table S5. Themes of the e2of3A1, e1b3uA1 and e4adyA2 helix bundle domains and their respective sequence positions. Variations of many themes are shared between all three ECOD domains proteins. Some themes are shared only between two of the domains, and few are found only in one.

Theme ID	Position on e1j6oA1	Position on e2gzxA1	Position on e4p5uA1
**125**	0-35	2-37	1-35
**126**	0-45	2-46	1-45
**127**	0-62	2-57	1-56
**124**	39-95	44-97	-
**128**	54-103	56-105	-
**524**	61-138	60-140	47-140
**452**	73-132/73-138	75-140	74 141
**129**	80-139	86-141	-
**130**	84-154	75-153/86-156	-
**117**	100-139	102-140	101-139/102-141
**118**	105-154	102-160/107-153	104-159
**523**	149-183	147-186	-
**119**	151-191	153-192	-
**120**	151-201	153-204/153-210/155-204	-
**121**	151-250	151-253/169-253/188-253	148-257/167-256
**122**	176-250/193-250	169-253/188-253	187-256
**770**	-	39-99	38-98
**767**	-	149-208	148-207
**769**	-	216-254	218-262

Table S6. Themes of the e1j6oA1, e2gzxA1 and e4p5uA1 TIM barrels and their respective sequence positions. Variations of many themes are shared between all three proteins. Some themes are shared only between two of the proteins.

Theme ID	Position on e3ktzA1	Position on e3ctkA1
**10078**	3-42	3-37 / 3-43
**10081**	4-69	3-75
**10103**	45-80	45-80
**10079**	49-84 / 51-91	43-91 / 45-84
**10109**	51-140	46-142 / 47-142
**10104**	62-103	58-104
**10095**	62-130 / 66-140	58-132
**10105**	66-116	62-117
**10107**	81-177	80-176
**10096**	84-140	84-142
**10097**	98-140	98-142
**10098**	106-177	107-176
**10090**	118-177 / 123-181	119-176
**10099**	133-174	135-173
**10100**	133-188	135-188
**10075**	148-187	150-188 / 153-188
**10076**	152-196	150-195 / 151-196
**10077**	152-245	150-246
**10091**	154-233	153-234
**10089**	190-245 / 192-245	180-246
**10092**	191-234	191-234
**10080**	-	3-53
**10087**	-	3-89
**10101**	-	175-209

Table S7. Themes of the e3ktzA1 and e3ctkA1 domains from ribosome-inactivating proteins (RIP) 3KTZ and 3CTK and their respective sequence positions. Variations of many themes are shared between all three proteins. Some themes are shared only between two of the proteins.

Theme ID	Position on e1ulsB1	Position on e2ae2A1	Position on e3n74B1
**2939**	1-45 / 2-36 / 2-66 / 3-57 / 9-48	6-60 / 12-46	1-50 / 2-56 / 3-67 / 4-38 / 6-50
**2967**	46-86	54-98	48-92 / 55-89 / 56-91
**2968**	54-93 / 54-95	57-106 / 71-125	50-116 / 53-102 / 56-96 / 58-112 / 65-124
**2969**	64-139	83-149	72-143 / 76-146
**2960**	93-139	105-146	102-146
**2962**	99-167 / 104-183 / 111-169 / 130-185	105-171 / 109-196 / 131 196	95-213 / 96-176 / 98-173 / 99-163 / 102-167 / 102-213 / 104-176 / 106-213 / 109-213 / 114-171 / 114-213 / 120-213 / 122-214 / 126-187
**2963**	116-237 / 123-237	135-253	102-249
**2964**	141-185 / 148-183	152-196	149-213 / 151-213 / 154-214 / 156-213
**2965**	147-237 / 158-237 / 170-237	155-253 / 173-253	166-249
**2966**	188-237 / 191-240 / 192-237 / 195-240	194-253	214-249
**3034**	53-102	-	-
**3380**	61-137	-	63-144
**3397**	93-127	-	-
**3395**	128-181	-	-
**3396**	139-182	-	-
**3379**	146-237	-	156-249
**2972**	148-192	-	-
**3493**	181-237	-	-
**3117**	-	-	44-96
**3193**	-	63-98	56-90
**3314**	-	-	99-133
**3315**	-	-	100-186
**3472**	-	-	133-142
**3313**	-	-	138-186
**3316**	-	-	146-486

Table S8. Themes of the e1ulsB1, e2ae2A1 and e3n74B1 Rossmann-related domains and their respective sequence positions. Variations of many themes are shared between all three proteins. Some themes are shared only between two of the proteins.

Ten Sample Combinations of Themes for AMI-SMI Analysis (e2xyiA1)									
**Comb-1**	**Theme**	**14940-1**	**14946-1**	**14942-2**	**14954**	**14937**	**14816**	**14861-1**	**14951**
	**Residue**	14-53	55-120	126-170	176 220	227-265	271-309	314-353	356-415
**Comb-2**	**Theme**	**14940-1**	**14946-1**	**14942-2**	**14954**	**14939**	**14950**		
	**Residue**	14-53	55-120	126-170	176 220	227-309	314-408		
**Comb-3**	**Theme**	**14940-1**	**14946-2**	**14942-2**	**114813-2**	**14815-1**	**14861-1**	**14951**	
	**Residue**	14-53	58-91	126-170	176-240	244-309	314-353	356-415	
**Comb-4**	**Theme**	**14945**	**14942-2**	**14813-1**	**15218**	**15219**	**14861-1**	**14951**	
	**Residue**	16-120	126-170	176-231	230-270	281-315	314-353	356-415	
**Comb-5**	**Theme**	**14945**	**14942-2**	**14813-1**	**15221**	**15222**			
	**Residue**	16-120	126-170	176-231	230-330	336-407			
**Comb-6**	**Theme**	**14940-2**	**14946-1**	**14942-2**	**14813-1**	**14937**	**14816**	**14861-1**	**14951**
	**Residue**	21-55	55-120	126-170	176-231	227-265	271-309	314-353	356-415
**Comb-7**	**Theme**	**14940-2**	**14946-1**	**14942-2**	**14813-1**	**14939**	**14950**		
	**Residue**	21-55	55-120	126-170	176-231	227-309	314-408		
**Comb-8**	**Theme**	**14940-3**	**14946-2**	**14942-2**	**14813-1**	**14938**	**14955**	**14815-4**	
	**Residue**	21-59	58-91	126-170	176-231	227-287	283-341	343-406	
**Comb-9**	**Theme**	**14940-3**	**14946-2**	**14942-2**	**14813-1**	**15218**	**14816**	**14861-1**	**14951**
	**Residue**	21-59	58-91	126-170	176-231	230-270	271-309	314-353	356-415
**Comb-10**	**Theme**	**14940-3**	**14946-2**	**14942-2**	**14813-1**	**15221**	**15222**		
	**Residue**	21-59	58-91	126-170	176-231	230-330	336-407		

Table S9. Ten sample possible theme combinations of the e2xyiA1 propeller structure, filtered with 3-residue overlap and 8-residue gap restriction.

AMI	DEs Mode-1	DEs Mode-2	DEs Mode-3	DEs Mode-4	DEs Mode-5	DEs Mode-6	DEs Mode-7
**Minimum**	0.48	0.52	0.60	0.54	0.62	0.58	0.69
**Maximum**	0.67	0.69	0.73	0.71	0.80	0.72	0.91
**Average**	0.55	0.59	0.66	0.63	0.71	0.64	0.79
**SMI**	**DEs Mode-1**	**DEs Mode-2**	**DEs Mode-3**	**DEs Mode-4**	**DEs Mode-5**	**DEs Mode-6**	**DEs Mode-7**
**Minimum**	86	92	94	79	86	94	120
**Maximum**	130	140	135	113	111	128	160
**Average**	100	110	112	95	97	109	134

Table S10. AMI and SMI values for the correlation between the DEs of each of the seven slowest GNM modes and theme combinations, filtered with 5-residue overlap and 10-residue gap limit for e2xyiA1.

AMI	DEs Mode-1	DEs Mode-2	DEs Mode-3	DEs Mode-4	DEs Mode-5	DEs Mode-6	DEs Mode-7
**Minimum**	0.48	0.52	0.59	0.54	0.58	0.57	0.64
**Maximum**	0.68	0.73	0.77	0.76	0.81	0.74	0.94
**Average**	0.54	0.60	0.66	0.65	0.72	0.64	0.79
**SMI**	**DEs Mode-1**	**DEs Mode-2**	**DEs Mode-3**	**DEs Mode-4**	**DEs Mode-5**	**DEs Mode-6**	**DEs Mode-7**
**Minimum**	76	88	85	76	81	84	106
**Maximum**	130	153	148	123	111	132	160
**Average**	96	109	108	95	95	105	128

Table S11. AMI and SMI values for the correlation between the DEs of each of the seven slowest GNM modes and theme combinations, filtered with 5-residue overlap and 15-residue gap limit, for e2xyiA1.

Domain ID	DEs Mode-1	DEs Mode-2	DEs Mode-3	DEs Mode-4	DEs Mode-5	DEs Mode-6	DEs Mode-7
**e2xyiA1**	99	106	112	94	99	108	137
**e3emhA1**	90	114	N/A	100	108	117	65
**e1j6oA1**	104	95	78	95	81	102	76
**e2gzxA1**	92	100	82	100	83	74	90
**e4p5uA1**	126	73	89	80	103	101	87
**e3ktzA1**	75	77	97	85	92	84	96
**e3ctkA1**	92	74	94	72	77	89	81
**e1ulsB1**	53	67	76	88	72	98	90
**e2ae2A1**	71	111	81	65	86	100	81
**e3n74B1**	79	92	76	87	92	89	85

Table S12. The mean SMI values for individual modes of each of the in-depth set domains.

Domain ID	DEs Mode-1	DEs Mode-2	DEs Mode-3	DEs Mode-4	DEs Mode-5	DEs Mode-6	DEs Mode-7
**e2xyiA1**	127	128	133	109	111	124	160
**e3emhA1**	119	140	N/A	128	131	178	93
**e1j6oA1**	113	102	79	97	83	105	78
**e2gzxA1**	120	118	93	118	90	79	98
**e4p5uA1**	140	79	108	83	113	103	88
**e3ktzA1**	89	95	114	93	100	96	112
**e3ctkA1**	111	91	126	85	91	126	101
**e1ulsB1**	67	78	91	115	86	126	109
**e2ae2A1**	78	130	102	77	94	112	92
**e3n74B1**	100	115	98	105	115	106	119

Table S13. The maximum SMI values for individual modes of each of the in-depth set domains.

	PC1	PC2	PC3	PC4	PC5	PC6	PC7
**GNM 1**	0.13	0.11	0.86	-0.03	0.00	0.17	-0.27
**GNM 2**	-0.19	0.09	0.59	0.33	0.21	-0.08	0.03
**GNM 3**	0.82	-0.11	-0.04	0.02	0.09	0.01	-0.05
**GNM 4**	-0.35	0.03	0.16	0.52	0.14	0.04	0.30
**GNM 5**	-0.01	0.77	-0.19	0.03	-0.07	-0.01	-0.18
**GNM 6**	-0.05	-0.06	0.03	-0.07	0.53	0.22	0.28
**GNM 7**	0.04	0.27	-0.19	0.02	0.22	-0.26	0.40

Table S14. Pearson correlation coefficient between GNM modes and principal components (PCs).

T

D		t_1	…	t_j	…	t_l
	d_1	n_{11}	…	.	…	n_{1l}
	…	…		…		.
D	d_i	.		n_{ij}		.
	…	…		…		.
	d_k	n_{k1}	…	.	…	n_{kl}

Table S15. k × l contingency table of the overlaps between two clusterings D (dynamic elements) and T (themes) from a dataset consisting of N records.

Domain ID	DEs Mode-1	DEs Mode-2	DEs Mode-3	DEs Mode-4	DEs Mode-5	DEs Mode-6	DEs Mode-7
**e4kliA3**	0.89	0.70	0.71	0.55	0.31	0.28	0.32
**e2bcqA3**	1.00	0.57	0.44	0.25	0.70	0.50	0.62
**e4drxF1**	0.30	0.21	0.53	0.76	0.41	N/A	0.84
**e3ro3A1**	1.00	0.38	0.52	0.46	0.62	0.61	0.59

Table S16. Correspondence between Pfam entries and DEs in four possible ECOD domains via AMI analysis.
